# ELAVL1-KO Affects Steroid Synthesis in ACC Cell Line NCI-H295R and Reduces Colony-Forming Abilities

**DOI:** 10.3390/ijms27157001

**Published:** 2026-08-04

**Authors:** Max Brandau, Vanessa Kirsch, Dmitry Chernyakov, Laura-Sophie Landwehr, Silviu Sbiera, Bayram Edemir

**Affiliations:** 1 Klinik für Innere Medizin IV, Landeszentrum für Zell-und Gentherapie, Universitätsklinikum Halle (Saale), 06120 Halle (Saale), Germany; maxbrandau@googlemail.com (M.B.); vanessa_kirsch94@web.de (V.K.); dmitry.chernyakov@uk-halle.de (D.C.); 2 Department of Internal Medicine, Division of Endocrinology and Diabetes, University Hospital, University of Würzburg, 97070 Wurzburg, Germany; landwehr_l@ukw.de (L.-S.L.); sbiera_s@ukw.de (S.S.); 3 Lehrstuhl für Physiologie, Pathophysiologie und Toxikologie, ZBAF, Universität Witten/Herdecke, 58455 Witten, Germany

**Keywords:** adrenocortical carcinoma, ELAVL1, CRISPR/Cas9, steroidsynthesis, colony forming assays

## Abstract

Adrenocortical carcinoma (ACC) is a rare malignancy of the adrenal gland for which no curative treatment options exist in advanced stages. Analyzing the ACC cohort of The Cancer Genome Atlas, we identified that expression of the mRNA-stabilizing protein Embryonic-Lethal-Abnormal-Vision-Like RNA-Binding Protein 1 (ELAVL1) was negatively associated with patient survival. To investigate the functional role in ACC, we generated a CRISPR/Cas9-mediated ELAVL1 knockout (KO) in the ACC cell lines NCI-H295R and HAC15 and performed Next-Generation RNA Sequencing (NGS) to characterize transcriptomic changes. In NCI-H295R, NGS analysis revealed 3468 upregulated genes and 3458 downregulated genes in ELAVL1-deficient cells compared with controls. Kyoto Encyclopedia of Genes and Genomes (KEGG) pathway analysis identified significant enrichment of aldosterone biosynthesis-related genes and enriched downregulation of genes associated in with “pathways in cancer.” Functionally, ELAVL1-KO cells exhibited impaired colony-forming ability, indicating reduced proliferative or clonogenic potential. Steroid profiling of cell culture supernatants further demonstrated increased aldosterone synthesis and decreased cortisol and androgen production in the KO cells, consistent with the observed transcriptional changes. Given that hypercortisolism is an established negative prognostic factor in ACC, these data suggest that ELAVL1 may represent a potential therapeutic target worth further investigation for modulating steroidogenesis in ACC patients.

## 1. Introduction

Adrenocortical carcinoma (ACC) is a rare and aggressive malignancy arising from the adrenal cortex, characterized by an estimated annual incidence of 0.7 to 2 cases per million individuals [1,2,3]. While many cases are identified as incidentalomas during cross-sectional imaging, a subset of patients presents with symptoms of endocrine dysfunction, most frequently hypercortisolism [4,5]. Complete surgical resection currently remains the only potential curative intervention; however, the high risk of recurrence necessitates adjuvant therapy, typically involving mitotane [6]. In cases of advanced, unresectable, or metastatic disease, systemic treatment options—such as the combination of mitotane with etoposide, doxorubicin, and cisplatin (EDP-M)—are often utilized, though their efficacy is frequently limited by systemic toxicity and poor long-term outcomes [7,8]. Furthermore, the presence of overt hypercortisolism serves as a negative prognostic indicator [9,10], highlighting the critical importance of effective endocrine management alongside oncological therapy to mitigate metabolic complications and preserve patient quality of life.

In our analysis of the TCGA ACC cohort, higher *ELAVL1* expression was significantly associated with poorer patient survival. Long et al. had previously implicated *ELAVL1* in ACC through regulation by the long noncoding RNA ASB16-AS1 and showed that *ELAVL1* downregulation inhibits cell growth [11]. Because ELAVL1 is a well-studied mRNA-binding protein with oncogenic roles in many cancers [12], we investigated its function in ACC. ELAVL1, also known as HuR, binds AU-rich elements in the 3′-UTR of target mRNAs and increases their stability [13], thereby influencing genes involved in cancer and inflammation [14,15]. However, its role in ACC had not been defined. To address this gap, we generated a CRISPR/Cas9-mediated *ELAVL1* knockout in the ACC cell lines NCI-H295R and HAC15, and analyzed the resulting differentially expressed genes to elucidate the downstream molecular pathways affected by ELAVL1 depletion.

## 2. Results

### 2.1. ELAVL1 Expression Is Associated with Overall Survival in ACC Patients

**Figure 1:**
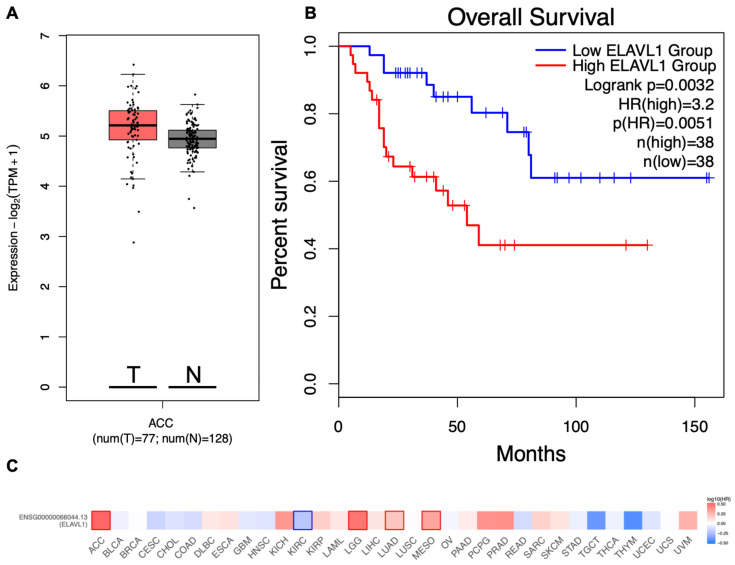
High *ELAVL1* expression predicts poor overall survival in ACC patients. (**A**) *ELAVL1* expression in 77 ACC samples (T, red) versus 128 normal adrenal samples (N, gray). (**B**) Kaplan–Meier overall survival analysis of ACC patients stratified by median *ELAVL1* expression (since one sample is the median sample, we have 38 samples above and 38 samples below the median) into low and high *ELAVL1* groups. (**C**) Hazard ratios for high *ELAVL1* expression across tumor entities. Framed tiles indicate *p* < 0.05.

In silico analysis of the TCGA ACC cohort using GEPIA2 showed slightly higher *ELAVL1* expression in tumor tissue than in healthy adrenal tissue (Figure 1A). Kaplan–Meier analysis revealed that ACC patients with higher *ELAVL1* expression had significantly poorer overall survival than those with lower expression (Figure 1B; HR = 3.2, *p* = 0.0032). Among the tumor entities analyzed, ACC showed the strongest association between *ELAVL1* expression and patient survival (Figure 1C). Significant associations were also observed in LGG, LUAD, MESO, and KIRC, although KIRC was the only entity in which higher *ELAVL1* expression correlated with improved survival.

### 2.2. Establishing a Functional ELAVL1 Knockout in NCI-H295R and HAC15 Cells

**Figure 2:**
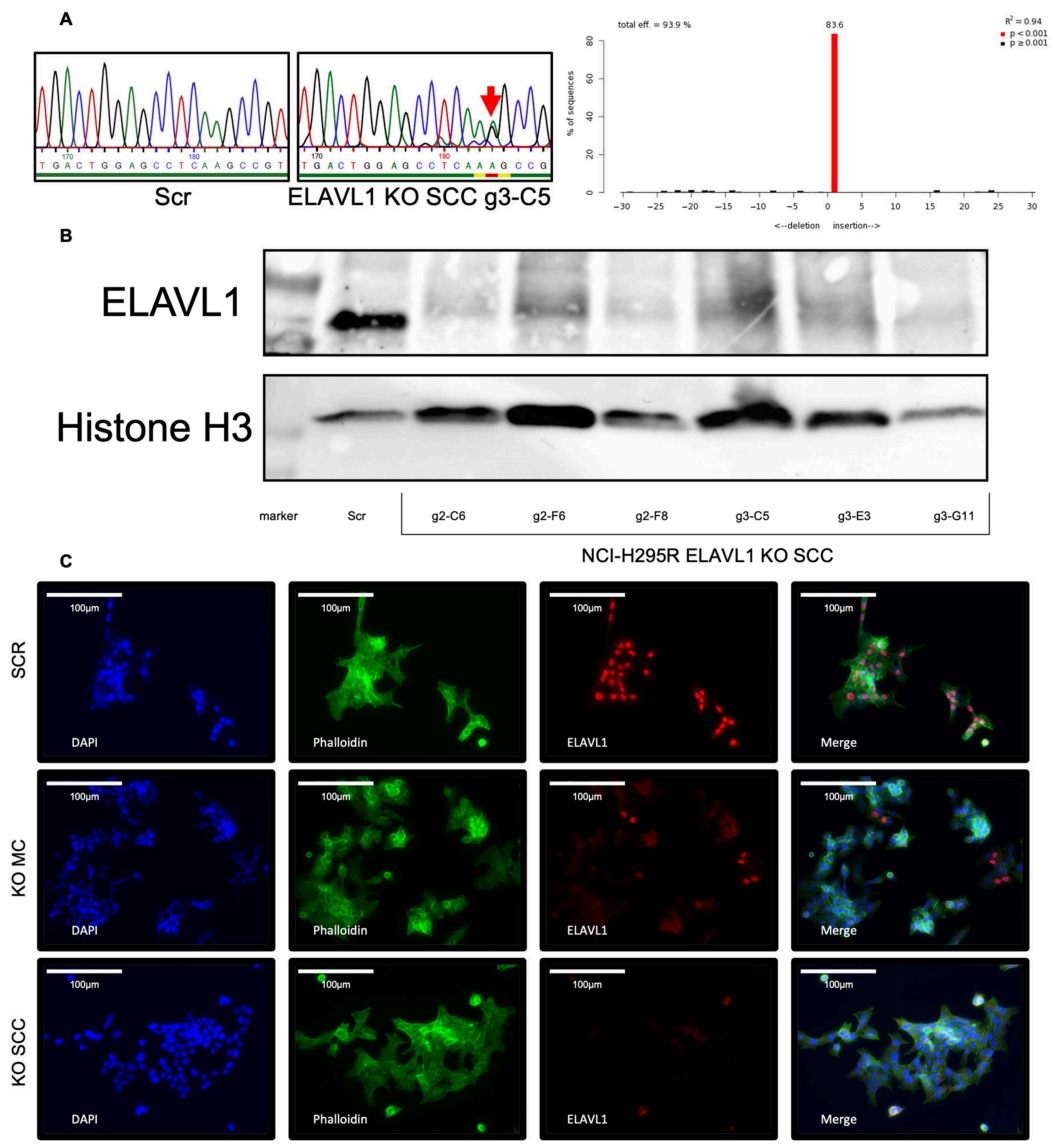
Establishment of a functional *ELAVL1* knockout in NCI-H295R cells. (**A**) DNA sequences of NCI-H295R scrambled control cells and an *ELAVL1* knockout single-cell clone. The red arrow indicates the insertion of one nucleotide, which was also confirmed by TIDE analysis on the right panel, resulting in a frameshift mutation. (**B**) Western blot analysis comparing ELAVL1 protein expression in NCI-H295R scrambled control cells and six different *ELAVL1* knockout single-cell clones. The lower panel shows a Western blot for Histone H3 as a loading control. (**C**) Immunofluorescence analysis of *ELAVL1* expression in NCI-H295R scrambled control cells (top), NCI-H295R mixed culture (KO-MC, middle), and one of six NCI-H295R *ELAVL1* knockout single-cell clones (bottom). ELAVL1 protein is enriched in the nucleus (shown in red). This pattern was not detectable in ELAVL1 knockout cells. Nuclei stained with DAPI are shown in blue; actin filaments were stained with Phalloidin (shown in green).

To investigate the role of *ELAVL1* in cellular function and its potential contribution to ACC tumorigenesis and progression, we generated an *ELAVL1* knockout in NCI-H295R and HAC15 cells using CRISPR/Cas9. Figure 2 shows exemplary validation of functional deletion of ELAVL1 in H295R cells. Similar validation was performed for HAC15 cells. Knockout efficiency was confirmed at the DNA level by Sanger sequencing and TIDE mutation analysis, revealing a frameshift mutation in *ELAVL1* (Chr.19 g.7981110_7981111insA/G; Figure 2A). Loss of ELAVL1 protein expression was validated by immunofluorescence and Western blot analysis, both of which showed no detectable ELAVL1 in the KO single-cell clones (Figure 2B,C). For further experiments, the six single-cell clones were pooled to reduce clonal effects.

### 2.3. Loss of ELAVL1 Results in Reduced Colony-Forming Abilities

**Figure 3:**
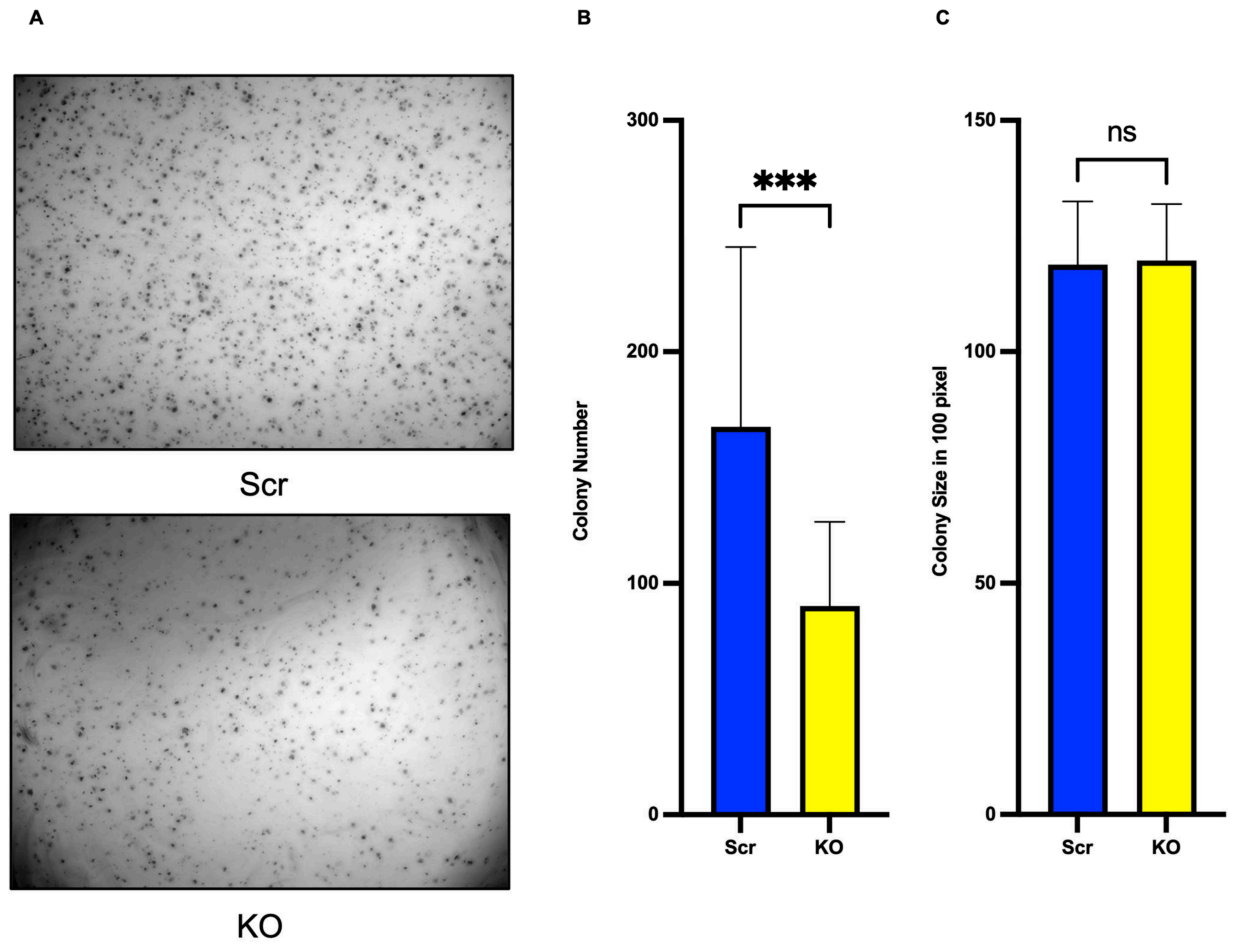
*ELAVL1*-KO in NCI-H295R leads to reduced colony-forming abilities. (**A**) Representative images from the soft agar colony formation assay of NCI-H295R scrambled control and *ELAVL1*-KO cells after 2 weeks, analyzed using ImageJ. (**B**,**C**) Quantification of colony number (**B**) and size (**C**) after 14 days. Scrambled control: *n* = 43 wells; ELAVL1-KO: *n* = 41 wells (4 independent experiments). Data represent mean ± SD. *** *p* < 0.001 (unpaired two-tailed Student’s *t*-test).

We performed live cell imaging to test if ELAVL1 deletion has an impact on cell proliferation. Although ELAVL1 was described to promote proliferation and migration in multiple tumor types, our results showed no significant impact on cell proliferation in ELAVL1-deficient cells. To further assess clonogenic and metastatic potential, soft agar colony formation assays were performed using *ELAVL1*-KO and scrambled control NCI-H295R cells. After 14 days, control cells formed significantly more colonies (mean = 167 ± 11.84; *n* = 43 wells) than *ELAVL1*-KO cells (mean = 90 ± 5.67; *n* = 41 wells) (Figure 3B; *** *p* < 0.001). Colony size did not differ between groups (*p* = 0.47) (Figure 3C).

The proliferation rate of the HAC15 cells was so low that we did not include them in the proliferation assay or colony-forming assay.

### 2.4. RNA-Seq Analysis Revealed Extensive Transcriptomic Changes upon ELAVL1 Knockout

**Figure 4:**
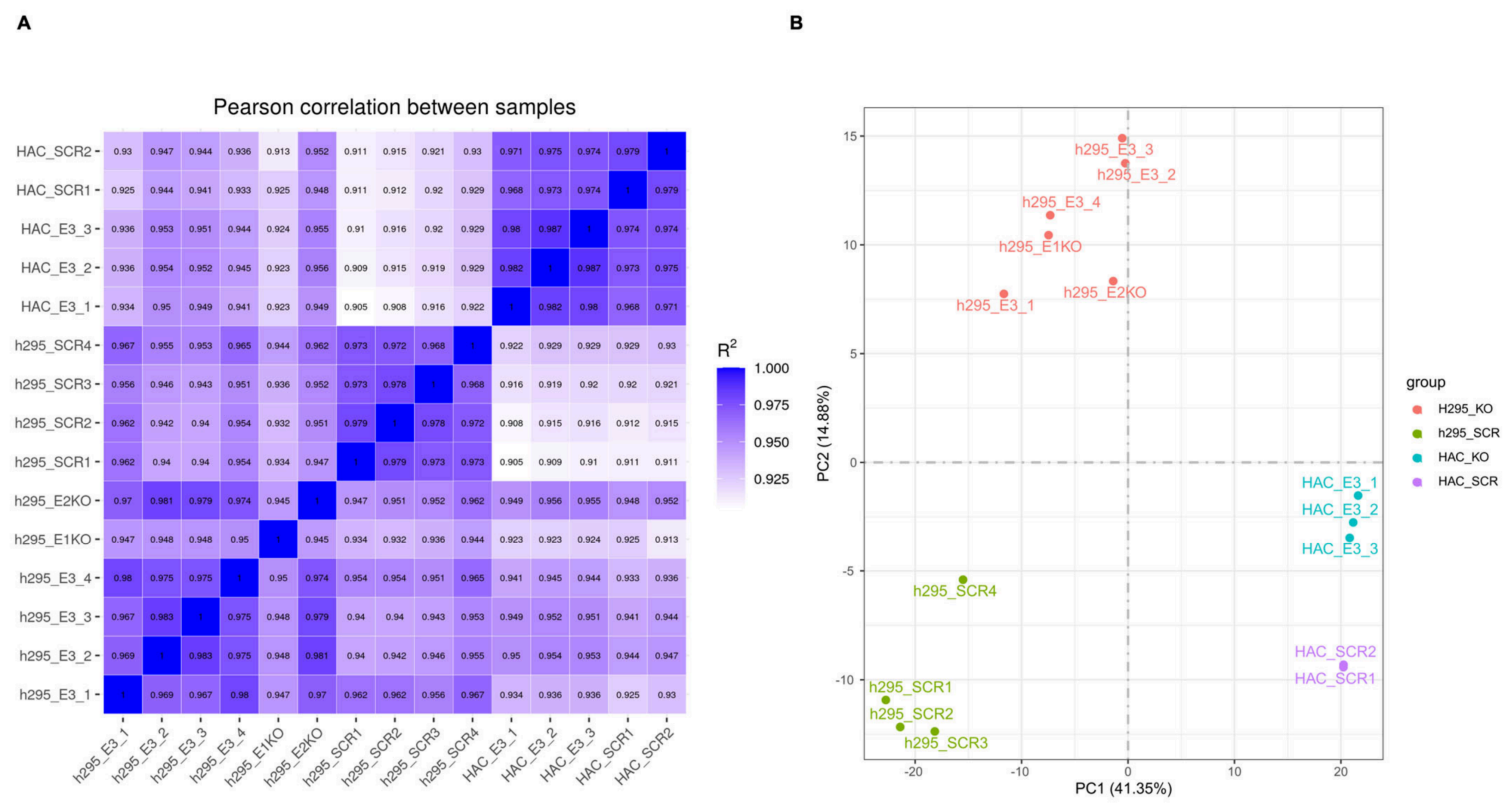
Transcriptomic analysis clearly separated *ELAVL1*-depleted HAC15 and H295R cell lines and control cells. (**A**) Pearson correlation matrix showing the similarity between global gene expression profiles of all analyzed biological replicates. (**B**) Principal component analysis (PCA) of RNA-seq data demonstrating the clustering of samples by cell line and knockout status, where PC1 and PC2 explain 41.35% and 14.88% of the total variance, respectively. Samples are color-coded by experimental group: H295_KO (red), h295_SCR (green), HAC_KO (teal), and HAC_SCR (purple).

Since ELAVL1 has an impact on mRNA stability, we performed gene expression profiling using next-generation sequencing in H295R and HAC15 cells. The CRISPR/Cas9-mediated targeting of *ELAVL1* in HAC15 cells led to efficient loss of protein expression in the bulk population, as confirmed by immunofluorescence, which indicated that approximately 80% of the cells behaved as knockouts. However, despite repeated attempts, we were unable to obtain viable single-cell clones, so that we in this case have rather a knockdown than a complete knockout. Nevertheless, RNA-seq was performed on the mixed HAC15 culture to assess the transcriptomic consequences of *ELAVL1* knockdown. In Figure 4, the Pearson correlation and principal component analysis of the selected groups illustrate the overall similarity and divergence in gene expression profiles between the conditions.

**Figure 5:**
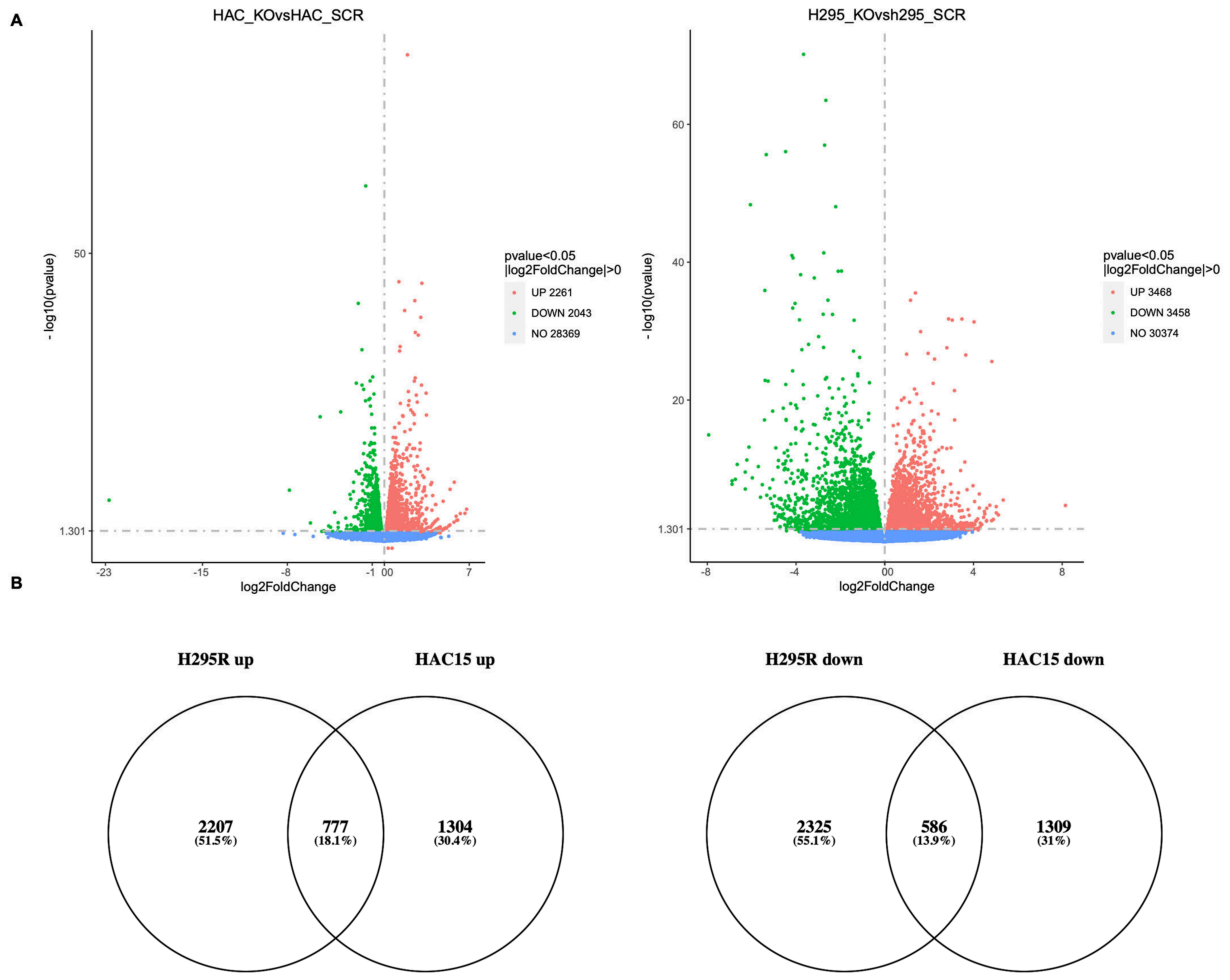
Differential gene expression analysis following *ELAVL1* knockout. (**A**) Volcano plots representing differentially expressed genes in HAC_KO vs. HAC_SCR and H295_KO vs. H295_Scr. Genes with a *p*-value < 0.05 are highlighted in red (upregulated) and green (downregulated), while non-significant genes are shown in blue. (**B**) Venn diagrams illustrating the overlap of significantly upregulated (left panel) and downregulated genes (right panel) between the HAC15 and H295R cell models, demonstrating common and cell-line-specific transcriptomic responses to *ELAVL1* depletion.

Volcano plots visualize widespread gene expression changes upon *ELAVL1* knockout (Figure 5A). Around 4300 genes were affected in HAC15 cells and around 6900 genes in H295R cells. To assess the overlap in transcriptional changes between the two cell types, we compared the lists of differentially expressed genes (*p* < 0.05). The Venn diagrams illustrate that while a core set of genes is commonly regulated—comprising 777 upregulated and 586 downregulated genes—a substantial proportion of the transcriptional response is cell-line specific (Figure 5B). The complete lists of differentially expressed genes and common or unique affected genes are provided as Supplemental Excel File S1.

**Figure 6:**
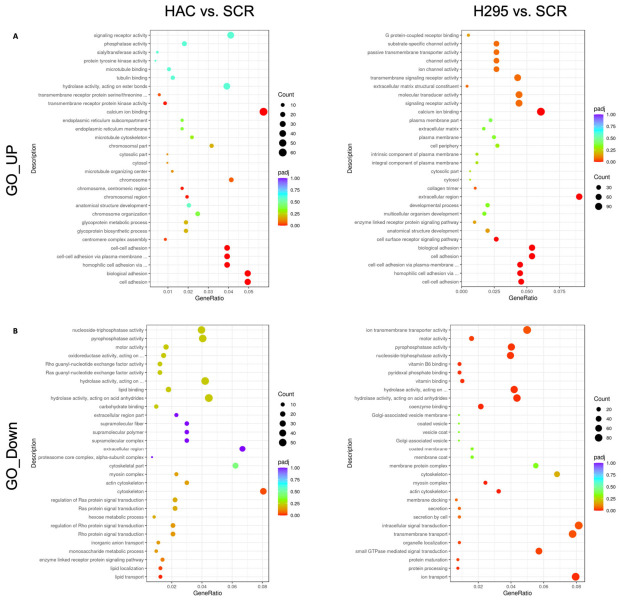
GO functional enrichment analysis of *ELAVL1* knockout-affected genes in HAC15 and H295R cells. Enriched GO terms were identified among upregulated genes (**A**) and downregulated genes (**B**) in HAC15 and H295R *ELAVL1* knockout cells relative to scrambled control. Bubble size indicates the number of genes associated with each term; the x-axis shows gene ratio, and the colored scale bar reflects the adjusted *p*-value, indicating the significance level.

In the next step, we performed functional annotation enrichment using gene ontology (GO) classification and KEGG signaling pathway analysis separately for the list of up- and downregulated genes to identify enriched GO terms or KEGG signaling pathways that are associated with differentially expressed genes. Figure 6 shows the enriched GO terms that are associated with the up- or downregulated genes in HAC15 and H295R cells.

Since there are differences within the affected genes, this is also reflected by the enriched GO terms. Several of the GO terms are common in both cell lines. For example, “motor activity” or “myosin complex” within the upregulated terms and “calcium ion binding” or “cell–cell adhesion” within the list of downregulated genes. This implicates that *ELAVL1* activity might be involved in these kinds of functions in both cell types.

**Figure 7:**
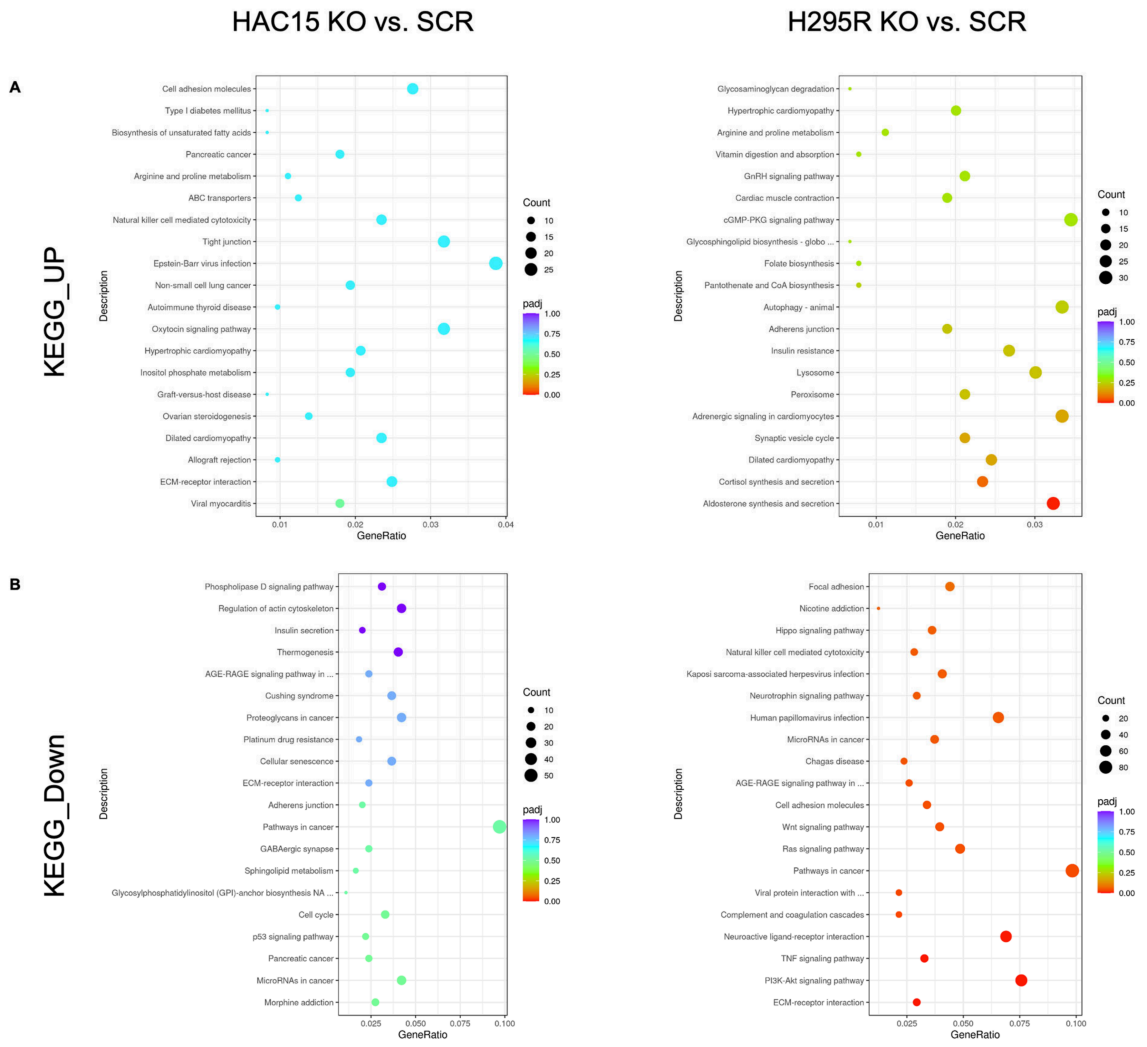
KEGG pathway enrichment analysis of *ELAVL1* knockout-affected genes in HAC15 and H295R cells. Enriched KEGG pathways were identified among upregulated genes (**A**) and downregulated genes (**B**) in HAC15 and H295R *ELAVL1* knockout cells relative to scrambled control. Bubble size indicates the number of genes associated with each pathway, the x-axis shows gene ratio, and bubble color and scale bar reflect the adjusted *p*-value.

These results highlight that while *ELAVL1* serves as a consistent regulator of gene expression, its functional impact is highly cell- and probably context-dependent, reflecting distinct regulatory networks between H295R and HAC15 cells. Despite the observed cell-line-specific transcriptional responses, we identified *CYP11B2*—a key enzyme in aldosterone biosynthesis—as a consistently upregulated gene in HAC15 and H295R knockout cells (Figure 7).

In the same way, we analyzed for enriched KEGG signaling pathways (Figure 7). In HAC15, ELAVL1 knockout showed a stronger emphasis on signaling, immune-related, and hormone/metabolic related pathways, including cell adhesion molecules, tight junctions, natural killer cell-mediated cytotoxicity, cytokine signaling, and pancreatic cancer-related pathways in the upregulated set, while downregulated genes were associated with phospholipase D, insulin secretion, thyroid hormone, ECM-receptor interaction, and p53-related pathways. In contrast, in H295R cells, *ELAVL1* knockout displayed a broader shift toward steroidogenic, endocrine, and stress-response biology, with association of the upregulated genes in GnRH, aldosterone and cortisol synthesis and secretion, cAMP/AMPK signaling, insulin resistance, and adipocytokine-related pathways, and downregulated genes playing a role in focal adhesion, PI3K-Akt, Ras, Hippo, and ECM-receptor interaction pathways. No overlap was present between HAC15 and H295R cells related to KEGG signaling pathways. Interestingly, loss of ELAVL1 function in H295R cells was associated with enriched upregulation of genes that are associated with “aldosterone synthesis and secretion” (Figure 7B).

### 2.5. Association of ELAVL1-Dependent Gene Profiles with Patient Survival in NCI-H295R

To better contextualize the results of the in silico analysis performed at the beginning of the study, we conducted an unbiased analysis using the 10 most strongly up- and downregulated genes as a gene signature to assess their concordance with the initial in silico findings.

**Figure 8:**
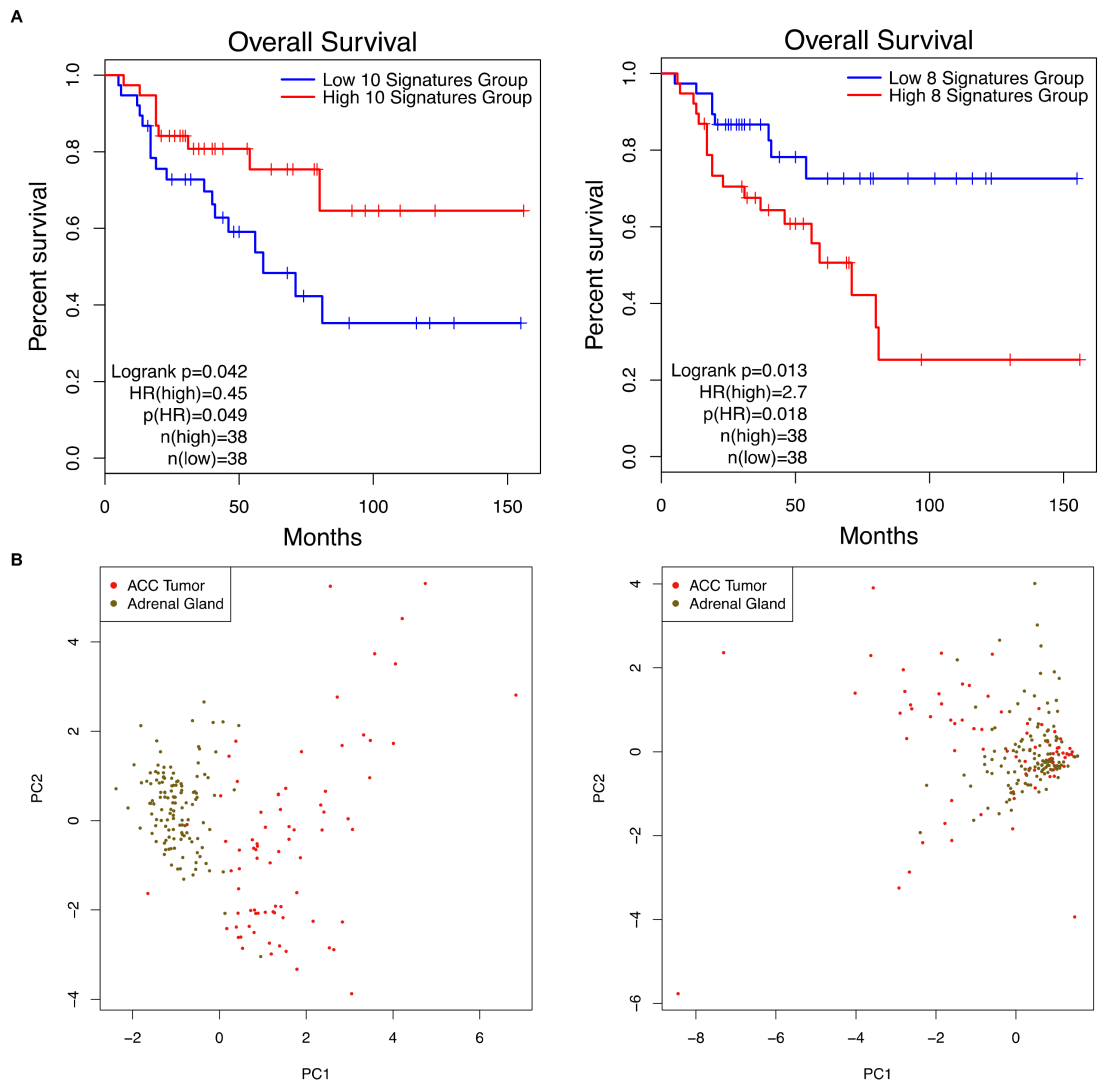
Gene signatures of the top 10 down- and upregulated genes correlate with ACC patient overall survival. (**A**) Signatures of the top 10 down- (left) and top 8 upregulated (right) genes correlate significantly with ACC patients’ overall survival. *MAB21L4* and *STRIT1* are neglected due to missing TCGA data. (**B**) Dimensionality reduction plots comparing ACC tumor samples and healthy adrenal tissue samples regarding the expression profile of the top 10 down- (left) and top 8 upregulated (right) genes in NCI-H295R *ELAVL1*-KO cells.

The gene signature of the downregulated genes (Table 1) with the clinical data from patients in the TCGA ACC cohort showed a significant favorable correlation between expression level and patients’ overall survival (Figure 8A). Interestingly, the gene signature of the upregulated genes has an unfavorable impact on the overall survival of the patients (Figure 8A). The downregulated gene signatures are also capable of distinguishing the ACC samples from the healthy adrenal tissue samples (Figure 8B).

### 2.6. Analysis of Gene Signatures of the Top 10 Up- and Downregulated Genes in HAC15 with Respect to Overall Survival in ACC Patients

**Figure 9:**
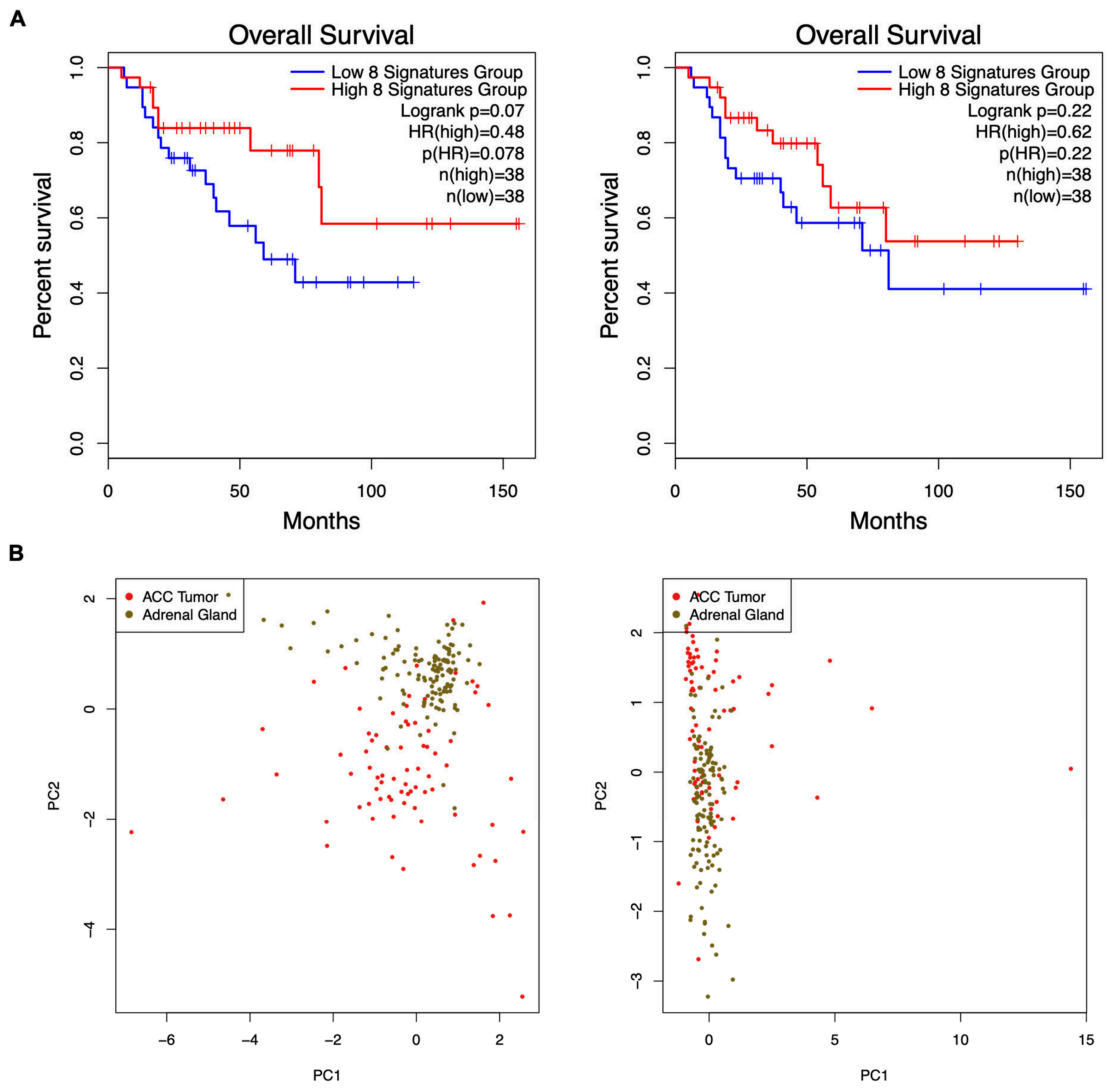
Signature of the top 8 down- and upregulated genes in the HAC15 *ELAVL1*-KO bulk population shows no correlation with ACC patient overall survival. (**A**) Gene signatures of the top 8 down- (left) and top 8 upregulated (right) genes do not correlate with ACC patients’ overall survival. *ZP3P2*, *RUBCNL*, *CYRIA* and *FDX2* are neglected due to missing TCGA data. (**B**) Dimensionality reduction plots comparing ACC tumor samples and healthy adrenal tissue samples regarding the expression profile of the top 8 up- and top 8 downregulated genes in HAC15 *ELAVL1*-KO bulk population.

In contrast to NCI-H295R, the gene signature derived from the top 10 down- and upregulated genes in HAC15 (Table 2) did not show a significant correlation with overall survival in ACC patients (Figure 9A). But in the dimensional reduction analysis, the signature based on the top eight downregulated genes clearly separated healthy adrenal tissue from tumor tissue according to expression levels (Figure 9B). For the top eight upregulated genes, the separation was less distinct than for the downregulated genes, although a similar tendency was observed (Figure 9B).

Multivariate analysis is shown as Supplemental Figure S1 and as Supplemental Table S2.

### 2.7. Deregulation of Steroid Synthesis Associated Genes

**Figure 10:**
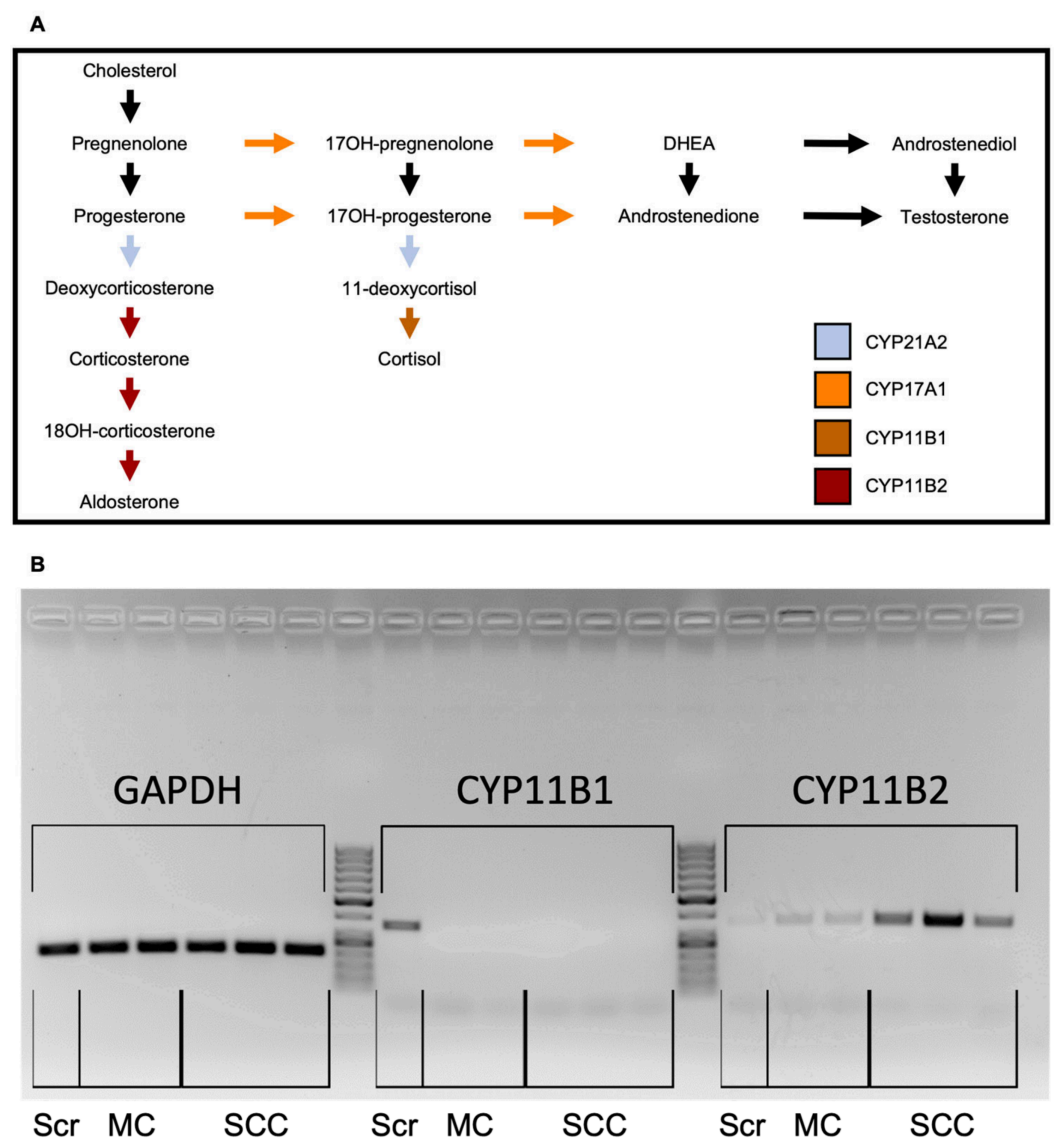
*ELAVL1*-KO leads to changes in gene expression involved in steroid synthesis in NCI-H295R. (**A**) Scheme of adrenal steroid hormone synthesis (modified from Miller et al. [16]). Different colored arrows mark reactions that are catalyzed by enzymes that are deregulated in NCI-H295R *ELAVL1*-KO cells according to NGS data. (**B**) PCR of *CYP11B1* and *CYP11B2* using cDNA of NCI-H295R Scr, NCI-H295R *ELAVL1*-KO mixed cultures, and three samples of NCI-H295R *ELAVL1*-KO pooled SCC. *GAPDH* was amplified as a control.

As mentioned previously, KEGG analysis of upregulated genes identified 29 genes associated with aldosterone synthesis and secretion, all of which were significantly upregulated. Among these were *CYP11B2* and *CYP21A2*. *CYP11B2*, also known as aldosterone synthase and responsible for catalyzing the final three steps in aldosterone synthesis, was upregulated with a log_2_ fold change of 3.77, while *CYP21A2* (21-hydroxylase), an enzyme involved in both aldosterone and cortisol synthesis, was upregulated with a log_2_ fold change of 1.87. *CYP11B1*, which catalyzes the conversion of 11-deoxycortisol to cortisol, showed a log_2_ fold change of −4.46, and *CYP17A1*, important not only in cortisol but also in androgen synthesis, was downregulated with a log_2_ fold change of −1.43 (Table 3; Figure 10A). To confirm the deregulation of genes associated with steroid synthesis, RNA was isolated from NCI-H295R Scr, NCI-H295R ELAVL1-KO mixed cultures, and NCI-H295R ELAVL1-KO SCC and PCR was performed. Using primers specific for *CYP11B1* and *CYP11B2*, no amplifiable *CYP11B1* transcripts were detected in the KO mixed cultures and KO SCC, whereas *CYP11B2* transcripts clearly increased after ELAVL1-KO. Amplification of GAPDH transcripts as a control showed comparable signal intensity across all samples (Figure 10B). In the NGS data of the HAC15 ELAVL1-KO bulk population, *CYP11B2* was also upregulated, with a log_2_ fold change of 2.8. In contrast, *CYP17A1* was upregulated in HAC15 cells as well, with a log_2_ fold change of 1.25. *CYP21A2* and *CYP11B1* were not significantly deregulated in the HAC15 NGS data.

Although KEGG analysis identified 29 genes associated with aldosterone synthesis and secretion as upregulated, we focused subsequent analyses on four genes directly involved in the steroidogenic pathway, as the remaining genes were predominantly related to G protein-coupled receptor signaling. This approach was chosen to specifically assess the impact of ELAVL1 knockout on phenotypic cell function in NCI-H295R cells.

**Figure 11:**
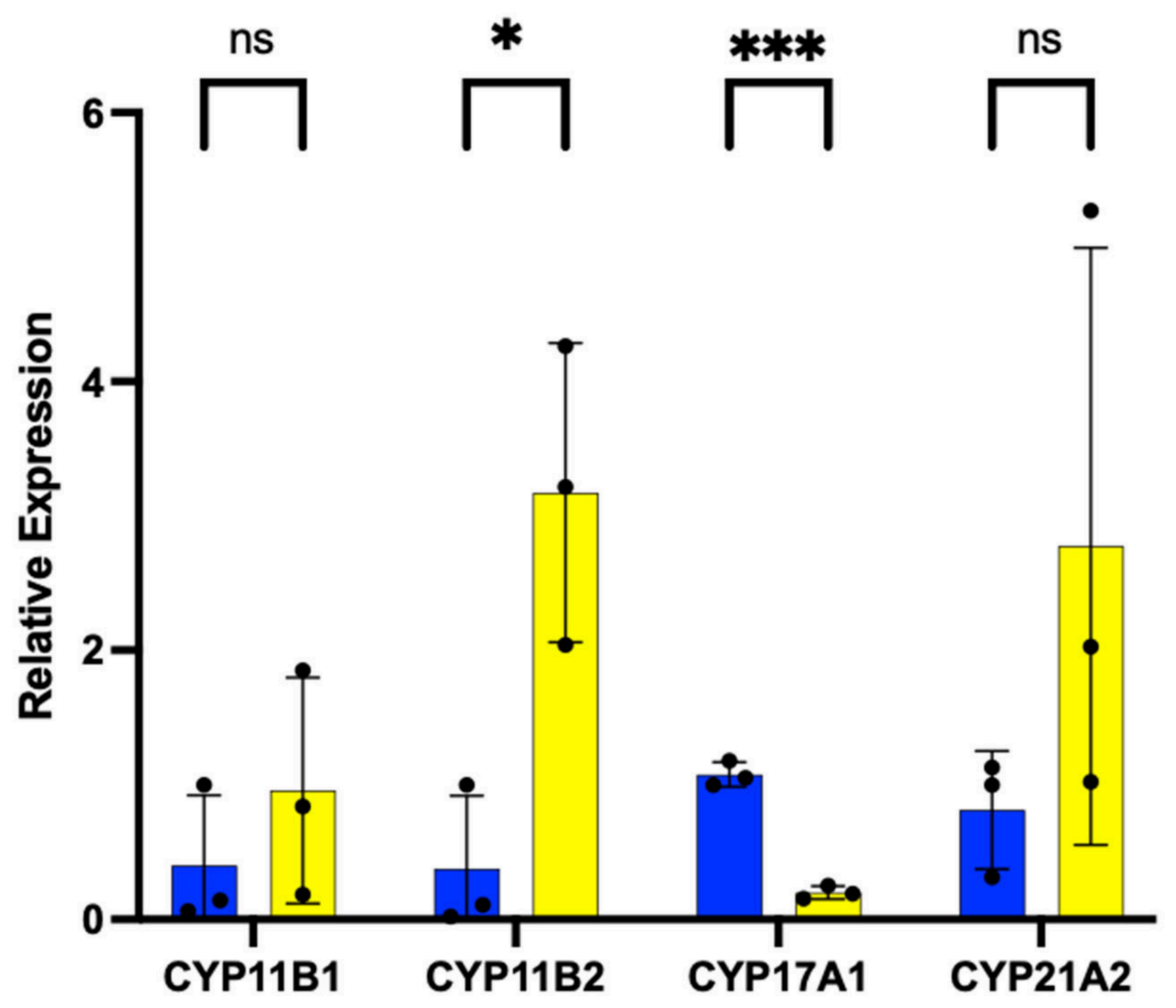
qPCR of deregulated genes in steroid synthesis in NCI-H295R KO cells. Statistically significant changes in expression could only be confirmed for *CYP11B2* and *CYP17A1*. Blue = Scr; yellow = ELAVL1-KO. Error bars represent ± standard deviation. Significance levels: * *p* < 0.05; *** *p* < 0.001. Statistics: unpaired two-tailed Student’s *t*-test. cDNA was used from 3 independent experiments.

To validate the RNA-seq results, qPCR was performed (Figure 11). In contrast to the NGS data, only *CYP11B1* and *CYP17A1* showed significant deregulation by qPCR. Deregulation of *CYP11B1* and *CYP21A2* could not be confirmed.

### 2.8. Measurement of Steroid Hormone Concentrations in Cell Culture Supernatants Confirms NGS Data

Since we observed changes in the steroid synthesis pathway, we tested whether altered expression of genes encoding steroidogenic enzymes affects hormone production by measuring concentrations of 13 steroid hormones in cell culture supernatants using mass spectrometry.

Focusing on the aldosterone synthesis pathway, we analyzed progesterone, 11-deoxycorticosterone, corticosterone, and aldosterone levels in NCI-H295R *ELAVL1* knockout (KO) cells and control cells. Consistent with gene expression data, all measured steroids in this pathway were elevated in KO cell supernatants.

**Figure 12:**
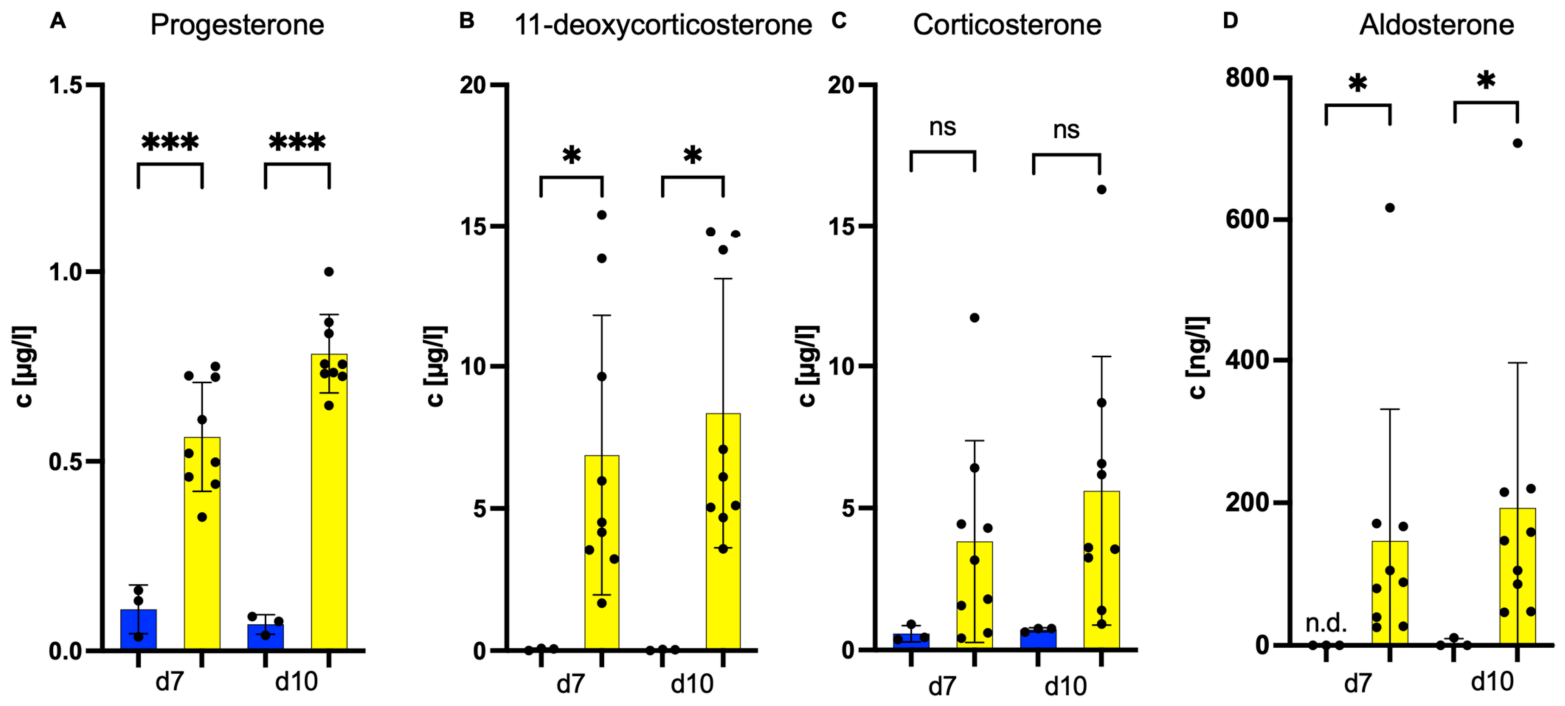
Loss of functional ELAVL1 leads to enhanced production of adrenal mineralocorticoids (**A**–**D**) NCI-H295R-Scr (blue) compared to *ELAVL1*-KO (yellow): concentrations of different steroids in mineralocorticoid synthesis in cell culture supernatants on day 7 (d7) and day 10 (d10). Columns represent mean ± SD (error bars); *, *p* < 0.05; *** *p* < 0.001, unpaired two-tailed Student’s *t*-test; n.d. = not detectable. Normalized to 60,000 seeded cells.

11-deoxycorticosterone, synthesized from progesterone by *CYP21A2*, was significantly increased in KO supernatants compared to controls (day 7: 6.879 µg/L ± 5.73 vs. 0.049 µg/L ± 0.043, *p* = 0.04; day 10: 8.36 µg/L ± 4.73 vs. 0.027 µg/L ± 0.024, *p* = 0.01; Figure 12B). Corticosterone, produced from 11-deoxycorticosterone by *CYP11B2*, was also higher in KO cells (day 7: 3.823 µg/L ± 3.55 vs. 0.575 µg/L ± 0.29; day 10: 5.608 µg/L ± 4.73 vs. 0.716 µg/L ± 0.07; Figure 12C), although these differences did not reach statistical significance (*p* = 0.16 and *p* = 0.11, respectively).

In contrast, aldosterone levels were significantly higher in KO cells (day 7: 146.7 ng/L ± 184.49 vs. not detectable; day 10: 192.67 ng/L ± 203.56 vs. 3.567 ng/L ± 6.18; *p* = 0.04 and *p* = 0.02; Figure 12D). These findings support the NGS data indicating upregulation of *CYP11B2*.

Across all conditions, hormone concentrations were higher on day 10 than on day 7. As the culture medium was refreshed every three days, this increase is likely attributable to cell proliferation and the resulting higher number of hormone-producing cells.

**Figure 13:**
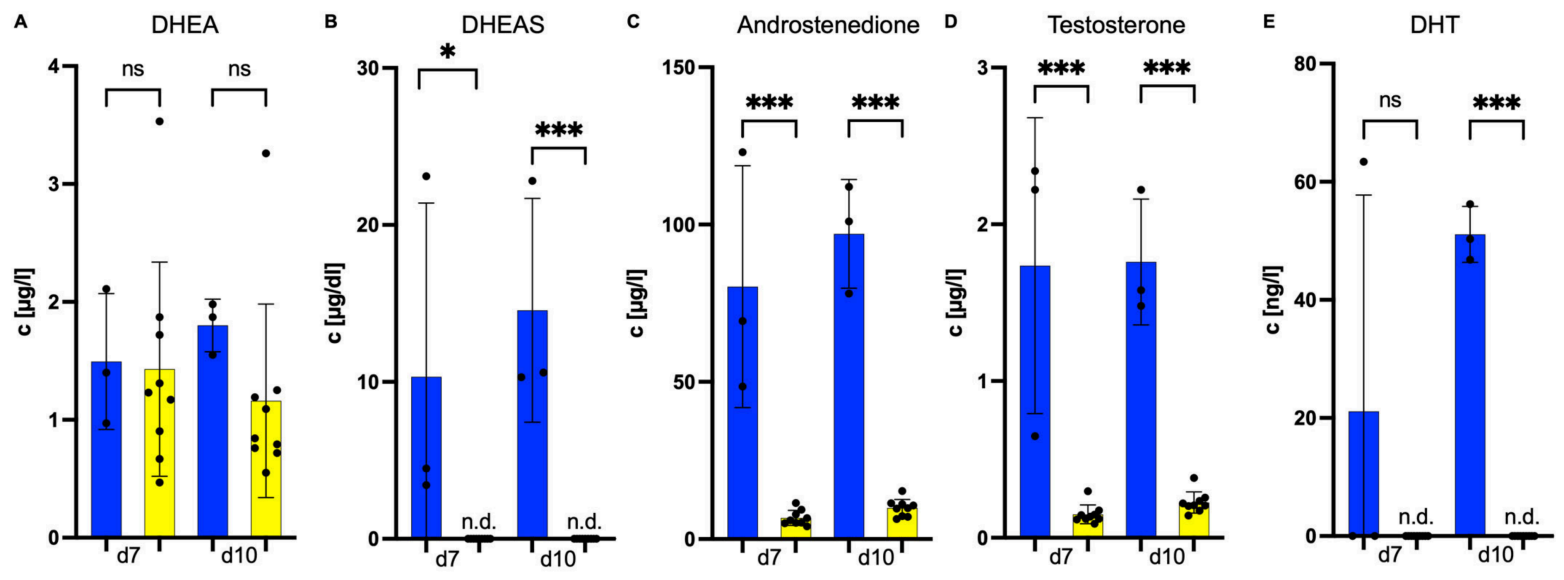
Reduced androgen synthesis in NCI-H295R *ELAVL1*-KO cells. (**A**–**E**) NCI-H295R Scr (blue) compared to *ELAVL1*-KO (yellow): concentrations of different steroids in androgen synthesis measured in cell culture supernatants on day 7 and day 10. Columns represent mean ± SD (error bars); *, *p* < 0.05; *** *p* < 0.001, unpaired two-tailed Student’s *t*-test; n.d. = not detectable. Normalized to 60,000 seeded cells.

**Figure 14:**
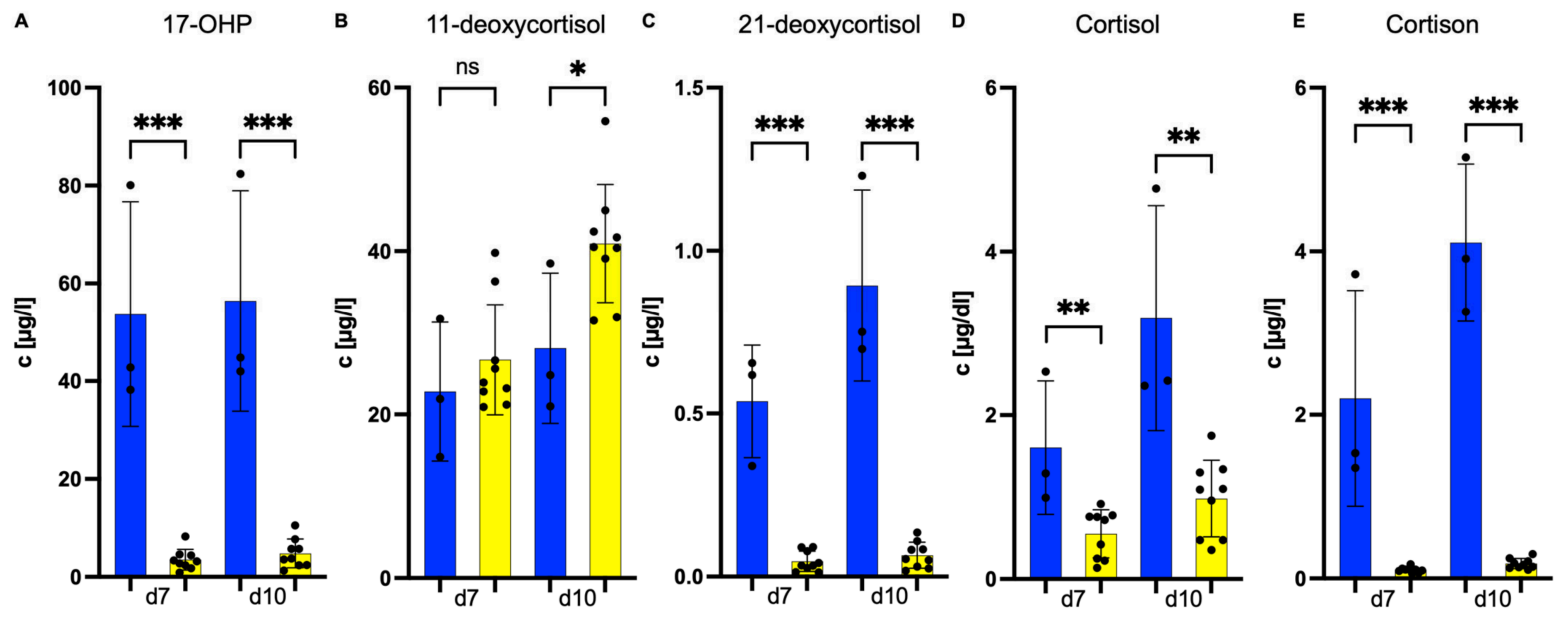
*ELAVL1*-KO in NCI-H295R is associated with reduced glucocorticoid synthesis. (**A**–**E**) NCI-H295R Scr (blue) compared to *ELAVL1*-KO (yellow): concentrations of different steroids in glucocorticoid synthesis measured in cell culture supernatants on day 7 and day 10. Columns represent mean ± SD (error bars); * *p* < 0.05; ** *p* < 0.01; *** *p* < 0.001, unpaired two-tailed Student’s *t*-test. Normalized to 60,000 seeded cells.

To assess changes in androgen synthesis, we measured concentrations of 17-hydroxyprogesterone (17-OHP), dehydroepiandrosterone (DHEA), dehydroepiandrosterone sulfate (DHEAS), androstenedione, testosterone, and dihydrotestosterone (DHT) (Figure 13 and Figure 14A).

17-OHP, produced from progesterone by CYP17A1, was markedly reduced in ELAVL1-KO cell supernatants (day 7: 3.505 µg/L ± 2.13; day 10: 4.781 µg/L ± 2.94) was significantly decreased compared to control cells (day 7: 53.7 µg/L ± 22.98; day 10: 56.4 µg/L ± 22.56), (*p* < 0.001 for both time points; Figure 13A). This finding supports downregulation of *CYP17A1*. Consistently, progesterone concentrations were significantly higher in KO cells (day 7: 0.564 µg/L ± 0.14; day 10: 0.784 µg/L ± 0.1) than in controls (day 7: 0.110 µg/L ± 0.06; day 10: 0.070 µg/L ± 0.03; *p* < 0.001 for both; Figure 12A), indicating substrate accumulation upstream of CYP17A1. Thus, despite increased substrate availability, 17-OHP production is reduced in *ELAVL1*-KO cells.

CYP17A1 also catalyzes the conversion of 17-OHP to androstenedione and 17α-hydroxypregnenolone to DHEA. In line with postulated reduced CYP17A1 activity, androstenedione levels were significantly decreased in KO supernatants (*p* < 0.001 for both day 7 and day 10; Figure 13C). In contrast, DHEA concentrations were not significantly altered between KO and control cells (*p* = 0.91 and *p* = 0.23, respectively; Figure 13A).

Testosterone and dihydrotestosterone levels were also markedly lower in *ELAVL1*-KO cells. Testosterone, synthesized from androstenedione by 17β-hydroxysteroid dehydrogenase type 5 (17β-HSD5), was reduced in KO supernatants (day 7: 0.149 µg/L ± 0.06; day 10: 0.225 µg/L ± 0.07) compared to controls (day 7: 1.736 µg/L ± 0.94; day 10: 1.76 µg/L ± 0.40; *p* < 0.001 for both; Figure 13D). As *17β-HSD5* expression is not altered in *ELAVL1*-KO cells according to NGS data, the reduction in testosterone is most likely due to limited substrate availability, consistent with decreased androstenedione levels.

We next examined the cortisol synthesis pathway by measuring 11-deoxycortisol, 21-deoxycortisol, cortisone, and cortisol concentrations (Figure 14). This pathway shares the initial conversion of progesterone to 17-OHP by CYP17A1 with androgen synthesis, where we previously observed reduced 17-OHP levels in *ELAVL1*-KO cells due to *CYP17A1* downregulation.

Despite decreased 17-OHP in KO supernatants, 11-deoxycortisol (formed from 17-OHP by CYP21A2) showed only modest changes. Levels were only significantly elevated on day 10 in KO cells (40.93 µg/L ± 7.23) compared to controls (28.1 µg/L ± 9.21; *p* = 0.03; Figure 14B). In contrast, 21-deoxycortisol (produced from 17-OHP by CYP11B1) was markedly reduced in KO supernatants (day 7: 0.047 µg/L ± 0.03; day 10: 0.065 µg/L ± 0.04) versus controls (day 7: 0.54 µg/L ± 0.17; day 10: 0.89 µg/L ± 0.29; *p* < 0.001 for both; Figure 14C), which is consistent with NGS data showing *CYP11B1* downregulation.

Cortisol (synthesized from 11-deoxycortisol by CYP11B1) and cortisone (formed from cortisol by 11β-hydroxysteroid dehydrogenase 2) were also significantly lower in KO cells. Cortisol levels were 0.55 µg/dL ± 0.29 (day 7) and 0.98 µg/dL ± 0.47 (day 10) in KO supernatants versus 1.6 µg/dL ± 0.81 (day 7) and 3.2 µg/dL ± 1.37 (day 10) in controls (*p* = 0.0056 and *p* = 0.0013; Figure 14D). Cortisone levels were similarly reduced (KO: day 7: 0.11 µg/L ± 0.03; day 10: 0.18 µg/L ± 0.06 vs. control: day 7: 2.2 µg/L ± 1.32; day 10: 4.11 µg/L ± 0.96; *p* < 0.001 for both; Figure 14E).

In summary, steroid measurements in cell culture supernatants validate the functional consequences obtained from NGS results. *ELAVL1*-KO cells exhibit upregulated *CYP11B2*, evidenced by elevated corticosterone and aldosterone levels. Similarly, *CYP21A2* upregulation correlates with increased 11-deoxycorticosterone and modestly higher 11-deoxycortisol. Conversely, *CYP17A1* downregulation is confirmed by reduced 17-OHP and androstenedione, while *CYP11B1* downregulation aligns with lower cortisol concentrations.

## 3. Discussion

The mRNA-stabilizing protein ELAVL1 has been implicated in promoting tumorigenesis and tumor progression across various tumor entities [12,17], though its specific role in ACC remains unexplored. Analysis of TCGA data revealed a significant negative correlation between *ELAVL1* expression and patient overall survival in ACC, which may suggest a potential involvement in disease progression. Furthermore, loss of functional ELAVL1 protein appeared to be associated with reduced colony-forming ability in the ACC cell line NCI-H295R, which has previously also been described after an *ELAVL1* knockdown in the oral cancer cell line HSC-3 [18]. As colony formation is considered relevant to metastatic potential and a hallmark of carcinogenesis [19], ELAVL1 loss might impair this process, though this requires further investigation. Consistent with prior findings by Long et al. [11] showing that *ELAVL1* downregulation inhibits cell growth in NCI-H295R, these observations hint at possible effects on at least two hallmarks of carcinogenesis in this model. While no approved ELAVL1 inhibitors are currently available for clinical use, recent research on HuR inhibitors has identified several candidate compounds [20,21,22]. For instance, the small-molecule inhibitor KH-3 suppressed breast cancer growth and metastasis in vitro and in xenograft models [23], raising the possibility—albeit untested—that it could target ELAVL1 in NCI-H295R cells. In further experiments, it would be interesting to see if pharmacological ELAVL1 inhibition in NCI-H295R leads to the same effects of impaired colony-forming abilities shown in this study.

When comparing differentially expressed genes in HAC15 *ELAVL1* knockdown cells with those in NCI-H295R ELAVL1 knockout cells, we identified a common set of 777 upregulated and 586 downregulated genes, indicating a shared *ELAVL1*-dependent transcriptional program between both cell lines. At the same time, the majority of deregulated genes were specific to each cell line, suggesting that the transcriptomic consequences of *ELAVL1* loss are strongly context-dependent and shaped by the distinct molecular backgrounds of HAC15 and NCI-H295R cells.

Our analysis of ACC patient survival in relation to the NCI-H295R *ELAVL1*-KO gene signatures indicates that ELAVL1-dependent transcriptomic changes may be linked to clinically relevant patterns. In this dataset, higher expression of the top 10 genes downregulated upon *ELAVL1* knockout in NCI-H295R cells was associated with improved survival, whereas increased expression of the corresponding top 10 upregulated genes correlated with decreased overall survival. However, these associations do not establish causality, and additional functional studies will be required to clarify the contribution of individual genes to ACC biology. In contrast, the top 10 up- and downregulated genes identified in HAC15 *ELAVL1* knockdown cells did not show a clear association with ACC patient survival, which is in line with our broader observation that the transcriptional consequences of *ELAVL1* perturbation differ between the two cell models and may not uniformly translate to the clinical setting.

High expression of *AADAC*, the most strongly downregulated gene in NCI-H295R ELAVL1-KO cells, has recently been reported as a prognostic marker in gastric cancer, where its expression correlates with tumor stage [24]. Moreover, a 2022 study demonstrated that *AADAC* overexpression in colorectal carcinoma promotes metastatic colonization of the liver and the growth of liver metastases [25]. *FAT3*, the second most strongly downregulated gene in our ELAVL1-KO model, has likewise been implicated in cancer biology; in lung cancer, ELAVL1 has been shown to regulate *FAT3*, and *FAT3* expression correlates with tumor size, lymph node metastasis, and clinical stage [26]. *SLPI*, the next gene among the top 10 downregulated candidates, is upregulated in multiple tumor entities and has been described as a negative prognostic factor, for example in breast, ovarian, and non-small cell lung cancer [27,28,29]. In addition, *CXCL6*, *LYPD1*, *PLA2G2A*, *ITGA4*, and *PI3* are overexpressed in several other tumor types and have been associated with metastatic progression and poor patient outcome [30,31,32,33]. Collectively, the reported adverse roles of these genes in other cancers suggest that the observed positive association of the corresponding NCI-H295R ELAVL1-KO “downregulated” signature with survival in ACC should be interpreted with caution, particularly in view of the relatively small size of the TCGA ACC cohort (77 samples).

In contrast, the upregulated genes in NCI-H295R ELAVL1-KO cells, as mentioned above, are mostly associated with tumor suppression. For example, *FUT3*, which is one of the strongest upregulated genes in our KO cells, on the one hand was described to promote metastasis in colorectal cancer [34] and is important for adhesion and binding capacities of gastric cancer cells [35], but on the other hand induces cytotoxicity mediated by natural killer cells in erythroleukemia and human melanoma cells [36,37]. *SPRR2A*, another of the top 10 upregulated genes, increases local tumor invasiveness in cholangiocarcinoma but also prevents metastasis [38].

Principal component analysis of the top eight upregulated genes did not reveal a clear separation between healthy adrenal tissue and ACC tumor samples in our dataset. In line with this, these genes exhibited heterogeneous expression patterns and, for most of them, only limited evidence is available regarding a potential role in tumor progression. Together, these observations are compatible with the notion that these genes may be regulated by ELAVL1, but that their contribution to ACC tumorigenesis has to be tested. Nevertheless, additional experimental and clinical studies will be required to clarify their functional relevance in the *ELAVL1*-dependent regulatory network in ACC.

*ELAVL1* also modulates the expression of key enzymes in steroidogenesis in NCI-H295R. In our model, *CYP21A2* and *CYP11B2* were upregulated, whereas *CYP17A1* and *CYP11B1* were downregulated at the transcript level as determined by RNA-seq, and these changes were paralleled by corresponding alterations in steroid profiles in cell culture supernatants, indicating that ELAVL1-dependent transcriptional deregulation is functionally reflected at the level of hormone production.

Expression of adrenal cytochrome P450 (CYP) enzymes is known to be regulated in a developmentally programmed manner [39], but can also respond dynamically to hormonal stimuli that activate the protein kinase A (PKA) pathway, such as ACTH [40,41]. Activation of the PKA pathway induces synthesis of the steroidogenic acute regulatory (StAR) protein, which directly promotes CYP expression and is considered the major regulatory factor in hormone-dependent steroid production [42]. More recently, additional mechanisms modulating adrenal CYP expression and steroid biosynthesis have been identified [43]; for example, sulfated steroids such as cholesterol sulfate can not only inhibit CYP enzyme activity by direct binding [44], but also suppress StAR expression in a human granulosa-like tumor cell line [45].

Given that multiple regulatory layers control adrenal steroid hormone synthesis, our findings raise the question of which of these mechanisms might be influenced, at least in part, by the mRNA stabilizing protein ELAVL1. Duan et al. identified AU-rich elements in the 3′ untranslated region of StAR mRNA but showed that StAR regulation is independent of *ELAVL1* expression [46], arguing against a direct ELAVL1–StAR axis. An alternative level at which ELAVL1 could affect CYP expression is through microRNA-mediated regulation, as several studies have demonstrated that distinct microRNAs directly modulate the expression of *CYP11B1*, *CYP11B2*, *CYP11A1*, and *CYP17A1* [47,48]. In addition, *CYP11B1* and *CYP11B2* expression levels are also controlled by epigenetic mechanisms, with gene methylation in particular being associated with reduced mRNA transcript abundance [49]. It would be of interest in future studies to compare the methylation status of the *CYP11B1* and *CYP11B2* loci between NCI-H295R *ELAVL1*-KO and control (Scr) cells, in order to explore whether *ELAVL1* perturbation may indirectly affect steroidogenic enzyme expression through epigenetic modulation.

Our findings suggest that loss of functional ELAVL1 may inhibit glucocorticoid and androgen synthesis while potentially enhancing mineralocorticoid production in NCI-H295R cells. Given that hypercortisolism, often accompanied by androgen excess, is one of the most frequent clinical manifestations in ACC and has been associated with poorer survival [9,10,50], aldosterone overproduction has mainly been described in isolated case reports [51,52]; it will be important to further dissect the molecular mechanisms by which *ELAVL1* influences steroidogenesis. Although *ELAVL1* knockout in NCI-H295R cells was associated with reduced glucocorticoid and androgen production and research into ELAVL1-directed inhibitors is ongoing, the therapeutic potential of targeting *ELAVL1* in ACC remains speculative at this stage and will require comprehensive preclinical and clinical evaluation.

## 4. Methods

### 4.1. In Silico Analysis of ELAVL1 Expression in ACC-Tissue

Using the online database GEPIA2 [53], we analyzed the ACC cohort (*n* = 77) of The Cancer Genome Atlas (TCGA) regarding the expression of *ELAVL1* compared to healthy adrenal tissue samples (*n* = 128). A log2 fold change cutoff was set to 1, and a *p*-value cutoff was set to 0.01. We also performed a survival analysis of the TCGA ACC cohort using GEPIA2. The cohort was divided into two groups: high *ELAVL1* expression and low *ELAVL1* expression. The median was chosen as the group cutoff. One sample is the median sample, which explains 38 samples for high and low expression. Afterwards, we compared expression-related survival across multiple cancer types. A heatmap showing the hazard ratio for the high *ELAVL1* expression group compared with the low *ELAVL1* expression group across different tumor entities was created. The significance level was set to 0.05.

### 4.2. Cell Culture

Adrenocortical carcinoma cell lines NCI-H295R and HAC15 were obtained from ATCC (CRL-128 and CRL-3301, respectively) and cultured in Dulbecco’s modified Eagle’s medium/F-12 Ham supplemented with 2.5% Nu-Serum I or 10% Cosmic Calf Serum, respectively, along with 1% ITS+ and 1% penicillin/streptomycin. Cells were maintained at 37 °C in a humidified atmosphere containing 5% CO_2_.

### 4.3. CRISPR/Cas9 Plasmid Design

The guide RNAs targeting the *ELAVL1* gene were designed using the CHOPCHOP [54] and ligated into the lentiCRISPRv2 vector (Addgene, Watertown, MA, USA) according to the protocol described in the Lentiv2 ligation kit [55]. Transformation of the vector was performed in DH5α *E. coli*, and plasmids were isolated using the PureLink™ HiPure Plasmid Filter Maxiprep Kit (Thermo Fisher Scientific; Waltham, MA, USA). The sequences are provided as Supplemental Table S1.

### 4.4. Virus Particle Production and Transduction

The CRISPR/Cas9 plasmid, together with lentiviral packaging plasmids (Thermo Fisher Scientific), was co-transfected into HEK293T cells using TurboFect transfection reagent (Thermo Fisher Scientific) according to the manufacturer’s protocol. Twenty-four hours after transfection, the culture medium was replaced, and the viral supernatant was collected 48 h later. NCI-H295R and HAC15 cells were then seeded in 12-well plates in 1 mL of culture medium, followed by the addition of 1 mL of virus-containing supernatant. After 48 h of incubation, the viral supernatant was removed, and transduced cells were selected with 3.5 µg/mL puromycin.

### 4.5. DNA Isolation, Sanger-Sequencing, and Mutation Analyses

To confirm mutations in the *ELAVL1* gene, DNA was isolated from cells using the GenElute™ Mammalian Genomic DNA Miniprep Kit (Sigma-Aldrich, St. Louis, MI, USA). DNA fragments flanking the target regions of the gRNAs were amplified by PCR using specific primers (Supplementary Table S1) and subsequently sequenced (Eurofins Genomics Luxembourg, Luxembourg). Mutation analyses were performed using the web tool “Tracking of Indels by DEcomposition” (TIDE) [56] to prove frame-shift mutations in the *ELAVL1* locus.

### 4.6. Clonal Isolation

To select KO single-cell clones, clonal isolation was performed by serial dilution in 96-well plates. Afterwards, total DNA was isolated, and PCR was performed to amplify the target region. The amplified DNA was sequenced using Sanger sequencing as described before. Using the TIDE web tool, only cells with frameshift mutations on both alleles were selected for follow-up experiments.

### 4.7. Immunofluorescence

Cells were seeded onto coverslips in 24-well plates and fixed with 4% formalin after removal of the culture medium. Cells were permeabilized with 0.1% Triton X-100 in PBS and blocked with 0.3% fish skin gelatin in PBS. The primary antibody against HuR/ELAVL1 (sc-5261; Santa Cruz Biotechnology, Dallas, TX, USA) was diluted in gelatin–PBS, applied to the cells, and incubated for 1 h at 37 °C. After three washes of 15 min each, the secondary antibody (Alexa Fluor 568 goat anti-mouse IgG; Thermo Fisher Scientific) was applied, together with DAPI for nuclear staining and Alexa Fluor 488 Phalloidin for cytoskeletal staining, and incubated for 1 h at 37 °C. Following three additional 15 min washes, coverslips were mounted on microscope slides using Fluoroshield histology mounting medium (Sigma-Aldrich, Steinheim, Germany). Images were acquired using a Keyence BZ-900 microscope (Keyence, Osaka, Japan) at 400× magnification.

### 4.8. Western Blot

Total protein was isolated by lysing cell pellets in Pierce RIPA lysis and extraction buffer (Thermo Fisher Scientific, Waltham, MA, USA) supplemented with protease inhibitor cocktail (40 μL/mL; Sigma-Aldrich). Protein lysates were mixed with 4× NuPAGE LDS Sample Buffer (Thermo Fisher Scientific) and incubated at 95 °C for 10 min. Proteins were separated by SDS-PAGE using 7.5% Mini-PROTEAN TGX Stain-Free Protein Gels (Bio-Rad, Hercules, CA, USA) and transferred onto 0.45 μm nitrocellulose membranes. Membranes were blocked with 5% skimmed milk in PBST and incubated with primary antibodies diluted in 5% skimmed milk for 1 h at room temperature. Membranes were subsequently incubated with peroxidase-conjugated secondary antibodies in PBST for 1 h at room temperature. Proteins were detected using the ROTI-Lumin detection system (Carl Roth, Karlsruhe, Germany) and imaged with a ChemiDoc Imager (Bio-Rad).

### 4.9. Soft Agar Colony Formation Assay

NCI-H295R scrambled control and ELAVL1-KO cells (10,000 cells/well) were seeded in 0.2% low-melting agarose (Biozym, Hessisch Oldendorf, Germany) atop a 0.5% agar layer in 48-well plates. After 14 days at 37 °C, images were captured at 25× magnification using an Axio Vert.A1 FL-LED microscope (Zeiss, Oberkochen, Germany). Colony number and size were quantified using ImageJ (Version 1.54h; threshold: 212; particle size: 70–200 pixels; exclude edge particles). Four independent experiments were performed with 12 wells per condition; damaged wells were excluded from analysis.

### 4.10. RNA Isolation and Next-Generation Sequencing (NGS)

For gene expression analysis, NCI-H295R and HAC15 cells (scrambled control and ELAVL1-KO) were seeded in 6-well plates and cultured to 60–80% confluence. Total RNA was isolated using the GenElute Mammalian Total RNA Miniprep Kit (Sigma-Aldrich, St. Louis, MI, USA) according to the manufacturer’s instructions. NGS was performed on a total of 15 independent samples: for NCI-H295R, 4 scrambled control and 6 *ELAVL1*-KO samples were analyzed; for HAC15, 2 scrambled control and 3 *ELAVL1*-KO samples were analyzed. All sequencing services were provided by Novogene Company Limited (Cambridge, UK). The reference genome used was Homo_sapiens_release_104. The read depth and quality control is summarized in Supplemental Table S3. Details on sequencing depth, mapping rates, alignment software, and expression quantification are available in the Supplementary Materials, where full report is provided.

### 4.11. cDNA Synthesis and Real-Time PCR

For real-time PCR, 1 µg of total RNA was reverse-transcribed into cDNA using the MMLV RT kit (Thermo Fisher Scientific). cDNA was diluted 1:40 prior to amplification. Primers were designed using NCBI Primer-BLAST (Primer3 version 2.5.0) to span at least one intron. Real-time PCR was performed on a CFX Connect Real-Time System (Bio-Rad). Data were analyzed using the 2^−ΔΔCt^ method [57], with GAPDH serving as the internal normalization control, and evaluated using Bio-Rad CFX Manager 3.1 software.

### 4.12. Measurement of Steroid Concentrations

To evaluate steroid secretion, 60,000 cells were seeded into 12-well plates and cultured in 1 mL of medium. The medium was replaced every 72 h for 10 days. Supernatants were collected, and hormone concentrations were measured as previously described [58]. Briefly, 13 steroid hormones—aldosterone, cortisone, corticosterone, progesterone, cortisol, 11-deoxycortisol, 17α-OH-progesterone, DHEAS, testosterone, androstenedione, DHEA, DHT, and estradiol—were quantified using the MassChrom Steroids assay (Chromsystems, Gräfelfing, Germany). A 500 µL aliquot of cell culture supernatant underwent offline solid-phase extraction in 96-well plates. Following sample cleanup and concentration, 15 µL of each sample was injected into an Agilent 1290 U-HPLC coupled to a QTRAP 6500+ tandem mass spectrometer (Sciex, Toronto, ON, Canada). Measurements were performed in ESI+ mode, except for aldosterone and DHEAS, which were analyzed in ESI− mode. Stable isotope-labeled standards were used for all analytes, and two multiple reaction monitoring (MRM) transitions (qualifier/quantifier) were established. Quantification was based on a 6-point calibration curve (1/x weighting) and processed using Analyst 1.6.3 software.

### 4.13. Statistics

Statistical analyses were performed using GraphPad Prism version 9.3.1 (GraphPad Software, San Diego, CA, USA). All data are presented as the mean ± standard deviation (SD) of at least three independent experiments. To assess normal distribution, we performed D’Agostino–Pearson and Shapiro–Wilk tests, using a significance threshold of *p* < 0.05. Statistical significance between two groups was determined using Student’s *t*-test, with the significance threshold set at *p* < 0.05.

## Figures and Tables

**Table 1:** Top 10 downregulated and upregulated genes in NCI-H295R ELAVL1-KO cells.

Top 10 Downregulated Genes	Log2 Fold Change	Top 10 Upregulated Genes	Log2 Fold Change
*AADAC*	−6.86	*MAB21L4*	5.05
*FAT3*	−6.86	*FUT3*	4.92
*SLPI*	−6.71	*AP1M2*	4.86
*CXCL6*	−6.63	*CHRNA2*	4.84
*LYPD1*	−6.27	*STRIT1*	4.76
*PLCXD3*	−6.26	*WBP2P1*	4.61
*PLA2G2A*	−6.03	*CHRNA4*	4.6
*ITGA4*	−5.95	*SPRR2A*	4.48
*PI3*	−5.68	*DSC1*	4.32
*HSD17B2*	−5.58	*SPRR1A*	4.29

**Table 2:** Top 10 downregulated and upregulated genes in HAC15 *ELAVL1*-KO-bulk population.

Top 10 Downregulated Genes	Log2 Fold Change	Top 10 Upregulated Genes	Log2 Fold Change
*SNX6P1*	−4.61	*GAGE2A*	6.84
*ZP3P2*	−4.21	*KLRC2*	6.10
*RUBCNL*	−3.81	*CYRIA*	6.02
*SNTG1*	−3.76	*KRT8*	5.81
*DAPK1*	−3.76	*SP3P*	5.45
*SRRM4*	−3.55	*OR51B4*	5.17
*LUZP2*	−3.13	*LEFTY2*	4.95
*CPNE4*	−3.04	*WBP2P1*	4.94
*FDPSP8*	−2.88	*S100A5*	4.84
*FOXA2*	−2.78	*FDX2*	4.83

**Table 3:** Deregulated genes in steroid synthesis in NCI-H295R ELAVL1-KO cells.

Upregulated	Log2 Fold Change	Downregulated	Log2 Fold Change
*CYP11B2*	3.77	*CYP17A1*	−1.43
*CYP21A2*	1.87	*CYP11B1*	−4.46

## Data Availability

Data are available from the corresponding author on request.

## Supplementary Materials

The following supporting information can be downloaded at: https://www.mdpi.com/article/10.3390/ijms27157001/s1. References [59,60,61,62,63,64,65,66,67,68] are cited in Supplementary Materials.
