# Supplementary material for: ELAVL1-KO Affects Steroid Synthesis in ACC Cell Line NCI-H295R and Reduces Colony-Forming Abilities
Source: Int J Mol Sci. 2026 Aug 4;27(15):7001. doi: 10.3390/ijms27157001 (PMC13467342; doi:10.3390/ijms27157001)
Supplement: Supplementary file 1 [file ijms-27-07001-s001.zip › NGS_Report.pdf]

1 Workflow of RNA sequencing projects

2 Analysis Pipeline

3 Appendix

RNA-seq Analysis Report

Contract.Information	Contract.Content
Contract ID	H204SC20092106
Batch ID	X204SC20092106-Z01-F014
Species and Version	Homo_sapiens_release_104
Project Name	DE-UniklinikumHalle-100-Mouse-mRNA9G-WBI
Report Time	2021-10-21

1 Workflow of RNA sequencing projects

A transcriptome is a set of all the transcripts in one cell or one population of cells at certain status. Transcriptome analysis assists to study the identification of genes that are differentially expressed in distinct cell populations. Researchers can also gain a deeper insight into gene boundary identification, variable cleavage and transcript variation. [1].

RNA sequencing via Illumina platforms, based on the mechanism of SBS (sequencing by synthesis), offers a wide range of benefits on high throughput and high accuracy out of low sample requirements. This technical method can be a powerful tool for researching RNA transcriptional activity.

RNA sequencing projects are carried out as follows:

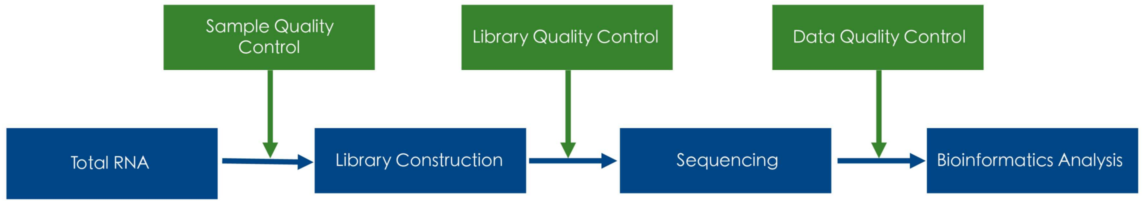

2 Analysis Pipeline

This is the workflow for mRNA sequencing data of standard bioinformatic analysis with a well-annotated reference genome, as follows:

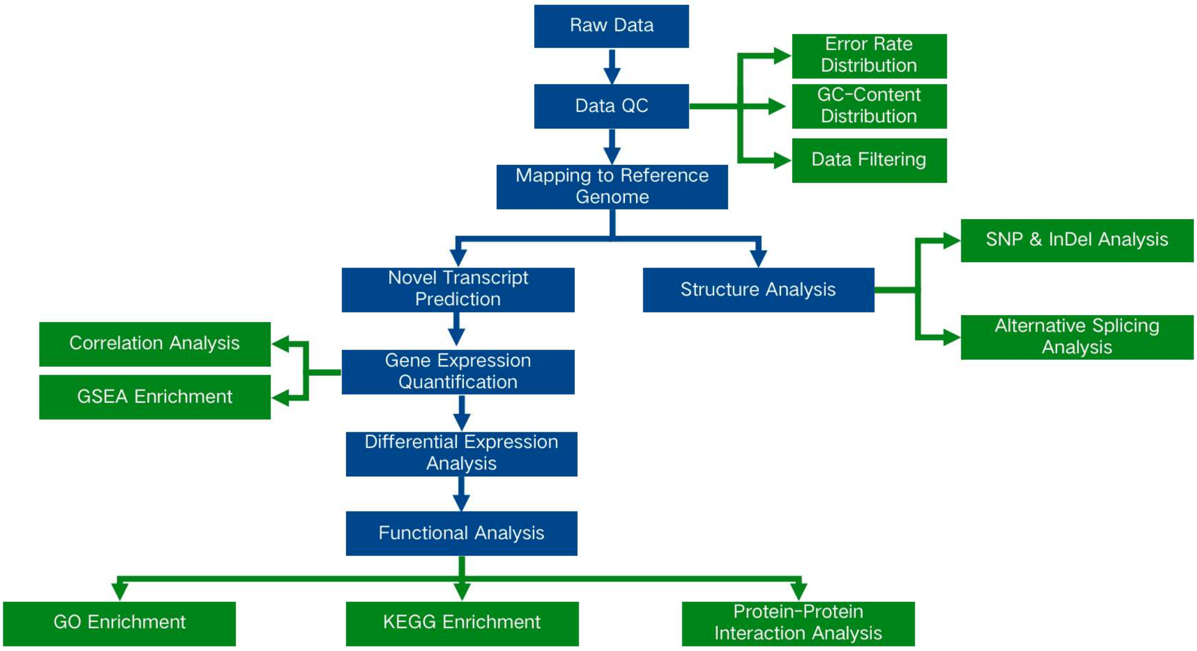

Figure 2.1 RNA-seq information analysis technology flow

For the analysis content of the above figure, if it exists in the content of the contract information analysis, the analysis is performed; if it does not exist, it is not performed.

2.1 Project Results

2.1.1 Raw data

Original image data file from high-throughput sequencing platforms (like Illumina) is transformed to sequenced reads (called Raw Data or Raw Reads) by CASAVA base recognition (Base Calling). Raw data are stored in FASTQ(fq) format files which contain sequences of reads and corresponding base quality. Each read has four descriptive lines, as indicated below:

Each read has four descriptive lines, as indicated below:

```
@A01426:11:H73WJDSX2:1:1101:2329:1016 1:N:0:GTGAGATC+GAACTAGC
TCGCACGCGCTGCCGTATGTGACGCCGTCGCTGCCGCACACAGGATCGTAGAGGCTCGC
+
FFFFFFFFFFFFFFFFFFFFFFFFFFFFFFFFFFFFFFFF,FFFFF:FFFFFFFFFFF
```

1. Line 1: Begins with the at sign (@) followed by sequence identifiers and optional description (such as FASTA header).
2. Line 2: Base sequences (raw read, A, G, C, and T).
3. Line 3: Begins with the plus sign (+) optionally followed by the same Illumina sequence identifiers and description information as in Line 1.
4. Line 4: The quality values for each base, corresponding to the data in Line 2.

Illumina Sequence Identifier:

Identifier	Meaning
**A01426**	Instrument – unique identifier of the sequencer
**11**	run number – Run number on instrument
**H73WJDSX2**	Flowcell ID - ID of flowcell
**1**	LaneNumber - positive integer
**1101**	TileNumber - positive integer
**2329**	X - x coordinate of the spot. Integer which can be negative
**1016**	Y - y coordinate of the spot. Integer which can be negative
**1**	Read Number - 1 can be single read or Read 2 of paired-end
**N**	Y if the read is filtered out (did not pass), N otherwise
**0**	control number - 0 when none of the control bits are on, otherwise it is an even number
**GTGAGATC+GAACTAGC**	Illumina index sequences

The details of Illumina Sequencing identifier are as follows:

1. A01426:11 A01426, Instrument - unique identifier of the sequencer; 11, Run number - Run number on instrument
2. H73WJDSX2:1:1101:2329:1016 means the coordinate of read on H73WJDSX2 (Flowcell ID) flowcell, line 1, 1101 tile is(x=2329, y=1016)
3. 1:N:0:GTGAGATC+GAACTAGC the first number is 1 or 2, 1 refers to single reads or the first read of paired ends, 2 refers to the second of paired ends; the second letter means whether reads is adjusted(Y means yes, N means no); the third number represent the number of Control Bits in sequence; six bases on the fourth place is Illumina index sequence.

2.1.2 Data Quality Control

The error rate for each base can be transformed by the Phred score as in equation 1. "e" represents sequencing error rate, "Qphred" represents base quality values of Illumina platforms (equation 1: Qphred = -10log10(e)).

Table 2.1 Illumina Casava 1.8 version base recognition and Phred score Concise correspondence between them

Phred.score	Base.Calling.error.rate	Base.Calling.correct.rate	Q.sorce
**10**	1/10	90%	Q10
**20**	1/100	99%	Q20
**30**	1/1000	99.9%	Q30
**40**	1/10000	99.99%	Q40

Sequencing error rate and base quality varies depending on sequencers, reagent residues, and different sample types. For RNA-seq technology, sequencing error rate distribution can be featured as below:

- Error rate increases with the sequencing reads for consumption of sequencing reagent. It is common in the Illumina high-throughput sequencing platform (Erlich Y, Mitra PP et al.2008; Jiang L, Schlesinger F et al.2011).
- The first six bases have a relatively high error rate due to the incomplete binding of random hexamers used in priming cDNA synthesis (Jiang et al.2011). In general, a single base error rate should be lower than 1%.

The sample sequencing error rate distribution is shown in the results file QC/1.Error (./result_tree.html). We also provide PDF and svg two types of image files.

h295_E1KO

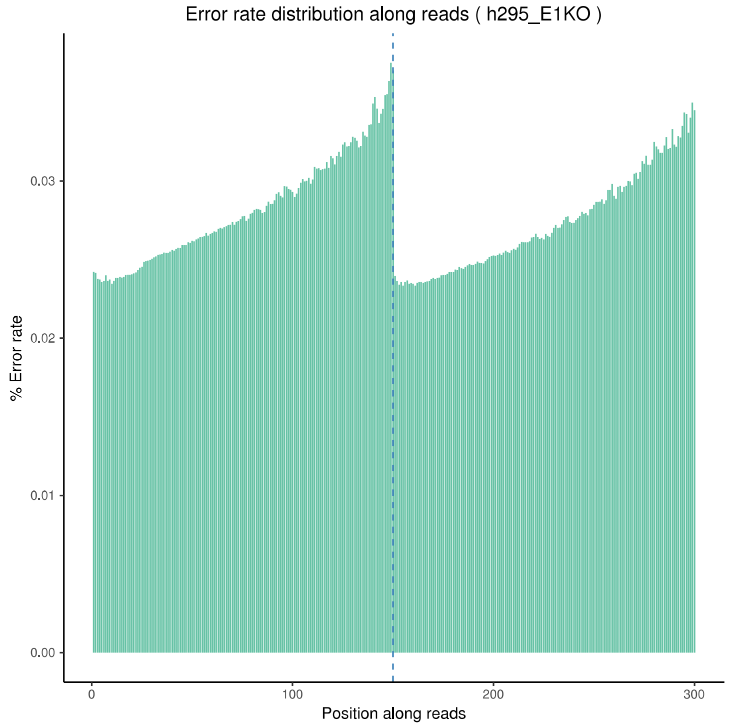

Figure 2.2 Sequencing data error rate distribution

The x-axis shows the base position along with each sequencing read and the y-axis shows the base error rate.

2.1.3 GC Content Distribution

GC content distribution indicates potential AT/GC separation which affects subsequent gene expression quantification. In view of random fragmentation and biological law of G/C-A/T content, G and C, A and T should be respectively equal, and the content should be stable throughout the entire sequencing process for the non-stranded library (If the library is strand-specific, AT separation or GC separation may occur). Large variations of sequencing error in the first 6-7 bases are allowed considering the use of random primer in library construction. It is normal that the first few bases have certain preferences in existing high-throughput sequencing technology. Result Directory: QC/2.GC (./result_tree.html)

h295_E1KO

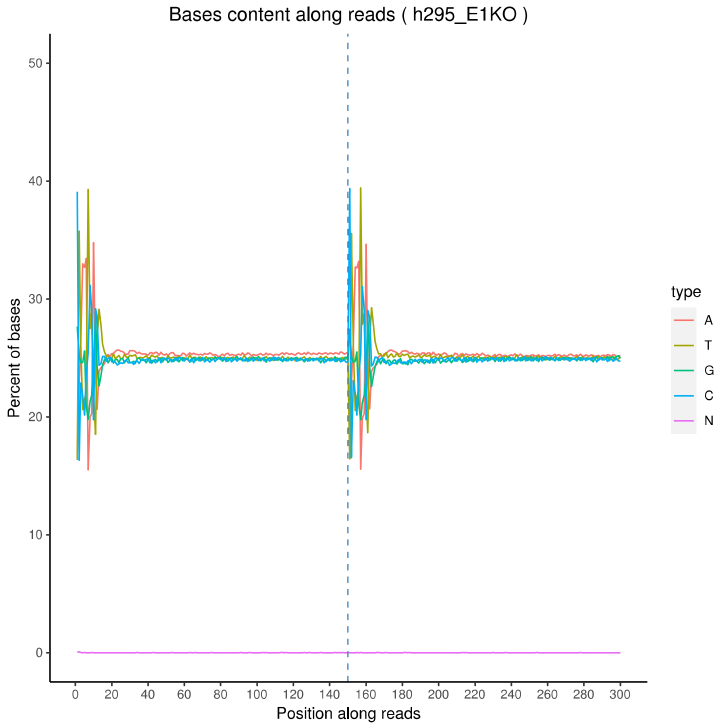

Figure 2.3 GC content distribution

The x-axis shows each base position within a read, and the y-axis shows the percentage of each base, with each base represented by a different color.

The left side of the vertical dashed line is the GC-content of read 1, the right side is the GC-content of read 2.

2.1.4 Data Filtering

The sequencing reads/raw reads often contain low-quality reads or reads with adapters, which will affect the quality of downstream analysis. To avoid this, it is necessary to filter the raw reads and obtain the clean reads.

Raw reads filtering is as follows:

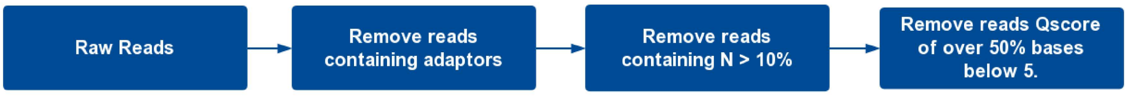

- Remove reads with adapter contamination.
- Remove reads when uncertain nucleotides constitute more than 10 per cent of either read (N > 10%).
- Remove reads when low-quality nucleotides (Base Quality less than 5) constitute more than 50 per cent of the read.

Adapter Sequences:

Adapter	Sequence
**P5 Adapter**	P5-AATGATACGGCGACCACCGAGA (5'-3')
	P5'-TTACTATGCCGCTGGTGGCTCT (3'-5')
**P7 Adapter**	CGTATGCCGTCTTCTGCTTG-P7' (5'-3')
	GCATACGGCAGAAGACGAAC-P7 (3'-5')

For the sequencing data of each sample, see the results file: QC/3.Filter (./result_tree.html), as shown below.

h295_E1KO

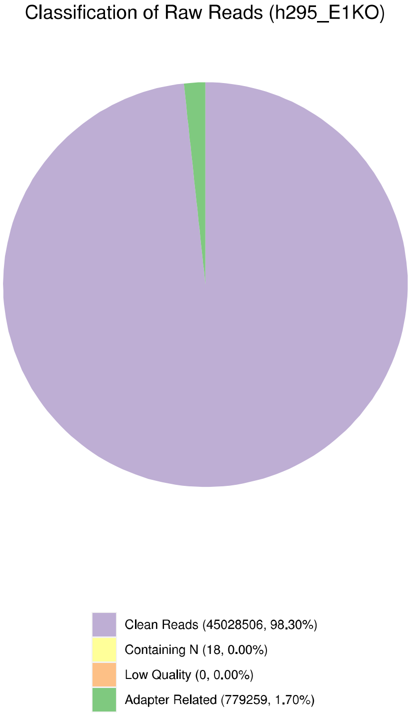

Figure 2.4 Sample Sequencing Data Filtering

Note: The proportions of the different colors in the graph represent the proportion of different components.

- **Adapter related**: (reads containing adapter) / (total raw reads).
- **Containing N**: (reads with more than 10% N) / (total raw reads).
- **Low quality**: (reads of low quality) / (total raw reads).
- **Clean reads**: (clean reads) / (total raw reads).

2.1.5 Data Quality Control Summary

Data are summarized in the table below.See the result file: QC/4.Stat/data_table.xls (./result_tree.html).

Table 2.2 Sample Sampling Data Quality Summary

sample	library	raw_reads	raw_bases	clean_reads	c
HAC_SCR1	ERRA210018680-1a	83887052	12.58G	82422734	
HAC_SCR2	ERRA210018681-1a	78093178	11.71G	76932056	
HAC_E3_1	ERRA210018682-1a	88338012	13.25G	87269924	
HAC_E3_2	ERRA210018683-1a	99334368	14.9G	98140034	
HAC_E3_3	ERRA210018684-1a	81877422	12.28G	80576604	

Showing 11 to 15 of 15 entries

Previous 1 2 Next

- **sample**: SampleID.
- **library**: library ID.
- **raw_reads**: Reads count from the raw data, four rows as a unit, with statistics of reads count for every sequencing.
- **raw_bases**: Base number of raw data. (number of raw reads) * (sequence length), converting unit to G.
- **clean_reads**: Base number of raw data after filtering. (number of clean reads) * (sequence length), converting unit to G.
- **clean_bases**: (clean base=clean reads*150bp) number multiply read length, saved in G unit.
- **error_rate**: Average sequencing error rate, which is calculated by Qphred=-10log10(e).
- **Q20**: The percentage of the bases whose Q Phred values is greater than 20. (Number of bases with Q Phred value > 20) / (Number of total bases) *100.
- **Q30**: The percentage of the bases whose Q Phred values is greater than 30. (Number of bases with Q Phred value > 30) / (Number of total bases) *100.
- **GC_pct**: The percentage of G&C base numbers of total bases.(G&C base number) / (Total base number)*100.

2.1.6 Data Quality Control Q&A

Interpretation of Relevant Nouns

- **Q20**:The percentage of the total bases have Phred greater than 20. Phred=-10log10(e)
- **Q30**:The percentage of the total bases have Phred greater than 30. Phred=-10log10(e)

The sequencing error rate increases as the sequence's length increases. What is the acceptable error rate?

Novogene set a high standard for sequencing quality. Generally, the error rate of a single base should be lower than 1%. In some special cases, the maximum error rate of a single base should not be greater than 6%.

2.2 Alignment

Perform alignments with HISAT2 (http://ccb.jhu.edu/software/hisat2/faq.shtml) to the reference. HISAT2[4] uses a graph-based alignment and has succeeded HISAT and TOPHAT2. HISAT2 is a fast and sensitive alignment program for mapping next-generation sequencing reads. Besides one global GFM index, HISAT2 also includes a large set of small GFM indexes that collectively cover the whole genome. These small indexes (local indexes), combined with multiple alignment strategies, enabled effective alignment of RNA-seq reads, particularly, reads spanning multiple exons. The following figure shows the algorithm of split reads comparison by HISAT2:

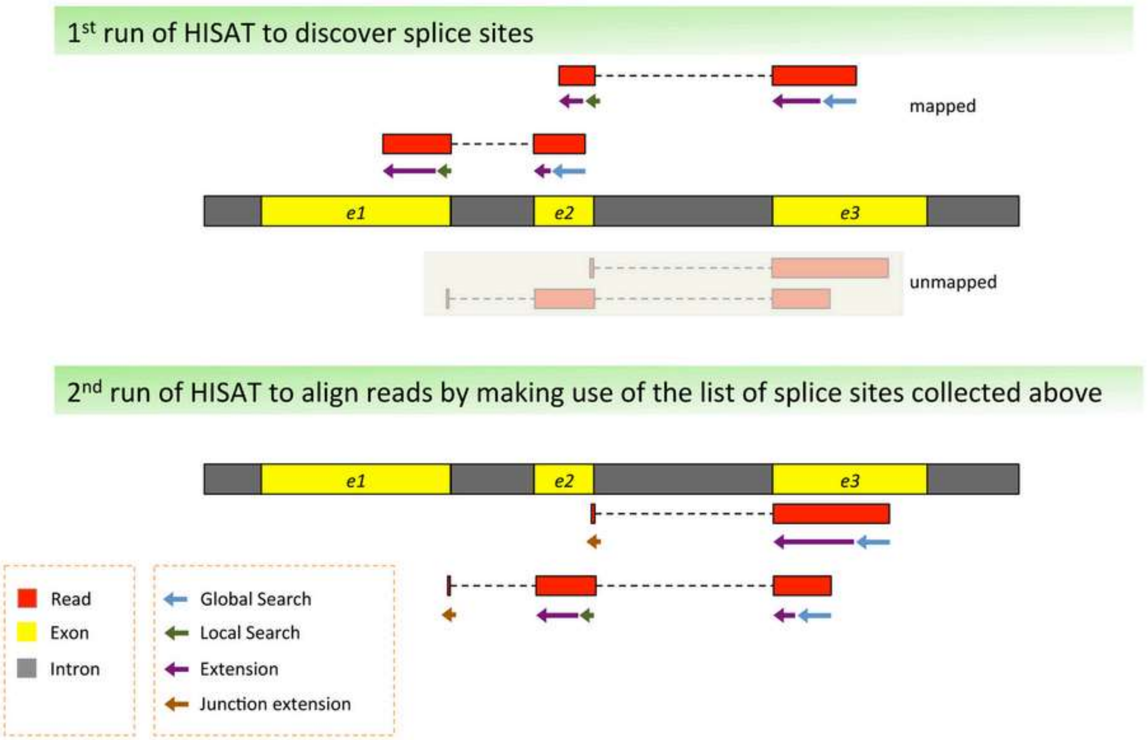

The algorithm of hisat is divided into three parts:

1. The whole sequence was aligned to a single exon.
2. The sequencing sequence was piecewise aligned to two exons of the genome.
3. The sequencing sequence was segmented and aligned to more than three (including three) exons of the genome.

2.2.1 Mapping Result

The data are summarized in the table below. The sample and reference genome comparisons can be found in the results file: Mapping/2.Stat/align_pct.xls (./result_tree.html).

Table 2.3 Comparison of sample and reference genomes

sample	total_reads	total_map	unique_map	multi_map

h295_SCR1	93568506	87931736(93.98%)	82547916(88.22%)	5383820(5.75%
h295_SCR2	105239296	98995853(94.07%)	92908087(88.28%)	6087766(5.78%
h295_SCR3	81366930	76573530(94.11%)	71859512(88.32%)	4714018(5.79%
h295_SCR4	81544432	77018170(94.45%)	72377808(88.76%)	4640362(5.69%
h295_E3_1	91942172	86742165(94.34%)	81396725(88.53%)	5345440(5.81%
h295_E3_2	94063908	88871818(94.48%)	83467589(88.73%)	5404229(5.75%
h295_E3_3	76585200	72255756(94.35%)	67851797(88.6%)	4403959(5.75%
h295_E3_4	83741324	78743648(94.03%)	73931161(88.29%)	4812487(5.75%
h295_E1KO	90057012	84599131(93.94%)	79315517(88.07%)	5283614(5.87%
h295_E2KO	108490704	102320424(94.31%)	96039007(88.52%)	6281417(5.79%

Showing 1 to 10 of 15 entries

Previous 1 2 Next

- **sample**: SampleID.
- **total_reads**: Total clean reads used for analysis.
- **total_map**: Number and percentage of reads aligned to the genome, the ratio should higher than 70%, total mapping rate: (mapped reads)/(total reads)*100.
- **unique_map**: Number and percentage of reads aligned to the unique position of the reference genome (for subsequent quantitative data analysis), unique mapping rate: (uniquely mapped reads)/(total reads)*100.
- **Multi_map**: number and percentage of reads aligned to multiple locations in the reference genome, multiple mapping rate: (multiple mapped reads)/(total reads)*100.
- **read1_map**: Number and percentage of read1 aligned to the reference genome.
- **read2_map**: Number and percentage of read2 aligned to the reference genome.
- **positive_map**: Number and percentage of reads aligned to the positive chain of the reference genome.
- **negative_map**: Number and percentage of reads aligned to the negative chain of the reference genome.
- **splice_map**: Number of spliced reads on the genome and its percentage.
- **unsplice_map**: Number of complete reads aligned to genome and its percentage.
- **proper_map**: Number of paired read1 and read2 aligned to the genome and its percentage.

2.2.2 Reads Distribution in Reference Genome

Mapped regions can be classified as exons, introns, or intergenic regions. Exon-mapped reads should be the most abundant type of reads when the reference genome is well-annotated. Intron-reads may be derived from pre-mRNA contamination or intron-retention from alternative

splicing. Reads mapped to intergenic regions are mainly attributed to weak annotations of the reference genome. The distribution of sequencing reads of all samples in the genomic region is shown in the figure below. See the results file: Mapping/1.Region (./result_tree.html).

h295_E1KO

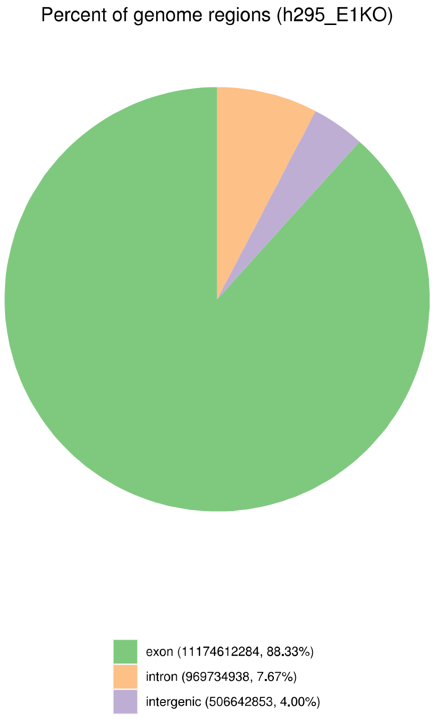

Figure 2.5 Sequencing reads in the genomic region

Note: The ratios of the different colors in the figure represent the ratio of reads to different regions

- **exon**: The number of reads aligned to exon regions of the genome and its proportion in clean reads.
- **Intron**: The number of reads aligned to intron regions of the genome and its proportion in clean reads.
- **Intergenic**: The number of reads aligned to intergenic regions of the genome and its proportion in clean reads.

2.2.3 Visualization of Mapping Results

Files provided in BAM format--a standard file format that contains mapping results–indicating the information of the corresponding referenced genome and gene annotations for some species. The Integrative Genomics Viewer (IGV) is a recommended software for visualizing data from BAM files.

IGV can be featured as below:

1. Displaying the positions of single or multiple reads in the reference genome, and read distribution between annotated exons, introns or intergenic regions, both in adjustable scale respectively;

2. Displaying the read abundance of different regions to demonstrate their expression levels, in adjustable scale;
3. Providing annotation information for both genes and splicing isoforms;
4. Providing other related annotation information;
5. Displaying annotations downloaded from remote servers and/or imported local machines.
6. IGV browser usage can refer to our provided documentation IGVQuickStart (src/IGVQuickStart.pdf)

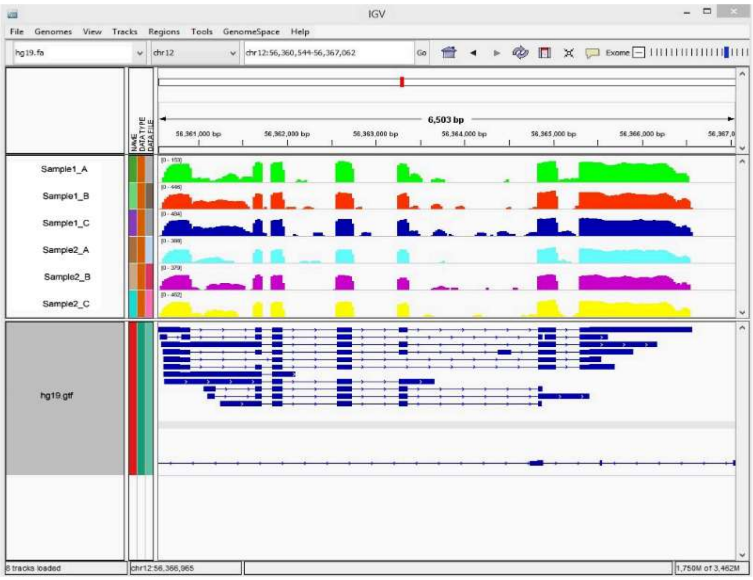

Figure 2.6 IGV Browser Comparison Visualization of Results

2.2.4 Alignment Q&A

What is the difference between the RNA data comparison software and the DNA data comparison software?

Due to the existence of alternative splicing during transcription, a large portion of the measured reads spans different exons, and all RNA data comparison software supports reads' splice comparison.

What are the reasons for the lower rate of mapping?

- The assembly of reference genome is not satisfying
- The relative relation between the tested species and the reference genome is far
- Special treatments or exogenous contaminations of samples

Does the mapping use full length of reads, or does the mapping use reads that are processed at the beginning and the end?

In our standard procedure, we use the standard RNA-seq kit whose indexes are in the middle of the adapters and the sequencing will be executed for the sequences with indexes. In this way, the sequences of read 1 and read 2 are from the samples and there is no need to process the sequences in mapping. Even if the adapters are in sequences or the quality is low, the read will be removed.

2.3 Novel Gene Prediction

2.3.1 Novel Gene Prediction

Mapping information from all samples is combined and placed as input into the regular Cufflinks assembler. The assembled transfrags are then compared to the reference transcripts to determine if they are sufficiently different to be considered novel. In brief, in this process we can (1) identify novel genes, (2) identify novel exons of known genes, and (3) optimize the start and end information of known transcripts[5]. The outputs are provided as GTF files; more information about GTF format is available at (http://mblab.wustl.edu/GTF22.html (http://mblab.wustl.edu/GTF22.html)). Result Directory: Assemble/*_novel.gtf (./result_tree.html).

Table 2.4 New Transcript Structure Comment Results

seqname	source	feature	start	end	score	strand
1	StringTie	exon	22324100	22324259	.	+
1	StringTie	exon	22328883	22329605	.	+
1	StringTie	transcript	44738060	44739282	.	+
1	StringTie	exon	44738060	44739282	.	+
1	StringTie	transcript	83198223	83242995	.	+

Showing 1 to 5 of 5 entries

Previous 1 Next

- **seqname**: Chromosome ID.
- **source**: The source of the annotation, stringTie here means that the transcript is assembled by stringTie software.
- **feature**: The structure type of the annotation information, such as gene, transcript, exon, etc.
- **start**: The starting coordinate of the annotation area on the chromosome.

- **end**: The end coordinate of the annotation area on the chromosome.
- **score**: The score of the annotation information which is a description of the possibility of annotation, and the dot is indicated as empty.
- **strand**: Annotation region positive and negative chain information on the chromosome.
- **frame**: Valid only for annotation types of CDS, valid values are 0/1/2.
- **attributes**: Contains a list of attributes, mainly gene number, transcript number, etc.

2.3.2 Novel Gene Annotation

After the novel gene is assembled, we will annotate the novel gene by using databases, such as Pfam, Swissprot, GO, KEGG. The Pfam, Swissprot annotation results are summarized in the table belowand. See result file: Assemble/*_novel_gene.xls (./result_tree.html).

Table 2.5 New Transcript Comment Results

gene_id	gene_name	gene_chr	gene_start	gene_end	gene_strar
novel.1	-	1	22324100	22329605	+
novel.2	-	1	44738060	44739282	+
novel.3	-	1	83198223	83242995	+
novel.4	-	1	143327566	143331413	+
novel.5	-	1	150629787	150635775	+

Showing 1 to 5 of 5 entries

Previous 1 Next

- **gene_id**: Gene’s ID.
- **gene_name**: The gene name.
- **gene_chr**: Chromosome/scaffold ID.
- **gene_start**: The starting coordinate of novel gene on the chromosome.
- **gene_end**: The end coordinate of novel gene on the chromosome.
- **gene_strand**: The positive and negative chain information of novel gene on the chromosome.
- **gene_length**: Novel gene length.
- **gene_biotype**: The type of novel gene.
- **gene_description**: Description from PFAM, Swissprot annotation.
- **gene_tf_family**: The tf_family annotation of novel gene.

2.3.3 Novel Gene Prediction Q&A

What is the significance of novel gene prediction?

For non-model species, the genetic annotation information is usually not perfect, and novel gene prediction can discover novel genes or transcripts of the species.

2.4 Gene Expression Level Analysis

Gene expression level analysis is the core task in the RNA-seq experiment. Gene expression level is calculated by the number of mapped reads.

2.4.1 Gene Expression Quantification

The abundance of transcripts reflects gene expression level directly. In RNA-seq experiments, gene expression level is estimated by the abundance of transcripts (count of sequencing) that mapped to genome or exon. Read counts is proportional to gene expression level, gene length and sequencing depth. FPKM (short for the expected number of Fragments Per Kilobase of transcript sequence per Millions base pairs sequenced) is the most common method of estimating gene expression levels, which takes the effects into consideration of both sequencing depth and gene length on counting of fragments (Mortazavi et al., 2008). The data are summarized in the table below. See result file: Quant/1.Count/gene_count.xls (./result_tree.html).

Table 2.6 Quantitative results of gene expression

gene_id	h295_SCR1	h295_SCR2	h295_SCR3	h295_SCR4
ENSG00000198804	427297	389861	450538	257413
ENSG00000198886	240873	211015	246788	168765
ENSG00000198712	225793	210571	233566	234792
ENSG00000198938	185085	192784	186270	137762
ENSG00000156508	182912	192043	142662	126679

Showing 1 to 5 of 5 entries

Previous 1 Next

- **gene_id**: Gene number.

- **sample**: Raw read count values of each sample.
- **gene_name**: Gene name.
- **gene_chr**: The name of the chromosome where the gene is located.
- **gene_start**: The starting position of the chromosome where the gene is located.
- **gene_end**: The end position of the chromosome where the gene is located.
- **gene_strand**: The positive and negative strand information of the chromosome where the gene is located.
- **gene_length**: Gene length, the sum of genes from the beginning to the end of all non-overlapping exon regions in chromosomes.
- **gene_biotype**: Gene type,such as coding protein genes,long non-coding genes,etc.
- **gene_description**: Gene description.
- **gene_tf_family**: Transcription factor family annotation information of a gene.

2.4.2 Distribution of Gene Expression Levels

To compare gene expression levels under different conditions, the distribution of gene expression levels and FPKM[8] among different samples are displayed by boxplots. For biological replicates, the final FPKM will be the mean value. Result Directory: Quant/3.Distribution/boxplot.svg (./result_tree.html).

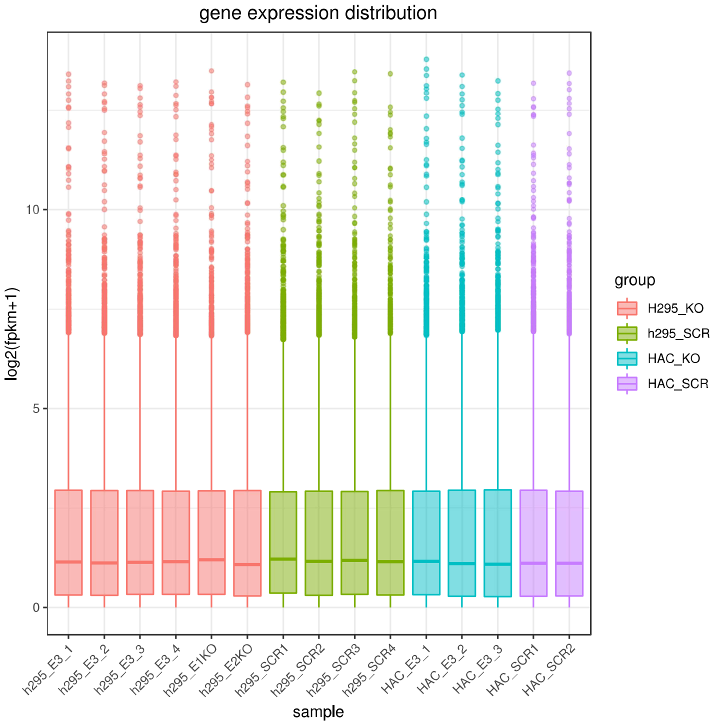

Figure 2.7 Sample gene expression distribution box plot

X axis represents the name of the sample. Y axis indicates the log2(FPKM+1). Parameters of box plots are indicated, including maximum, upper quartile, mid-value, lower quartile and minimum.

2.4.3 Correlation Analysis

Biological replicates are necessary for any biological experiment including RNA-seq technology. Correlation of the gene expression levels between samples plays an important role in verifying reliability and sample selection, which can not only demonstrate the repeatability of the experiment but estimate the differential gene expression analysis.

The closer the correlation coefficient is to 1, the higher similarity the samples have. Encode suggests that the square of the Pearson correlation coefficient should be greater than 0.92 (under ideal experiment conditions) and the R^2 should be greater than 0.8.

According to all gene expression levels (RPKM or FPKM) of each sample, the correlation coefficient of samples between groups is calculated and drawn as heat maps. It is intuitive to show sample differences and repeat cases between groups. The higher the correlation coefficient of the sample is, the closer the expression pattern is. The correlation coefficient matrix is shown in the following figure. Result Directory: Quant/2.Correlation/correlation.svg (./result_tree.html).

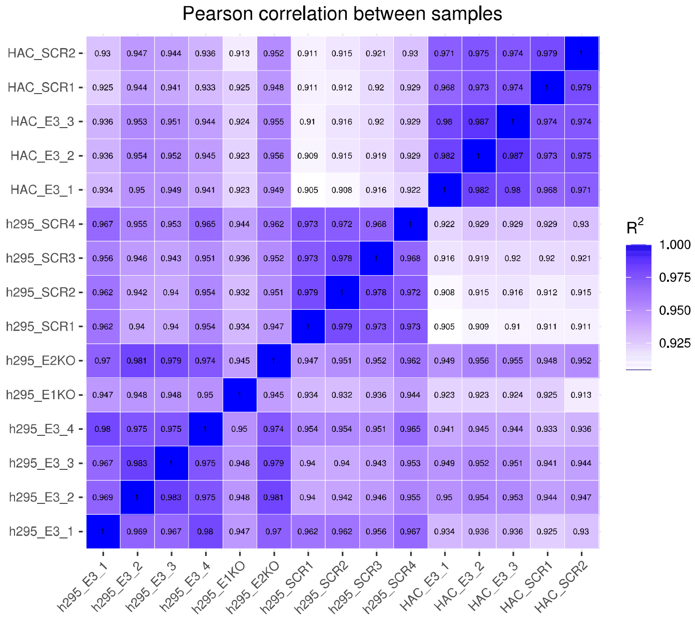

Figure 2.8 Inter-sample correlation heat map

R^2: Square of Pearson correlation coefficient(R)

2.4.4 Principal Component Analysis

Principal component analysis (PCA) is also commonly used to evaluate intergroup differences and intragroup sample duplication. PCA uses the linear algebra calculation method to reduce dimension and extract principal components from tens of thousands of gene variables. We performed PCA analysis on the gene expression value (FPKM) of all samples, as shown in the

figure below. Under ideal conditions, the samples between groups should be dispersed and the samples within groups should be gathered together. See the result file: Quant/2.Correlation/pca.svg (./result_tree.html).

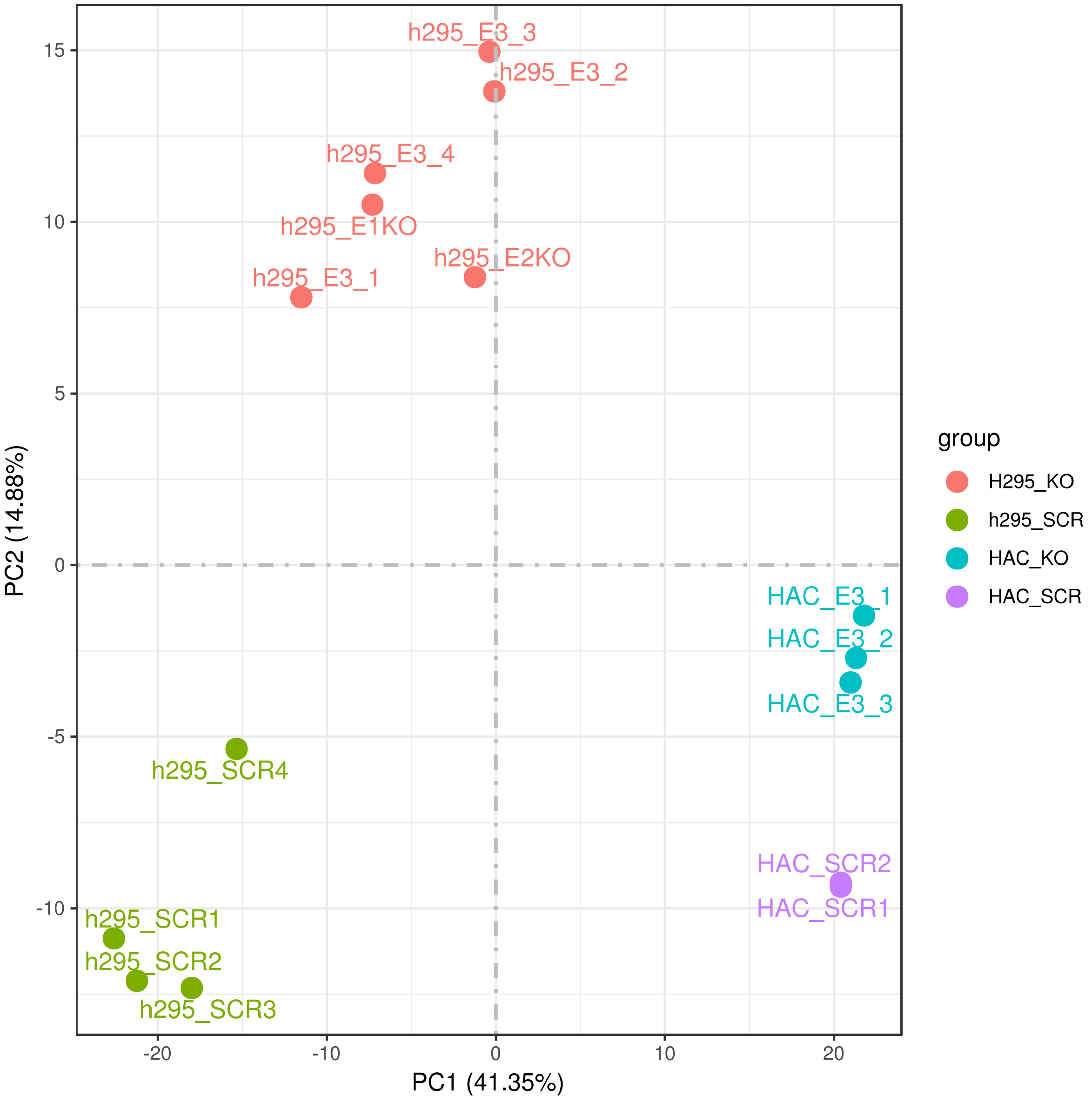

Figure 2.9 Principal component analysis result

2.4.5 Coexpression Venn Diagram

The coexpression Venn diagram presents the number of genes that are uniquely expressed within each group/sample, with the overlapping regions showing the number of genes that are co-expressed in two or more groups/samples. Result Directory: Quant/4.coExpression_venn (./result_tree.html)

venn1

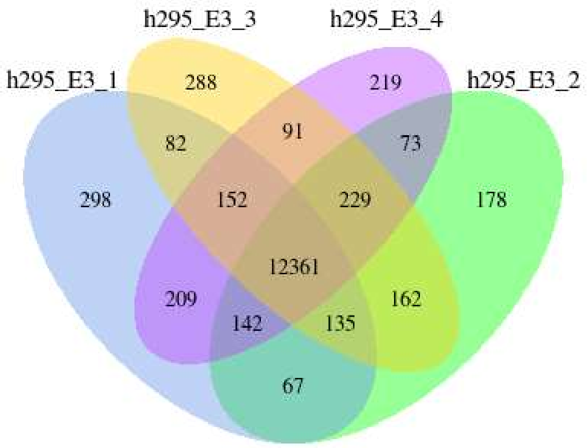

2.4.6 Quantification Analysis Q&A

How do you deal with reads (reads multi mapped) mapped to multiple locations in the genome during the quantification analysis?

Reads mapped to multiple locations in the genome are not possible to be determined their corresponding genes. Therefore, these reads are directly filtered out during the quantification analysis.

For two genes with overlapping regions, how are the reads in the overlapping region assigned during the quantification analysis?

Same as the previous answer.

How is the information of tf_family annotation of a gene obtained in the quantification analysis table?

On the one hand, it is annotated by the tf database (AnimalTFDB/PlantTFDB) (for the species already included in the database). On the other hand, it is predicted by protein domain databases such as Pfam/SUPERFAMILY.

How is FPKM calculated?

The calculation of FPKM(expected number of Fragments Per Kilobase of transcript sequence per Millions base pairs sequenced) takes into account the influence of sequencing depth and gene length on the count of fragments, and it is a commonly used method for estimating gene expression levels (Trapnell, Cole, et al., 2010).

What is the threshold for gene expression levels? Why is this threshold set?

Gene expression is generally considered to be greater than 1 for FPKM, a threshold recommended by mainstream journals.

What is the significance of the correlation between samples? How is it calculated?

The sample correlation represents the similarity among samples. The sample correlation could help us examine the similarity at the gene expression level. Higher correlation means higher similarity and less number of differential express genes. Generally, the correlation between biological replicates should be higher than the correlation of samples with different sources. There are three different calculation methods: A. Pearson correlation; B. Spearman rank correlation; C. Kendall's τ. Novogene uses R language to calculate the Pearson correlation coefficient.

What is the pca?

Principal Component Analysis (PCA) is a multivariate dimensionality reduction analysis method. The key of PCA is to reduce the dimension of the data under the premise of keeping the data as much as possible. Briefly, This method will ignore the less relevant variables to describe the relations between different samples. A dataset is a group of points in a multidimensional space and PCA method will move all the points to a new coordinate system without changing their relative spatial position, making their projections have the largest variances in the new coordinate. In this new coordinate system, the axis with the largest variance of projection is PC1 and the second largest one is PC2.

2.5 Differential Gene Expression Analysis

After the gene expression is quantified, statistical analysis of the expression data is required to screen the genes whose expression levels are significantly different in different conditions. The differential analysis is mainly divided into three steps.

- First, the raw readcount is normalized, mainly to correct the sequencing depth;
- Next, the statistical model is used to calculate the hypothesis test's probability (pvalue);
- Finally, multiple hypothesis test corrections are used to obtain FDR values (false discovery rate).

For different experimental conditions, we selected appropriate software for gene expression differential analysis, as shown in the following table.

Table 2.7 Software for Differential Analysis and Differential Gene Screening Criteria

Type	Software	Normalized Method	pvalue Calculation Model	FDR Calculation Method	Differential Gene Screening Threshold
Biological Replicates	DESeq2(Anders et al., 2014)	DESeq	The Negative Binomial Distribution	BH	|log2(FoldChange)| >= 1 & padj <= 0.05
No Biological Replicates	edgeR(Robinson et al., 2010)	TMM	The Negative Binomial Distribution	BH	|log2(FoldChange)| >= 1 & padj <= 0.05

If the number of different genes screened according to the above threshold is too small (less than 100), there will likely be no significant results in the subsequent functional enrichment analysis. Therefore, we will appropriately reduce the threshold for screening different genes according to the specific conditions of the project. If the project experiment only focuses on the expression of a few genes (such as gene knockout), please ignore the enrichment results, and filter the genes of interest from the differential analysis table below.

In general, if a gene differs more than twice as much in expression in both sets of samples, we believe that such genes are differentially expressed. In order to judge whether the difference in expression between two samples is due to various errors or essential differences, we need to make a hypothesis test on the expression data of all genes in these two samples. The transcriptome analysis is performed on thousands of genes, which leads to the accumulation of false positives. The more the number of genes, the higher the cumulative degree of false positives in the hypothesis test, so the introduction of padj to the hypothesis test P-value is calibrated to control the proportion of false positives [13].

The screening criteria for differential genes are very important. The standard we give |log2(FoldChange)| >= 1 & padj<= 0.05 is a commonly used empirical value in actual projects. It can be flexibly selected according to the situation. For example, the difference multiple can be 1.5 times, or 3 times, padj include 0.01, 0.05, 0.1, etc. If the number of differential genes screened according to the above criteria is too small, it is likely that there will be no significant results in the subsequent functional enrichment analysis. If the project experiment only focuses on the expression of a few genes (such as gene knockout), please ignore the enrichment results, and filter the genes of interest from the differential analysis table below. Conversely, if the number of differential genes obtained is too large, which is not conducive to the screening of subsequent target genes, screening can be performed using stricter threshold criteria at this time, and screening can be performed using stricter threshold criteria.

2.5.1 Result of Differential Expression Analysis

The differential significance analysis for each compare group is shown in the table below. The table shows the first 30 rows of the differential significance results for all the first compare group. See the results file: Differential/1.deglist (./result_tree.html).

Table 2.8 Differential Gene List Partial Results Presentation

gene_id	h295_E3_1	h295_E3_2	h295_E3_3	
ENSG00000185274	133.955782042521	134.057726062439	112.628310157621	122
ENSG00000066044	1570.3137661335	1618.06737890748	1639.4966385831	155
ENSG00000040731	22.4889269122481	12.1870660056762	16.2556323938835	15.[illegible]
ENSG00000183049	166.222503264443	161.244257921255	157.911857540582	14
ENSG00000187398	23.4667063432154	30.9363983221012	13.9333991947572	20.[illegible]

Showing 1 to 5 of 5 entries   Previous 1 Next

- **gene_id**: Gene id.
- **sample**: The readcount values of each sample after normalization.
- **group**: The standardized average of readcount for each group of samples.
- **log2FoldChange**: The ratio of gene expression level between the treatment group and the control group was processed by the shrinkage model of the differential analysis software, and finally the logarithm was taken with 2 as the base.
- **pvalue**: Pvalue in hypergenometric tests.
- **padj**: The corrected pvalue of multiple hypothesis test.
- **gene_name**: Gene name.
- **gene_chr**: The name of the chromosome where the gene is located.
- **gene_start**: The starting position of the chromosome where the gene is located.
- **gene_end**: The end position of the chromosome where the gene is located.
- **gene_strand**: The positive and negative strand information of the chromosome where the gene is located.
- **gene_length**: Gene length, the sum of genes from the beginning to the end of all non-overlapping exon regions in chromosomes.
- **gene_biotype**: Gene type,such as coding protein genes,long non-coding genes,etc.
- **gene_description**: Gene description.
- **gene_tf_family**: Transcription factor family annotation information of gene.

The significance analysis result of each gene in all compare groups was shown in the following table. See the results file: Differential/1.deglist/all_compare.xls (./result_tree.html).

Table 2.9 The results of each gene's differential significance analysis in all comparison combinations are shown

gene_id	h295_SCR1_count	h295_SCR2_count	h295_SCR3_count

ENSG00000198804	427297	389861	45053
ENSG00000198886	240873	211015	24678
ENSG00000198712	225793	210571	23356
ENSG00000198938	185085	192784	18627
ENSG00000156508	182912	192043	14266

Showing 1 to 5 of 5 entries

Previous 1 Next

- **gene_id**: Gene number.
- **sample_count**: Raw readcount value of each sample.
- **sample_fpkm**: FPKM value of each sample.
- **compare_treat**: The average readcount value of treatment group in compare group after normalized.
- **compare_control**: The average readcount value of control group in compare group after normalized.
- **compare_log2FoldChange**: The ratio of gene expression level between the treatment group and the control group of a comparison combination was processed by the shrinkage model of the differential analysis software, and finally the logarithm was taken with 2 as the base.
- **compare_pvalue**: Pvalue in hypergenometric test of compare group.
- **compare_padj**: The corrected pvalue of multiple hypothesis test of compare group.
- **gene_name**: gene name.
- **gene_chr**: The name of the chromosome where the gene is located.
- **gene_start**: The starting position of the chromosome where the gene is located.
- **gene_end**: The end position of the chromosome where the gene is located.
- **gene_strand**: The positive and negative strand information of the chromosome where the gene is located.
- **gene_length**: Gene length,the sum of genes from the beginning to the end of all non-overlapping exon regions in chromosome.
- **gene_biotype**: Gene type,such as coding protein genes,long non-coding genes,etc.
- **gene_description**: Gene description.
- **gene_tf_family**: Transcription factor family annotation information of gene.

2.5.2 Differential Gene Statistics

The statistics of the number of differential genes (including up-regulation and down-regulation) for each compare group and the threshold for screening are shown in the table below, see the results file: Differential/1.deglist/diff_stat.xls (./result_tree.html).

Table 2.10 Differential Gene Statistics Results

compare	all	up	down	thr
h295_E3KOvsh295_SCR	7583	3626	3957	DESeq2 pvalue<=0.05 |log2FoldCha
h295_E1KOvsh295_SCR	6250	2949	3301	DESeq2 pvalue<=0.05 |log2FoldCha
h295_E2KOvsh295_SCR	4220	1773	2447	DESeq2 pvalue<=0.05 |log2FoldCha
h295_E1KOvsh295_E3KO	6009	2885	3124	DESeq2 pvalue<=0.05 |log2FoldCha
h295_E2KOvsh295_E3KO	1016	384	632	DESeq2 pvalue<=0.05 |log2FoldCha
h295_E2KOvsh295_E1KO	3812	1344	2468	edgeR pvalue<=0.05 |log2FoldChar
H295_KOvsh295_SCR	6926	3468	3458	DESeq2 pvalue<=0.05 |log2FoldCha
HAC_KOvsHAC_SCR	4304	2261	2043	DESeq2 pvalue<=0.05 |log2FoldCha
h295_SCRvsHAC_SCR	9118	4939	4179	DESeq2 pvalue<=0.05 |log2FoldCha
HAC_KOvsH295_KO	8156	3670	4486	DESeq2 pvalue<=0.05 |log2FoldCha

Showing 1 to 10 of 10 entries

Previous 1 Next

- **compare**: Compare group name.
- **all**: The total number of differential genes in the compare group.
- **up**:The up-regulation number of differential genes in the compare group.
- **down**: The down-regulation number of differential genes in the compare group.
- **threshold**: The compare group software and thresholds for differential gene screening.

The number of differential genes (including up-regulation and down-regulation) for each comparison combination is shown in a histogram:

diff_stat

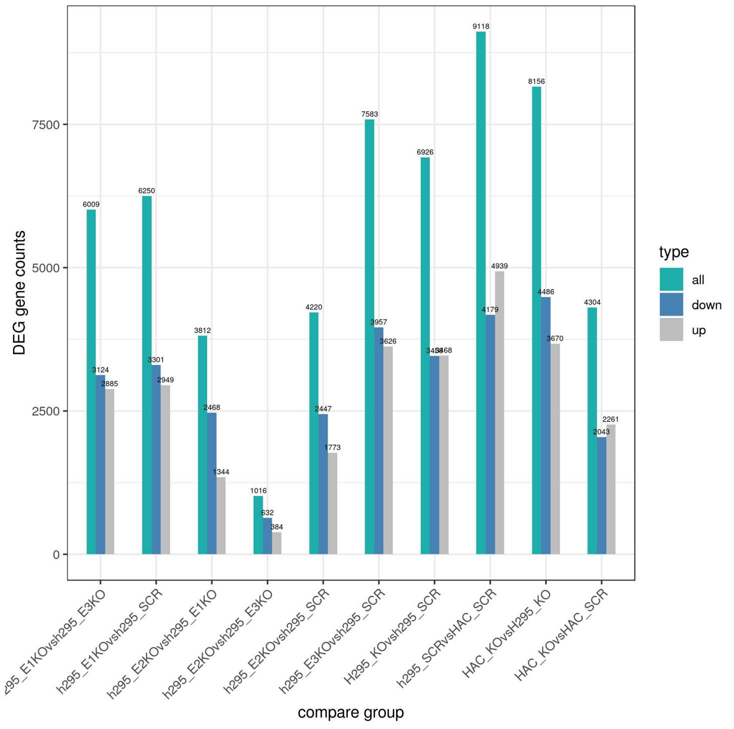

Figure 2.10 Difference Comparison Combine Differential Gene Number Statistics Histogram

Note: Blue and gray represent the differential genes for up-regulation and down-regulation, respectively, and the numbers on the columns indicate the number of differential genes

Volcano plots can be used to infer the overall distribution of differentially expressed genes. In the figure, The x-axis shows the fold change in gene expression between different samples, and the y-axis shows the statistical significance of the differences. Red dots represent up-regulation genes and green dots represent down-regulation genes. See the result file: Differential/1.deglist/{comparative combination}/_volcano.png (./result_tree.html).

h295_E1KOvsh295_E3KO

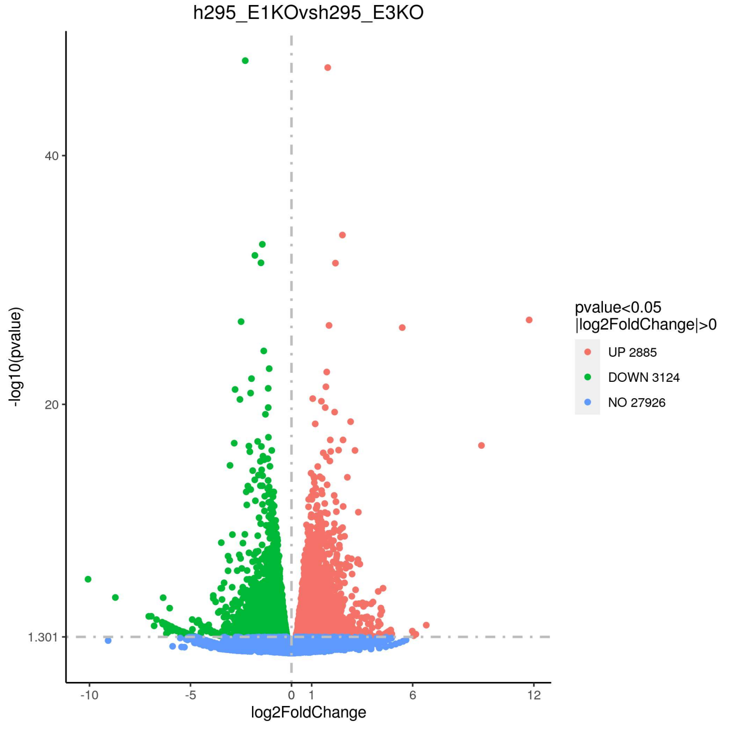

Figure 2.11 Differential Gene Volcano Map

The abscissa in the figure is log2FoldChange, and the ordinate is -log10padj or -log10pvalue, the blue dashed line indicates the threshold line for differential gene screening criteria

2.5.3 Venn Diagram

The Differential Expression Venn diagram illustrates the differential genes of the compared treatment group and the control group. The sum of all the numbers in the circle represents the total number in the compared groups, and the overlapping area indicates the number of differential genes shared between the groups, as shown in the following figure. See the result file: Differential/2.venn (./result_tree.html).

venn1

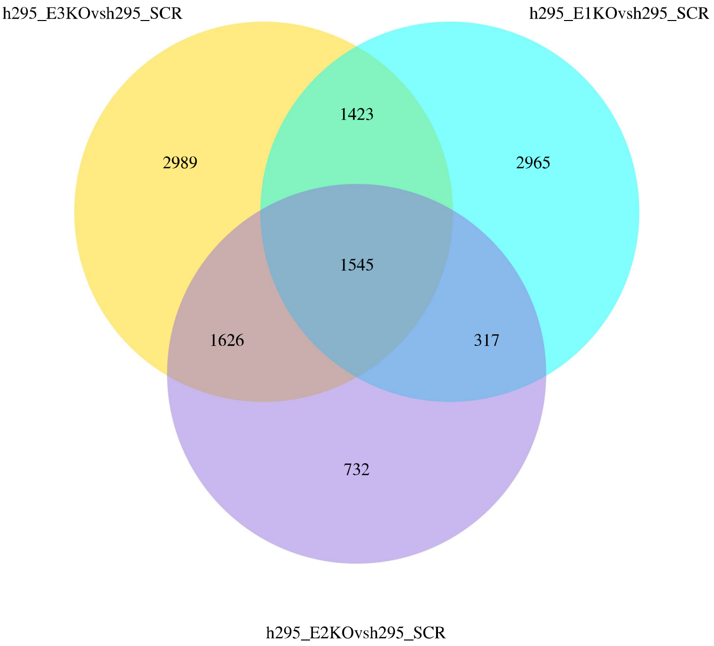

2.5.4 Cluster Analysis

All the differentially expressed genes in the comparison group were pooled as the differential gene set. For more than two groups of experiments, cluster analysis can be carried out on different gene sets and genes with similar expression patterns can be clustered together. We used the mainstream hierarchical clustering to cluster the fpkm values of genes, and homogenized the row (Z-score). The genes or samples with similar expression patterns in the heat map will be gathered together. The color in each grid reflects not the gene expression value, but the value obtained after homogenizing the expression data rows (generally between - 2 and 2). Therefore, the colors in the heat map can only be compared horizontally (the expression of the same gene in

different samples), but not vertically (the same sample). There are not only inter group clustering, but also inter sample clustering. The final report shows the clustering among samples. See the result file: Differential/3.cluster/heatmap.png (./result_tree.html).

heatmap

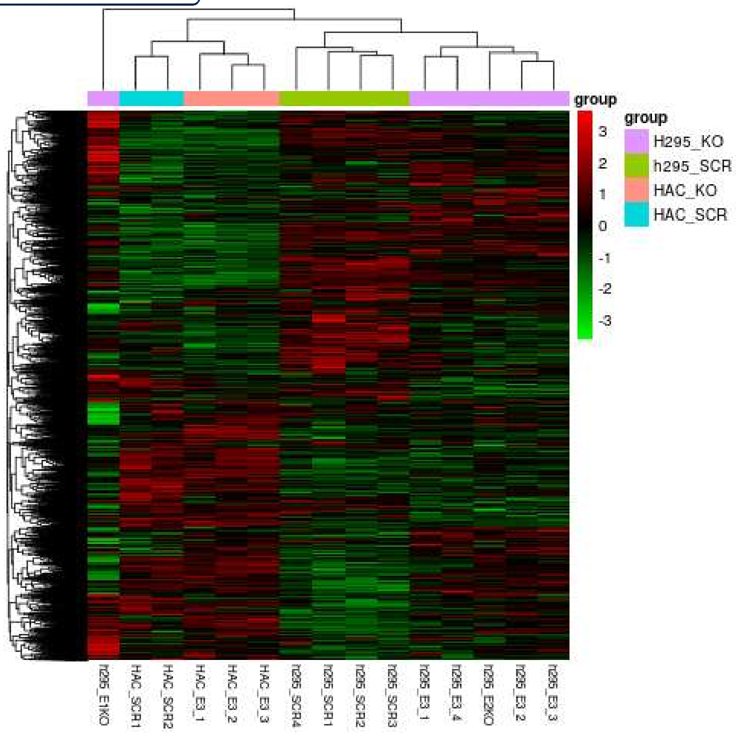

Figure 2.12 differential expression gene clustering heatmap

The overall results of FPKM cluster analysis, clustered using the log2(FPKM+1) value. Red color indicates genes with high expression levels, and blue color indicates genes with low expression levels. The color ranging from red to blue indicates that log2(FPKM+1) values where from large to small.

2.5.5 Differential Gene Expression Analysis Q&A

Can I use FPKM for differential analysis?

Most of the differential analysis software (DESeq, DESeq2 and edgeR) use the raw read counts as the input file and the negative binomial distribution model to estimate the probability of gene differential expression between samples. The software itself does some corrections on the read count (mainly the depth of sequencing), while FPKM is the corrected expression value, so it is unreasonable to use FPKM for the differential analysis to make double corrections.

Why do multiple hypothesis tests calculate padj values instead of using pvalues directly to screen fordifferential genes?

There is no problem with the usage of pvalue for a single hypothesis test, but in the process of differential analysis, we have to perform a hypothesis test on each gene. There are often tens of thousands of genes in a species, and tens of thousands of hypothesis tests are performed, which leads to greatly increasing false positives. Assuming a pvalue of 0.05 (only five of the one hundred differential genes are false positives), this accuracy is sufficient for a gene that is hypothesized, but for the entire tens of thousands of genes, such accuracy is far from good. For example, for every 10,000 genes tested, around 500 genes are false positive. In order to properly control false positive rates, it is necessary to introduce a stricter indicator which is the corrected pvalue. Of course, if there are too few differential genes, you can also use pvalues instead. As long as it has biological significance and can be verified by experiments.

What is the maximum threshold that can be set for differential gene screening? Is it appropriate to lower the threshold properly?

In general, the presetting screening threshold in higher-level article is more stringent, and in some articles, the differential gene screening thresholds are appropriately lower. As in some articles without biological replicates, only padj is used as a differential gene screening threshold, regardless of log2foldchange. While some other articles use pvalue as a screening threshold for differential genes.

What are the steps in cluster analysis?

Logarithmic transformation of gene expression values (FPKM expression matrices) of all samples is performed to approximate the data to an ideal normal distribution (clustering analysis rely on an early assumption that the data is subject to an idealized statistical distribution, usually Normal Distribution), then the software calculates the distance (Euclidean distance) of all the transformed data points, and finally classifies N objects into k groups by the complete clustering method in hierarchical clustering. The objects in each group are mutually similar. The hierarchical clustering algorithm is mainly divided into three steps, as shown below.

- At the initial moment, all points are themselves a cluster.
- Find the nearest two clusters to form a cluster. The distance between the two clusters refers to the distance between the two nearest points in the cluster.
- Repeat the second step until all points are clustered into individual cluster.

How is normalization calculated in cluster analysis?

The values of each row of data in the expression matrix are subtracted from the mean of each row of data, and divided by the standard deviation of each row of data. The range of values after normalizationare generally in the interval [-2,2].

How is the transcription factor predicted?

Identification of animal transcription factors uses the animal transcription factor database(animalTFDB 3.0), and identification of plant transcription factors uses the plant transcription factor database(PlantTFDB PlantTFDB 4.0). For the species included in the database, if Ensembl geneid is used, the transcription factor is directly screened; the Ensembl geneid is subjected to SUPERFAMILY and Pfam annotation by interproscan software, and the ID of SUPERFAMILY and Pfam of each gene are obtained. Then, the annotations information of the transcription factor family corresponding to each SUPERFAMILY and Pfam in the DBD (Transcription factor prediction database) are used to predict transcription factors. The fungal transcription factor is predicted by the SUPERFAMILY and Pfam annotations by interproscan software, and the ID of SUPERFAMILY and Pfam of each gene are obtained, and then each of the already annotated databases in the DBD(Transcription factor prediction database) are utilized. The information of the transcription factor family corresponding to SUPERFAMILY and Pfam is predicted.

2.6 Functional Analysis

Through the enrichment analysis of the differential expressed genes, we can find out which biological functions or pathways are significantly associated with differentially expressed genes. Novogene uses the clusterProfiler (Yu G, 2012) software for enrichment analysis, including GO Enrichment, DO Enrichment, KEGG and Reactome database Enrichment etc,.

$$P = 1 - \sum_{i=0}^{m-1} \frac{\binom{M}{i}\binom{N-M}{n-i}}{\binom{N}{n}}$$

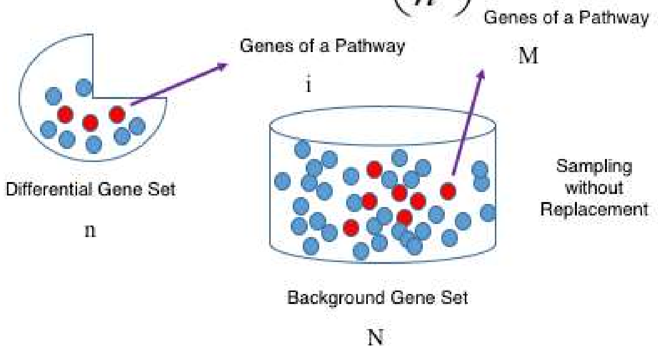

Figure 2.13 Gene Enrichment Analysis Schematic

2.6.1 GO Enrichment Analysis

GO is the abbreviation of Gene Ontology (http://www.geneontology.org/ (http://www.geneontology.org/)), which is a major bioinformatics classification system to unify the presentation of gene properties across all species. It includes three main branches: cellular component, molecular function and biological process. GO terms with padj < 0.05 are significant enrichment. See the result file: Enrichment/GO (./result_tree.html).

Table 2.11 Differential Gene GO Enrichment Analysis Results Partially Displayed

Category	GOID	Description
BP	GO:0007155	cell adhesion
BP	GO:0022610	biological adhesion
BP	GO:0098609	cell-cell adhesion
BP	GO:0007156	homophilic cell adhesion via plasma membrane adhesion molec
BP	GO:0098742	cell-cell adhesion via plasma-membrane adhesion molecules

Showing 1 to 5 of 5 entries

Previous 1 Next

- **Category**: Classification of GO databases, including biological processes(BP), cellular components(CC), molecular function(MF).
- **GOID**: Unique identification id of Gene Ontology database.
- **Description**: Function description corresponding to the GO number.
- **GeneRatio**: Ratio between the number of differentially expressed genes in each GO term and all differentially expressed genes that can be found in GO database.
- **BgRatio**: In background GO database, the ratio of all genes concerning this GO term to all genes.
- **pvalue**: Statistics category term; abbreviation for probability value.
- **padj**: Adjusted p-value. Generally, GO Terms with Corrected_pValue < 0.05 are significant enrichment.
- **geneID**: Differentially expressed genes in this term.
- **geneName**: Differentially expressed genes in this term.
- **Count**: The number of difference gene annotated to GO number.
- **Up**: Number of up expressed genes concerning this GO term.
- **Up_Gene_id**: Up expressed gene id concerning this GO term.
- **Down**: Number of down expressed genes concerning this GO term.
- **Down_Gene_id**: down expressed gene id concerning this GO term.

In the results of the GO enrichment analysis, the most significant 30 Terms were selected for display. If the results are less than 30, all terms would be drawn, as shown in the following figure. In this figure, the abscissa is GO Term. The ordinate is the significance level of GO Term enrichment. Higher values correspond to higher significance. The different colors represent the three GO subclasses of BP，CC，MF. See the result file: Enrichment/GO (./result_tree.html).

h295_E1KOvsh295_E3KO.al

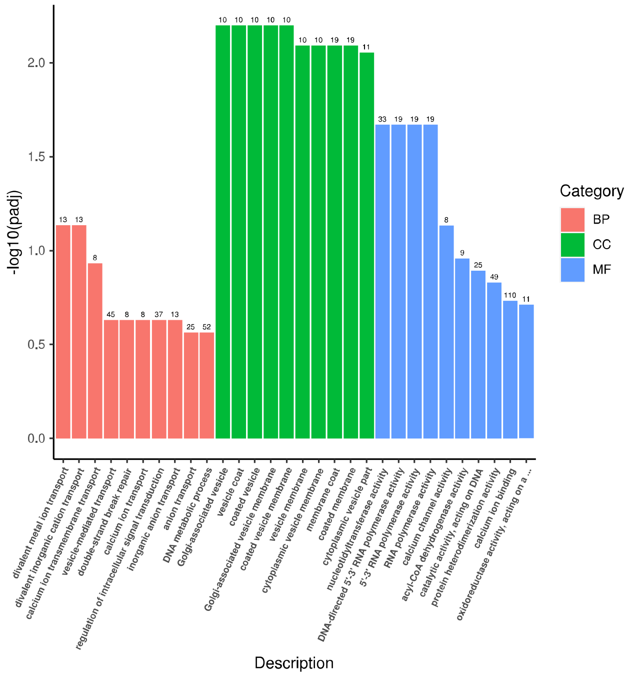

Figure 2.14 GO enrichment analysis histogram

Note: The abscissa in the figure is GO Term, and the ordinate is GO Term's level of significance of enrichment, expressed as -log10(padj). Different colors represent different functional categories

From the GO enrichment analysis result, the most significant 30 Terms were selected for display. If the results are less than 30, all Terms would be drawn according to major categories of biological processes, cell components, molecular functions and categories of up and down expressed genes.

h295_E1KOvsh295_E3KO.al

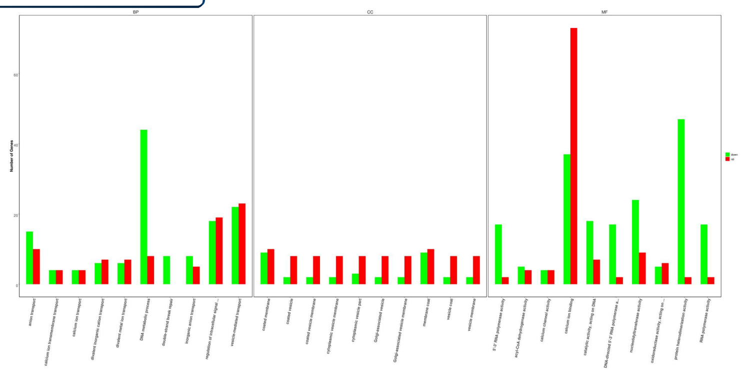

The abscissa in the figure is GO Term, and the ordinate is GO Term The level of significance of the set

From the GO enrichment analysis results, the most significant 30 GO Terms were selected for display. If the results are less than 30, all Terms would be drawn. In the figure, the abscissa is the ratio of the number of differential genes linked with the GO Term to the total number of differential

genes, and the ordinate is GO Term. The size of a point represents the number of genes annotated to a specific GO Term, and the color from red to purple represents the significant level of the enrichment. See the result file: Enrichment/GO (./result_tree.html).

h295_E1KOvsh295_E3KO.al

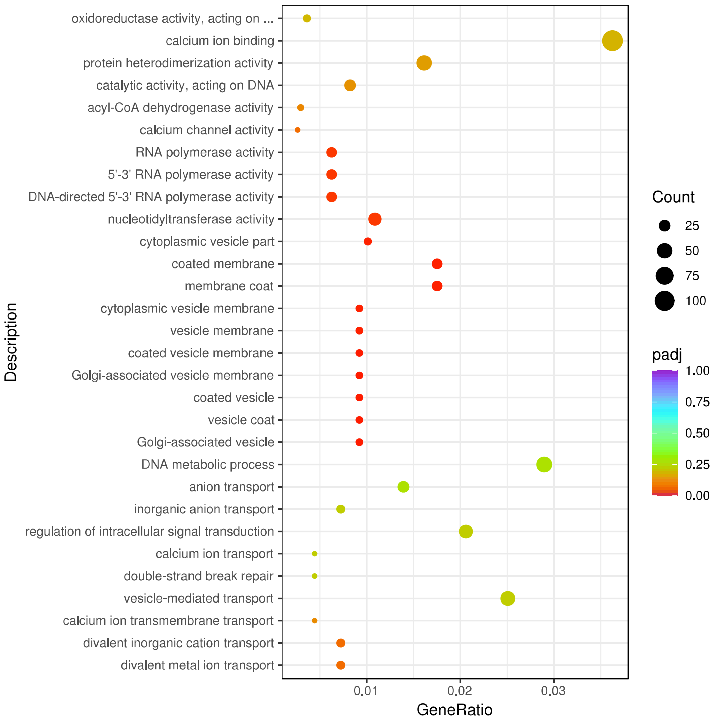

Figure 2.15 GO Enrichment Analysis Scatter Plot

The abscissa in the graph is the ratio of the differential gene number to the total number of differential genes on the GO Term, and the ordinate is GO Term

(Directed Acyclic Graph, DAG) could visualize the enriched GO Term of differential expression genes and its hierarchy. In this graph, branch means the hierarchical relation, and the function ranges become increasingly specific from the top to bottom. In general, the top 5 results of GO enrichment analysis are chosen as the main nodes (shown by box) in directed acyclic graph, and related GO Term are shown together by hierarchical connections. The enrichment degree is illustrated by color shades, the darker the shades are, the higher the enrichment degree is. In this project, DAG of biological process, molecular function and cellular component were drawn sequentially. See the result file: Enrichment/GO (./result_tree.html).

h295_E1KOvsh295_E3KO.al

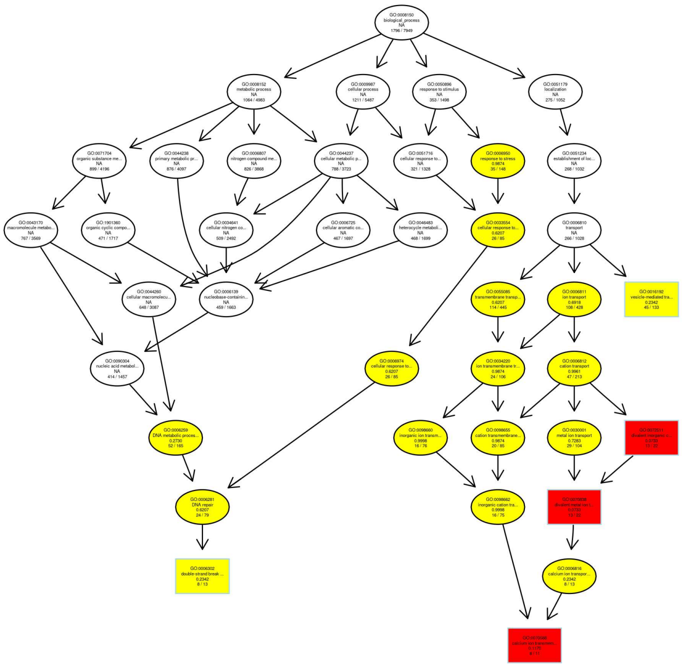

Figure 2.16 GO Enrichment Analysis DAG Chart

Each node represents a GO term, and the box represents the enrichment level of TOP5 GOTerms. The depth of the color represents the degree of enrichment, the darker the color is, the higherthe enrichment degree is. Each node shows the name of the term and the padj of enrichment analysis

2.6.2 KEGG Enrichment Analysis

The interactions of multiple genes may be involved in certain biological functions. KEGG (Kyoto Encyclopedia of Genes and Genomes) is a collection of manually curated databases containing resources on genomic, biological-pathway and disease information (Kanehisa,2008). Pathway enrichment analysis identifies significantly enriched metabolic pathways or signal transduction pathways associated with differentially expressed genes, comparing the whole genome background. KEGG pathways with padj < 0.05 are significant enrichment. See the result file: Enrichment/KEGG (./result_tree.html).

Table 2.12 KEGG Enrichment Analysis Partial Results

KEGGID	Description	GeneRatio	BgRatio		p

hsa04512	ECM-receptor interaction	43/2017	81/6270	0.0000710697744
hsa04360	Axon guidance	75/2017	161/6270	0.000078455436
hsa04015	Rap1 signaling pathway	77/2017	168/6270	0.00012335687
hsa04151	PI3K-Akt signaling pathway	125/2017	297/6270	0.000150135122
hsa04510	Focal adhesion	81/2017	184/6270	0.000421521368

Showing 1 to 5 of 5 entries

Previous 1 Next

- **KEGGID**: Unique identification id of KEGG database.
- **Description**: Function description of this pathway.
- **GeneRatio**: Ratio between the number of differentially expressed genes in each pathway and all differentially expressed genes that can be found in KEGG database.
- **BgRatio**: In background KEGG database, the ratio of all genes concerning this KEGG pathway to all genes included.
- **pvalue**: Statistics category term; abbreviation for probability value.
- **padj**: Adjusted p-value. Generally, KEGG pathways with Corrected_pValue < 0.05 are significant enrichment.
- **geneID**: Differentially expressed genes in this pathway.
- **geneName**: Differentially expressed genes in this pathway.
- **keggID**: Pathway gene id.
- **Count**: Number of differentially expressed genes concerning this pathway.
- **Up**: Number of up expressed genes concerning this KEGG pathway.
- **Up_Gene_id**: Up expressed gene id concerning this KEGG pathway.
- **Down**: Number of down expressed genes concerning this KEGG pathway.
- **Down_Gene_id**: Down expressed gene id concerning this KEGG pathway.

In the KEGG enrichment results, the most significant 20 KEGG pathways were selected for display. If the results are less than 20, all pathways would be drawn, as shown in the following figure. In this figure, the abscissa is the KEGG pathway, and the ordinate is the significance level of the pathway enrichment. Higher values correspond to higher significance. See the result file: Enrichment/KEGG (./result_tree.html).

h295_E1KOvsh295_E3KO.al

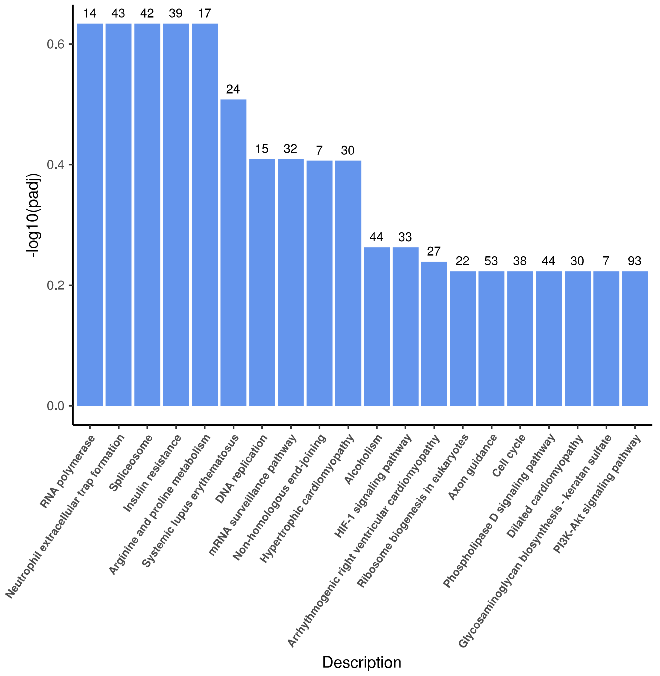

Figure 2.17 KEGG enrichment analysis histogram

The abscissa is the KEGG pathway, and the ordinate is the significance level of pathway enrichment

From the KEGG enrichment results, the most significant 20 KEGG pathways were selected for display. If the results are less than 20, all pathways would be drawn. In this figure, the abscissa is the ratio of the number of differential genes linked with the KEGG pathway to the total number of differential genes. The ordinate is KEGG Pathway. The size of a point represents the number of genes annotated to a specific KEGG pathway. The color from red to purple represents the significant level of the enrichment. See the results file: Enrichment/KEGG (./result_tree.html).

h295_E1KOvsh295_E3KO.al

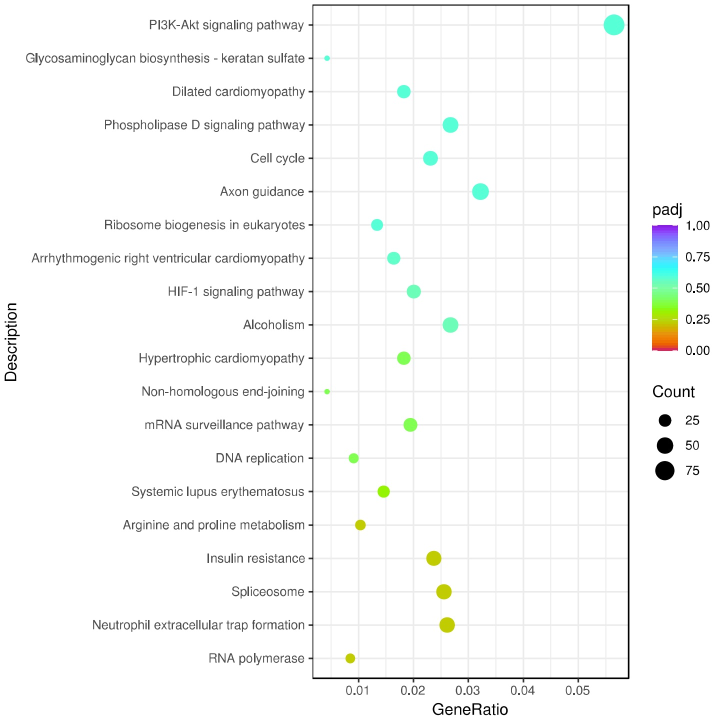

Figure 2.18 KEGG enrichment scatter plot

The abscissa in the graph is the ratio of the number of differential genes on the KEGG pathway to the total number of differential genes, and the ordinate is KEGG pathway

The html file can interactively help you view the significantly enriched KEGG pathway map by clicking corresponding links. In the map, the KEGG node including up-regulated genes is marked red, and the KEGG node including down-regulated genes is marked green. While yellow means the node contains both types of genes. Hovering over the marked KEGG node will show details of differentially expressed genes, with the same color as above, and the number inside brackets is log2(Foldchange). The above steps can be implemented offline. If you are connected to the Internet, click on each node to get the specific information of each KEGG node in the KEGG database. See the result file: Enrichment/KEGG (./result_tree.html).

h295_E3KOvsh295_SCR.all_

The most enriched pathway terms

Statistic method: hypergeometric test

FDR correction method: Benjamini and Hochberg

Term	Description	Sample number	Background number	P-va

2.6.3 Protein-Protein Interaction Network Analysis

The protein-protein interaction network is constructed for differential expression gene by searching STRING protein interaction database STRING (http://string-db.org/), the result file can be found in Enrichment/PPI (./result_tree.html).

Protein-protein interaction is provided as a network file that can be imported into Cytoscape software for visualization and edition. The central organizing metaphor of Cytoscape is a network graph, with molecular species represent as nodes and intermolecular interactions represent as edges.

1. Customized network data display using powerful visual styles.
2. View superposition of gene expression ratios and p-values on the network. Expression data can be mapped to node color, label, border thickness, or border color, etc. according to user-configured colors and visualization schemes.
3. Layout networks in two dimensions. A variety of layout algorithms are available, including cyclic and spring-embedded layouts.
4. Zoom in/out and pan for browsing the network.
5. Use the network manager to easily organize multiple networks. And this structure can be saved in a session file.
6. Use the bird's eye view to easily navigate large networks.
7. Easily navigate large networks (100,000+ nodes and edges) by an efficient rendering engine.

2.6.4 Enrichment Analysis Q&A

What is the meaning of n, N, i, M in the enrichment analysis schematic?

In the enrichment analysis results, the column of GeneRatio represents the differential gene set information. The number to the left of the slash represents the number of differential genes annotated to a pathway that corresponds to i of the enrichment schematic. The number to the right of the slash represents the differential gene annotated to all pathways that corresponds to n of the enrichment analysis schematic. The column of BgRatio represents the background gene set information. The number to the left of the slash is the number of background genes annotated to the pathway, corresponding to the M of the enrichment analysis schematic, and to the right of the slash is annotated background genes in all pathways, corresponding to the N of the enrichment analysis schematic.

Does the enrichment analysis result only look at the channels of significant enrichment?

The interpretation of the results of enrichment analysis should be based on biological significance. pvalue and padj only provide a reference. As long as the results can be explained and have biological significance, those pathways that are not significantly enriched are worthy of further study.

2.7 Alternative Splicing Analysis

Alternative Splicing (AS) is a universal gene regulation process in most eukaryotes. Eukaryotic split genes consist of intronic and exonic sequences. During RNA processing, the introns are spliced by spliceosomes while the exons are continuously edited and retained in the mature mRNAs. Some pre-mRNAs may have different splicing patterns in different conditions and yield different protein isoforms, which increases biological complexity and adaptability of eukaryotes.

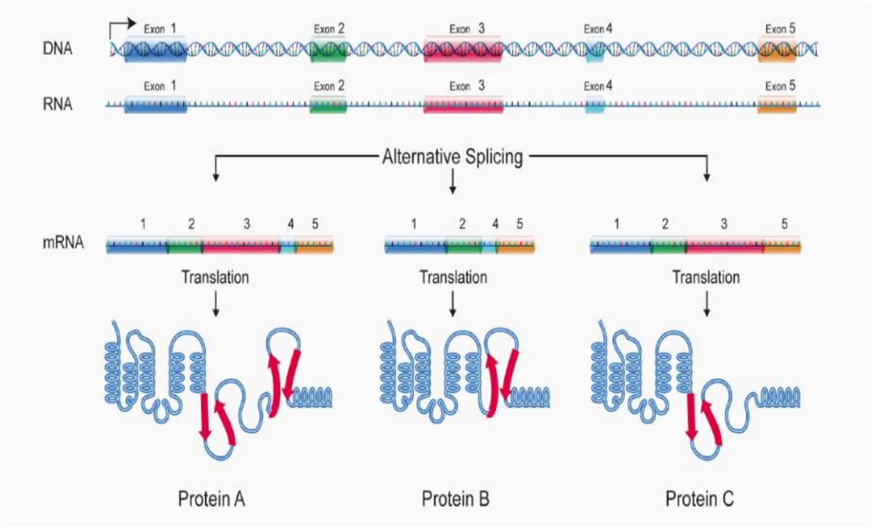

rMATS (http://rnaseq-mats.sourceforge.net/index.html) is a suitable RNA-Seq data alternative splicing analysis software [16], which not only can classify variable splicing events, but also perform differential analysis of alternative splicing events across different samples. Alternative splicing events as shown below:

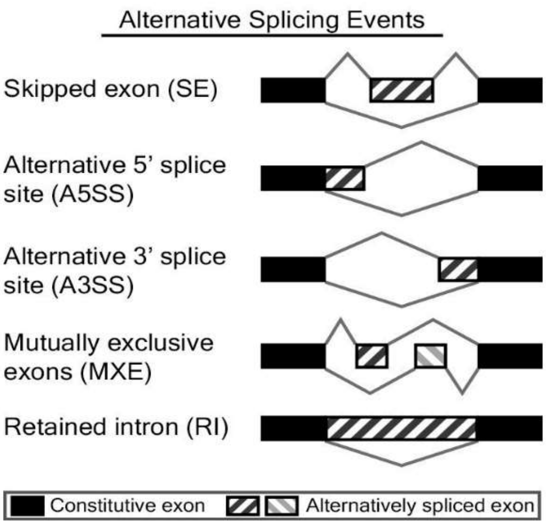

Figure 2.19 Variable splicing event classification

- **Skipped exon**: Skipped exon
- **MXE**: Mutually exclusive exon
- **A5SS**: Alternative 5' splice site
- **A3SS**: Alternative 3' splice site
- **RI**: Retained intron

2.7.1 Detection of Differentially Expressed Isoforms

Every alternative splicing event corresponds to 2 isoforms, which are referred to as Exon Inclusion Isoform and Exon Skipping Isoform. Statistics for expression quantity is conducted by dividing the effective length of expression when calculating the ratio of Exon Inclusion Isoform and the 2 Isoform whole expression quantity and differential significant analysis. The threshold of differential AS analysis is set as FDR < 0.05. The AS events in the below table are evaluated only with reads spanning splicing junctions. See the results file: AS/1.daslist (./result_tree.html).

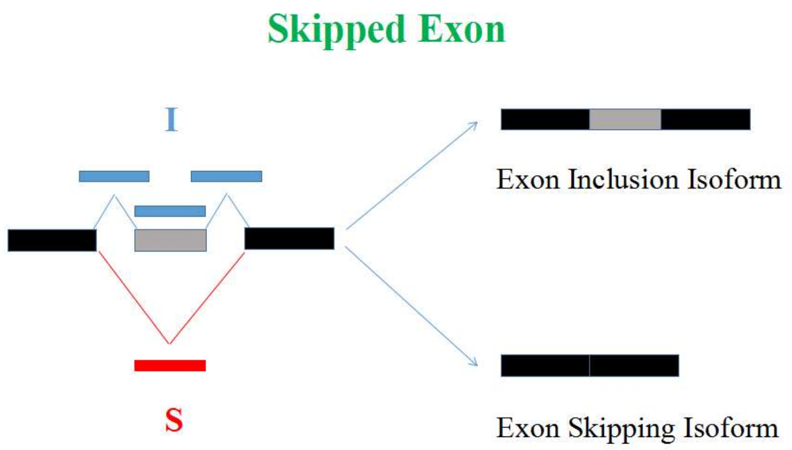

Figure 2.20 SE type of alternative splicing type

Note: The gray box in the figure indicates the exon that has a variable cut, and the black one is the constitutive exon.

Table 2.13 SE Type Differential Alternative Splicing Analysis Partial Results

GeneID	geneSymbol	chr	strand	exonStart_0base	exon

ENSG00000156299	TIAM1	21	-	31276731	312[illegible]
ENSG00000249624		21	+	33260596	332[illegible]
ENSG00000249624		21	+	33262792	332[illegible]
ENSG00000249624		21	+	33266011	332[illegible]
ENSG00000249624		21	+	33276595	332[illegible]

Showing 1 to 5 of 5 entries

Previous 1 Next

- **GeneID**: Gene id of the AS event.
- **geneSymbol**: Gene name of the AS event.
- **chr**: The chromosome where the AS event is located.
- **strand**: The direction of the chromosome chain where the AS event is located.
- **exonStart_0base**: The start position of AS event (the position that 1 nt upstream of the 5' end of the skipped exon)
- **exonEnd**: The end position of AS event (the 3' end position of skipped exon).
- **upstreamES**: The upstream exon start position of the AS event.
- **upstreamEE**: The upstream exon end position of the AS event.
- **downstreamES**: The downstream exon start position of the AS event.
- **downstreamEE**: The downstream exon end position of the AS event.
- **IC_SAMPLE_1**: Inclusion junction counts for SAMPLE_1, with only the reads span splicing junctions taken into account. Biological replicates are seperated by comma.
- **SC_SAMPLE_1**: Exclusion junction counts for SAMPLE_1, with only the reads span splicing junctions taken into account.
- **IC_SAMPLE_2**: Inclusion junction counts for SAMPLE_2, with only the reads span splicing junctions taken into account. Biological replicates are seperated by comma.
- **SC_SAMPLE_2**: Exclusion junction counts for SAMPLE_2, with only the reads span splicing junctions taken into account.
- **IncFormLen**: Length of inclusion form, used for normalization.
- **SkipFormLen**: Length of skipping form, used for normalization.
- **PValue**: Pvalue.
- **FDR**: Adjusted p-value.
- **IncLevel1**: Inclusion level for SAMPLE_1 replicates (comma separated) calculated from normalized counts.
- **IncLevel2**: Inclusion level for SAMPLE_2 replicates (comma separated) calculated from normalized counts.
- **IncLevelDifference**: average(IncLevel1) - average(IncLevel2).

2.7.2 Visualization of AS Events

Characteristics of Sashimi_plot as follows.

1. The RNA-Seq read densities along exons are shown as histograms, color-coded by the sample. The RNA-Seq densities are aligned to the isoforms drawn at the bottom of the plot, which are automatically read from the GFF annotation of the events given as input.
2. Junction reads are visualized as arcs connecting the pair of exons that the junction borders. The thickness of the arc is in proportion to the number of junction reads present in the sample, but the actual number of junction reads can be optionally plotted too (as in the main example.)
3. Vertical coordinate indicates a modified RPKM value

Result Directory: AS/2.dasplot (./result_tree.html)

SE1

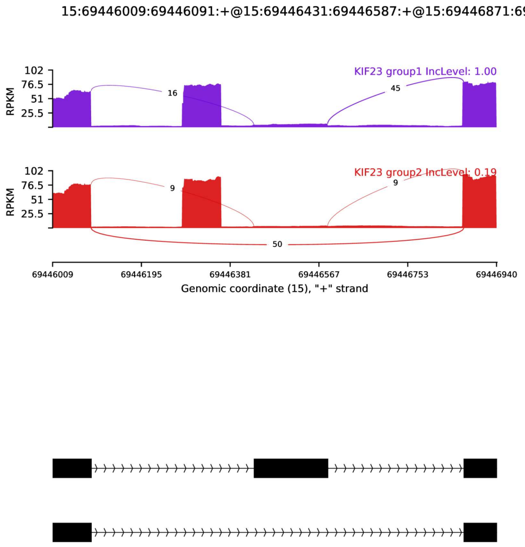

Figure 2.21 SE Type Alternative Splicing Visualization

Note: The red and orange colors in the figure represent different samples, and the inclusion level value represents the ratio of the total expression of exon inclusion isoform in the two isoforms, which can directly show the expression difference of alternative splicing events in different samples.

2.8 SNP&InDel Analysis

A single nucleotide polymorphism (SNP) is a substitution of nucleotide sequences occurring commonly within a population (e.g. 1%), in which a single nucleotide in the genome, or other shared sequence exchanges between members of a biological species or paired chromosomes. Two types of variation occur with SNPs, namely transitions and transversions, occur with a probability ratio of 1:2. The high occurrence region of SNPs is often the CG sequences, resulting in C to T transitions, which are associated with the tendency of C to be methylated. In contrast to SNPs, InDeL is a molecular biology term for an insertion or deletion of bases in the genome of an organism, measuring from 1 to 10 000 base pairs in length, which is classified among small genetic variations.

Mutation site analysis is an important part of RNA-seq structural analysis, including detection of inborn mutation sites and acquired somatic cell mutation sites, which is important for tumor research. We used GATK software to perform mutation site analysis on sample data and used Snpeff software to annotate the variant sites[18].

2.8.1 Variant Site Detection

The variant sites are divided into SNP and INDEL. The following table shows the SNP results,genotypes and annotations of each site. See the results file: SNP/2.snpeff (./result_tree.html).

Table 2.14 variant site type and its annotation

CHROM	POS	ID	REF	ALT	QUAL	DP	AD	GT	
1	14653	.	C	T	2298.03	141	0,74	1/1	E
1	14653	.	C	T	2298.03	141	0,74	1/1	E
1	14653	.	C	T	2298.03	141	0,74	1/1	E
1	14653	.	C	T	2298.03	141	0,74	1/1	E
1	15015	.	G	C	334.6	33	20,13	0/1	E

Showing 1 to 5 of 5 entries

Previous 1 Next

- **CHROM**: Chromosome ID of SNPs.
- **POS**: The postion of variation on chromosome.
- **ID**: The ID of this variation in dbSNP.
- **REF**: Bases of variation sites in reference genome.
- **ALT**: The actual base of the mutation site in the sample.
- **QUAL**: The quality of mutation sites, the value of phred scheme. The higher the value, the higher the reliability of the genotype.
- **DP**: The sequencing depth of each sample at the variant site (not all the number of reads, but the number of reads that are greater than a certain quality value.)
- **AD**: Allele Depth.The number of reads supporting either the reference genotype or SNP genotype.
- **GT**: The genotype of the locus.
- **GeneID**: Gene id where the variant site is located.
- **GeneName**: Gene name where the variant site is located.
- **FeatureID**: Transcript id where the variant site is located.
- **Biotype**: Transcript type where the variant site is located.
- **HGVS_C**: HGVS annotation of variant sites at the DNA level.
- **HGVS_P**: If variant is coding, this field describes the variant using HGVS notation (Protein level). Since transcript ID is already mentioned in ‘feature ID’, it may be omitted here.
- **EFFECT**: Detailed description of the effect predicted by SnpEff.
- **IMPACT**: Effects are categorized by ‘impact’: {High, Moderate, Low, Modifier}. This are pre-defined categories to help users find more significant variants.

2.8.2 Variant Site Statistics

After applying GATK for the detection of variant sites, we counted each variant site according to the SnpEff annotation information mainly including the function statistics of the variant site, the statistics of the variant site and the influence of the variant site. The variant site function (SNP_function) is statistically plotted from three aspects: synonymous mutation, missense mutation, and nonsense mutation. The variant site region (SNP_region) is statistically plotted from gene structure regions such as EXON, INTRON and INTERGENIC. Variant site influence (SNP_impact) is statistically plotted from four levels of HIGH (high), MODERATE (middle), LOW (low), MODIFIER (self-phenotype effect, and other variant sites should have an impact at the same time). The figure below shows the SNP results. See the result file: SNP/3.stat (./result_tree.html).

SNP_function

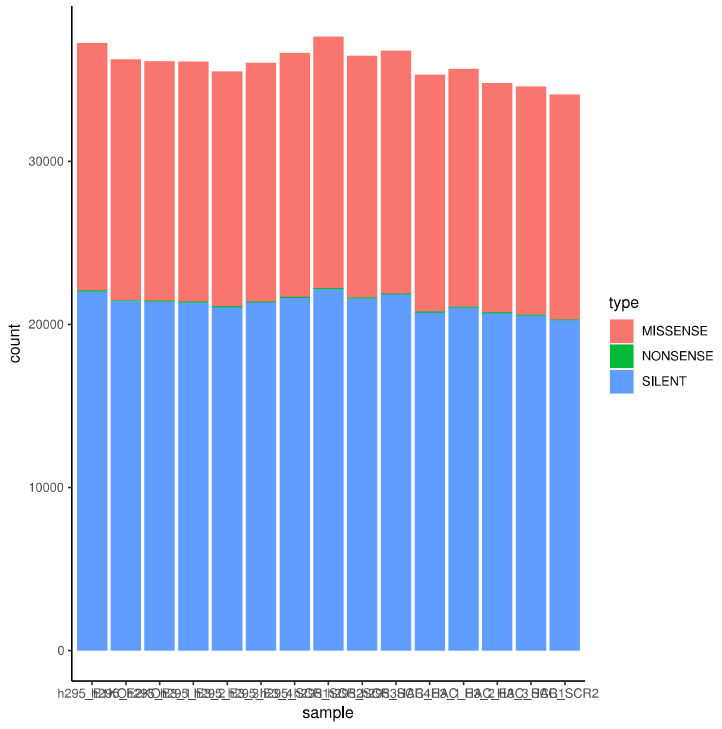

Figure 2.22 variability site statistics

Note: The abscissa in the figure represents the sample names, and the ordinate represents the number of variants of different genomic regions (EXON, INTRON, INTERGENIC)

Novogene Co.,Ltd.

2.8.3 Variant Site Analysis Q&A

What is the reference sequence for SNP analysis, and what is REF?

The reference sequence(REF) is the selected reference genomic sequence, and the SNP analysis used SNP calling by aligning the reads to the reference genome.

The two numbers in a column in the SNP represent the number of reads that support both REF and ALT bases. Why are some of them zero?

Taking 0,12 as an example, there are 0 reads that support REF bases, while there are 12 reads support ALT bases.

What does the SNP specifically mean? What is the difference between it and InDel?

In general, SNP refers to a single nucleotide variation with a frequency of variation greater than 1%. InDel(insertion-deletion) is an insert or deletion.

What is the meaning of the GT column?

GT indicates the genotype of a sample. Supposed that one genome of a diploid species has two copies, the same position of the two copies is an allele, and the GT value indicates the two alleles carried by the sample at a certain point. The point is mainly described by three values 0, 1, 2. 0 means the point is consistent with REF. 1 means it is consistent with the first ALT. 2 means it is consistent with the second ALT. 0/0 indicates homozygous. The two copies of the sample have the same alleles as REF. 0/1 indicates heterozygosity, and one copy of the sample is consistent with REF, and the other copy is consistent with ALT. 0/2 indicates heterozygosity. One copy of the sample is consistent with REF, and the other copy is consistent with the second ALT. 1/1 indicates homozygous and the alleles of the two copies of the sample are identical to the ALT. 1/2 indicates heterozygosity, and one copy of the sample is consistent with the first ALT, and the other copy is consistent with the second ALT. 2/2 means homozygous, and two copies of the sample are identical to the second ALT. According to genetic theory, the probability of two ALT appearing in one sample is very low.

What is the HGVS annotation?

HGVS is an abbreviation of Human Genome Variation Society. The gene variation sites are generally annotated with the command rules recommended by HGVS. For specific rules and examples, refer to DNA (http://www.hgvs.org/mutnomen/examplesDNA.html) and protein (http://www.hgvs.org/mutnomen/examplesAA.html)

2.9 Fusion Gene Analysis

A fusion gene is a chimeric gene in which all or part of two genes are fused together and is generally caused by chromosomal translocations, deletions and so on. The fusion gene was first discovered in malignant tumors of the blood system. Among them, the gene fusion of BCR-ABL in chronic myeloid leukemia is the most classical. The target of Imatinib/Gleevec, which treats chronic myeloid leukemia, is the fusion gene. High-throughput RNA sequencing technology has greatly accelerated the study of fusion genes due to its high throughput, low cost, high detection accuracy and wide detection range. Novogene uses STAR-Fusion (https://github.com/STAR-Fusion/STAR-Fusion/wiki) which is a software package that uses the fusion output of STAR alignment to detect the fusion transcript. The STAR-Fusion analysis process is shown in the figure below. It is divided into SATR alignment, STAR-Fusion.predict, STAR-Fusion.filter, the specific principle can refer to the appendix.

2.9.1 Fusionlist

The fusionlist is shown below. JunctionReads and SpanningFrags are the main indicators for detecting fusion genes; in pair-end sequencing, read1 and read2 can be regarded as the ends of a fragment (250bp-300bp). If read1 or read2 crosses the breakpoint of the fusion event, the read is called JunctionRead. The gap area between read1 and read2 spans the breakpoint of the fusion event. Such a fragment is called SpanningFrag. See the result file: Fusion/1.fusionlist (./result_tree.html).

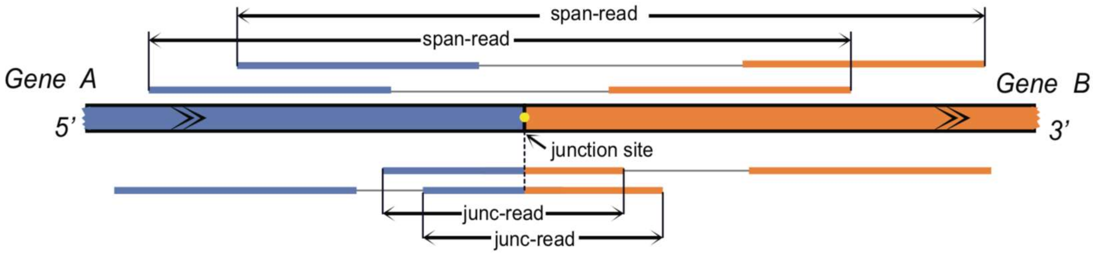

Figure 2.29 JunctionReads and SpanningFrags Schematic

See the result file: Fusion/1.fusionlist (./result_tree.html).

FusionName	JunctionReadCount	SpanningFragCount	
U2AF1--SH3TC1	59	3	U2AF1^E[illegible]
TMCC1--AC055733.2	39	0	TMCC1^E[illegible]
PRH1--AC007450.1	26	0	PRH1^E[illegible]
TAS2R14--AC007450.1	26	0	TAS2R14^[illegible]
AL391840.3--SH3BGRL2	24	1	AL391840.3[illegible]

Showing 1 to 5 of 5 entries

Previous 1 Next

- **FusionName**: Fusion event name.
- **JunctionReadCount**: The number of junction reads that support fusion events.
- **SpanningFragCount**: The number of Spanning fragments that support fusion events.
- **LeftGene**: The upstream gene of fusion event, including the gene ID and the gene name, wherein the gene ID is the GENCODE database ID, and the decimal point and the following numbers are removed to be consistent with the previous ones.
- **LeftLocalBreakpoint**: The breakpoint position of the upstream gene on the gene sequence (the breakpoint position starts from the 5' end of each gene sequence).
- **LeftBreakpoint**: The genomic coordinates of the upstream gene breakpoint and the positive and negative strand information of the genome of the upstream gene.
- **RightGene**: The downstream gene of fusion event.
- **RightLocalBreakpoint**: The breakpoint position of the downstream gene on the gene sequence (the breakpoint position starts from the 5' end of each gene sequence).
- **RightBreakpoint**: The genomic coordinates of the downstream gene breakpoint and the positive and negative strand information of the genome of the upstream gene.
- **SpliceType**: Whether the identified fusion breakpoint is located in an exon splice site of a known gene structure.
- **annots**: Functional annotation information for upstream and downstream genes of fusion events.
- **CDS_LEFT_ID**: Transcript ID with CDS region in the upstream gene.
- **CDS_LEFT_RANGE**: The base range(bp) of transcript with CDS regions in the upstream gene.
- **CDS_RIGHT_ID**: Transcript ID with CDS region in the downstream gene.
- **CDS_RIGHT_RANGE**: The base range(bp) of transcript with CDS regions in the downstream gene.
- **PROT_FUSION_TYPE**: The fusion type of the coding gene, FRAMESHIFT (shifting of the reading frame), INFRAME (the frame does not have a frameshift).
- **FUSION_CDS**: The CDS sequence of the fusion gene, the lower case base is the CDS sequence of the upstream fusion gene, and the upper case is the CDS sequence of the downstream fusion gene.
- **FUSION_TRANSL**: The amino acid sequence of the translated fusion gene.
- **PFAM_LEFT**: The PFAM annotation of the upstream gene, such as DUF1515|572-611|0.073, where DUF1515 is the predicted protein domain of PFAM, 572-611 is the base range of the predicted domain, and 0.073 is the similarity between prediction and DUF15.
- **PFAM_RIGHT**: The PFAM annotation of the downstream gene.

2.9.2 The Distribution of Fusion Gene on Genome

The distribution of fusion gene events on the chromosome is shown by a circos diagram. The loop in the figure consists of multiple chromosomes. Each curve in the loop represents a fusion event. The two ends of the curve represent the breakpoint position of the fusion event. The red indicates that the upstream and downstream genes of the fusion gene are derived from the same

chromosome. While blue indicates that the upstream and downstream genes of the fusion gene are derived from different chromosomes. The thickness of the curve represents the expression level of the fusion gene. See the result file: Fusion/2.circos (./result_tree.html).

h295_E1KO

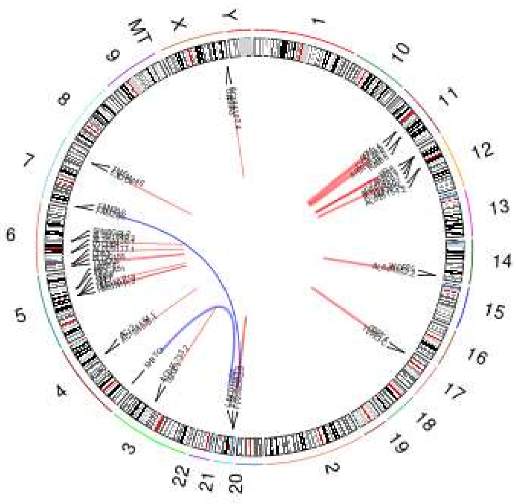

Figure 2.30 Fusion Gene Chromosome Distribution

2.9.3 Visualization of Fusion Gene

Fusion gene events can be visualized by IGV software as shown below.

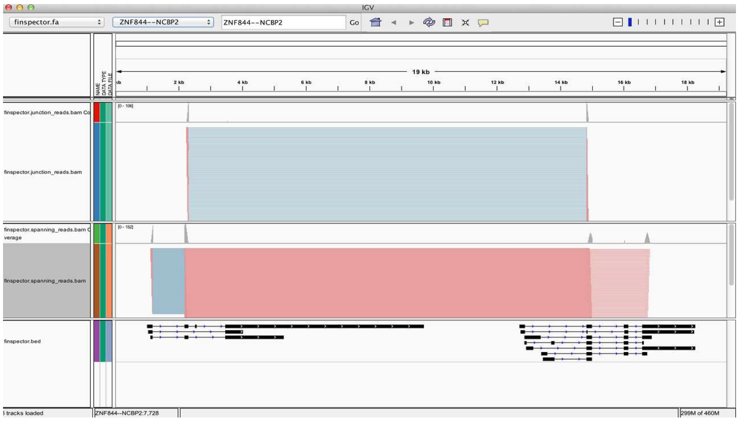

Figure 2.31 Fusion Gene Chromosome Distribution

Import The fusion gene sequence file of each sample in the fusion gene folder in the result file, the gene structure annotation file (providing annotation files in both bed and gtf formats, one of which can be used) and the aligned bam files are respectively into IGV software. The specific

file description is as follows, see the result file: Fusion/3.IGV (./result_tree.html).

- **sample.fa**: Fusion gene sequence file, in fasta format.
- **sample.bed**: Fusion gene structure annotation file, in bed format.
- **sample.gtf**: Fusion gene structure annotation file, in gtf format.
- **sample.junction_reads.bam**: Bam files for JunctionReads that support fusion events.
- **sample.spanning_reads.bam**: Bam files for SpanningFrags that support fusion events.

2.9.4 Fusion Gene Q&A

Which fusion gene should be selected for fusion gene validation?

Highly expressed fusion genes are preferentially selected for validation. Highly expressed fusion gene events have lower false positives and are more easily experimentally verified. However, low expression fusion gene events also have research values. For example, many tumor suppressor genes are lowly expressed and the fusion of these genes may result in the loss of important functions.

3 Appendix

3.1 Result file decompression method

Compressed.format	Customer.type	method
**compressed files in the format of *.tar.gz:**	Unix/Linux/Mac user	use tar -zxvf *.tar.gz command
	Windows user	use uncompressed software such as WinRAR, 7-Zip et al
**compressed files in the format of *.gz:**	Unix/Linux/Mac user	use gzip –d *.gz command
	Windows user	use uncompressed software such as WinRAR, 7-Zip et al
**compressed files in the format of *.zip:**	Unix/Linux/Mac user	use unzip *.zip command
	Windows user	use uncompressed software such as WinRAR, 7-Zip et al

3.2 Result file format description

File.type	Document.description	Open.mode
**file.fa/fasta**	sequence file, in the format of fasta in genaral. Since sequence of gene or genome is large, we provide customer with sample*.fasta files(partial sequences of* .fasta). It will be more convenient to check file fomat.	unix/Linux/Mac users use less or more commands to view sequences in the format of *.fasta.
		windows users use editor Editplus/Notepad++ et al
**file.fq/fastq**	eads sequence file, in the format of fasta. it is not easy to open since it is a large big file.	unix/Linux/Mac users use less or more commands;
		windows users use editor Editplus/Notepad++ et al
**file.txt/xls**	table result file; files are separated by(Tab)	unix/Linux/Mac users use less or more commands
		windows users use editor Editplus/Notepad++ et al, also can use Microsoft Excel to open.
**file.pdf/svg**	Results image files and vector images can be enlarged and reduced without distortion, which is convenient for users to view and edit. Adobe Illustrator can be used to edit images and publish articles	Windows / Mac users can use adobe reader / foxin reader / Web browser to open it
		Unix / Linux users use evince command to open
**file.png**	Results image file; bitmap, lossless compression	Unix / Linux / Mac users use the display command to open
		windows users use picture viewer to view, for example Photoshop etc. al

3.3 Analysis software list and version

Analysis	Software	Version	Parameter	Remarks
**Mapping**	hisat2	2.0.5	Default	Mapping to a reference
**Assembly**	Stringtie	1.3.3b	Transcripts of class code type 'u'	
**Quantification**	featureCounts	1.5.0-p3	Default	
**Differential Analysis**	DESeq2	1.20.0	|log2(FoldChange)| >= 1 & padj <= 0.05	For sample with bio-replicate
	edgeR	3.22.5	|log2(FoldChange)| >= 1 & padj <= 0.05	For sample without bio-replicate
**Enrichment Analysis**	clusterProfiler	3.8.1	padj < 0.05	For GO, KEGG enrichment analysis
**GSEA Analysis**	gsea	v3.0		
**Protein-Protein Interaction Analysis**	diamond	0.9.14	Using blast, String database.	
**Alternative splicing**	rMATS	4.1.0	Default	
**SNP/InDel Analysis**	GATK	4.1.1.0	MQ < 40.0 and QD < 2.0 and FS > 30.0 and DP < 10 and QUAL < 20	SNP/InDel calling
	snpEff	4.3.1q	Default	SNP/InDel Annotation

3.4 methods

In order to facilitate users to write articles, we have prepared data analysis related toEnglishmethods (src/methods.pdf)

3.5 references

- [1] Wang Z, Gerstein M, Snyder M. RNA-Seq: a revolutionary tool for transcriptomics[J]. Nature Reviews Genetics, 2009, 10(1): 57-63.

- [2] Parkhomchuk D, Borodina T, Amstislavskiy V, et al. Transcriptome analysis by strand-specific sequencing of complementary DNA[J]. Nucleic acids research, 2009, 37(18): e123-e123.
- [3] Goldstein L D , Cao Y , Pau G , et al. Prediction and Quantification of Splice Events from RNA-Seq Data.[J]. Plos One, 2016, 11(5):e0156132.
- [4] Mortazavi A, Williams B A, McCue K, et al. Mapping and quantifying mammalian transcriptomes by RNA-Seq[J]. Nature methods, 2008, 5(7): 621-628.
- [5] Mihaela Pertea, Geo M Pertea, Corina M Antonescu1,et al.StringTie enables improved reconstruction of a transcriptome from RNA-seq reads[J].Nat Biotechnol. 2015 March ; 33(3):290-295(StringTie)
- [6] Liao Y1, Smyth GK, Shi W. featureCounts: an efficient general purpose program for assigning sequence reads to genomicfeatures.Bioinformatics.2014 ,30(7):923-30.(featureCounts)
- [7] Garber M, Grabherr M G, Guttman M, et al. Computational methods for transcriptome annotation and quantification using RNA-seq[J]. Nature methods, 2011, 8(6): 469-477.
- [8] Bray N, Pimentel H, Melsted P, et al. Near-optimal RNA-Seq quantification[J]. arXiv preprint arXiv:1505.02710, 2015.
- [9] Patro R, Mount S M, Kingsford C. Sailfish enables alignment-free isoform quantification from RNA-seq reads using lightweight algorithms[J]. Nature biotechnology, 2014, 32(5): 462-464.
- [10] Anders S, Huber W. Differential expression analysis for sequence count data[J]. Genome biol, 2010, 11(10): R106.
- [11] Love M I, Huber W, Anders S. Moderated estimation of fold change and dispersion for RNA-seq data with DESeq2[J]. Genome biology, 2014, 15(12): 1-21.(DESeq2)
- [12] Robinson M D, McCarthy D J, Smyth G K. edgeR: a Bioconductor package for differential expression analysis of digital gene expression data[J]. Bioinformatics, 2010, 26(1): 139-140.(edgeR)
- [13] Young M D, Wakefield M J, Smyth G K, et al. Method Gene ontology analysis for RNA-seq: accounting for selection bias[J]. Genome Biol, 2010, 11: R14.
- [14] He Z , Zhao X , Lu Z , et al. Comparative transcriptome and gene co-expression network analysis reveal genes and signaling pathways adaptively responsive to varied adverse stresses in the insect fungal pathogen, Beauveria bassiana[J]. Journal of Invertebrate Pathology, 2017:S0022201117304391.
- [15] Kanehisa M, Goto S. KEGG: kyoto encyclopedia of genes and genomes[J]. Nucleic acids research, 2000, 28(1): 27-30.(KEGG)
- [16] Shen S., Park JW., Lu ZX., Lin L., Henry MD., Wu YN., Zhou Q., Xing Y. rMATS: Robust and Flexible Detection of Differential Alternative Splicing from Replicate RNA-Seq Data.(rMATS).
- [17] Katz Y, Wang E T, Airoldi E M, et al. Analysis and design of RNA sequencing experiments for identifying isoform regulation[J]. Nature methods, 2010, 7(12): 1009-1015.
- [18] McKenna A, Hanna M, Banks E, et al. The Genome Analysis Toolkit: a MapReduce framework for analyzing next-generation DNA sequencing data[J]. Genome research, 2010, 20(9): 1297-1303.(GATK)
