# Supplementary material for: ELAVL1-KO Affects Steroid Synthesis in ACC Cell Line NCI-H295R and Reduces Colony-Forming Abilities
Source: Int J Mol Sci. 2026 Aug 4;27(15):7001. doi: 10.3390/ijms27157001 (PMC13467342; doi:10.3390/ijms27157001)

Original Figures ELAVL1

2B Western Blot

ELAVL1+Histone H3

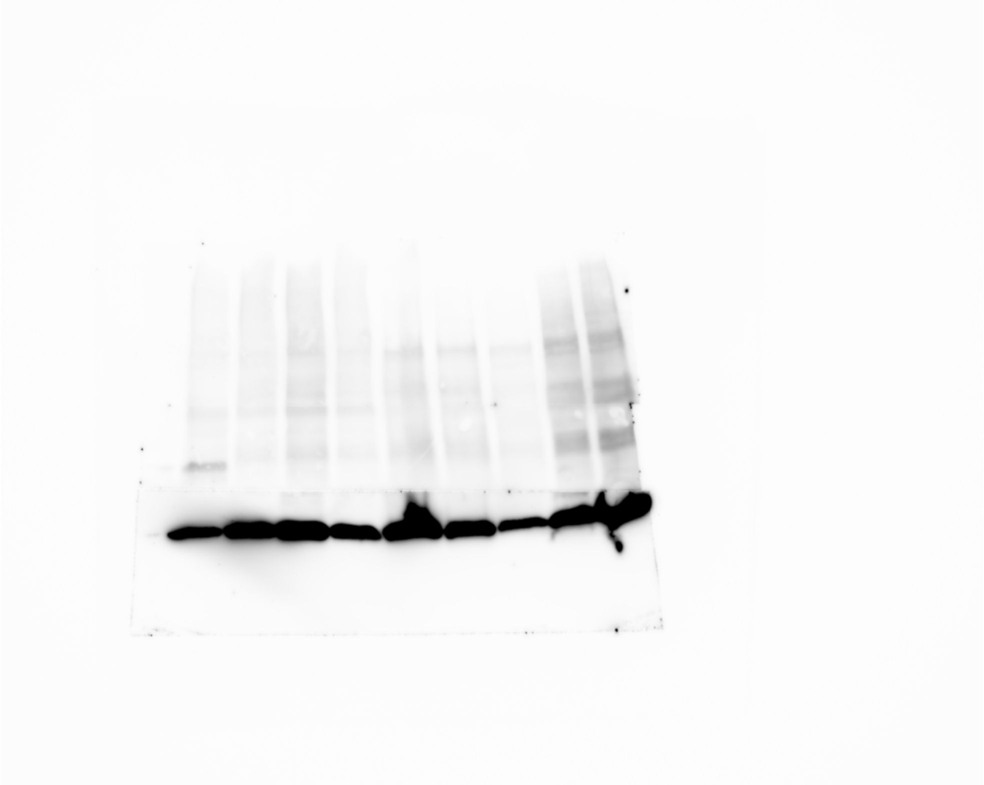

ELAVL1

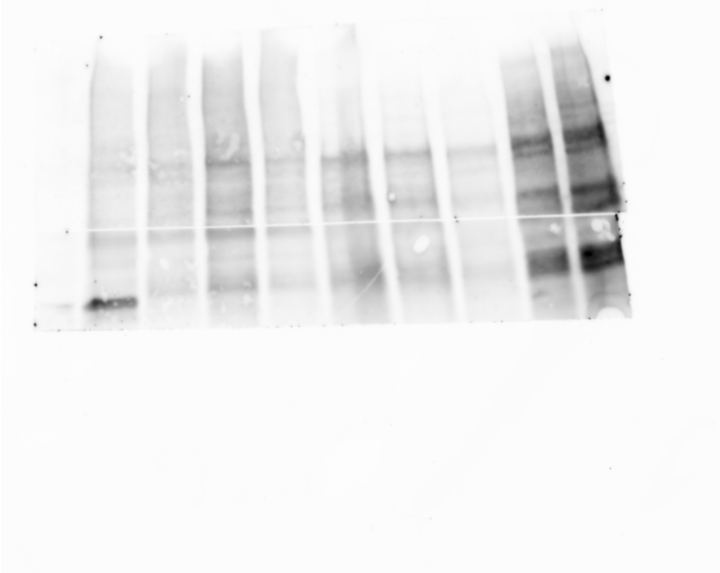

2C Immunofluorescence

Scr:

Scr DAPI

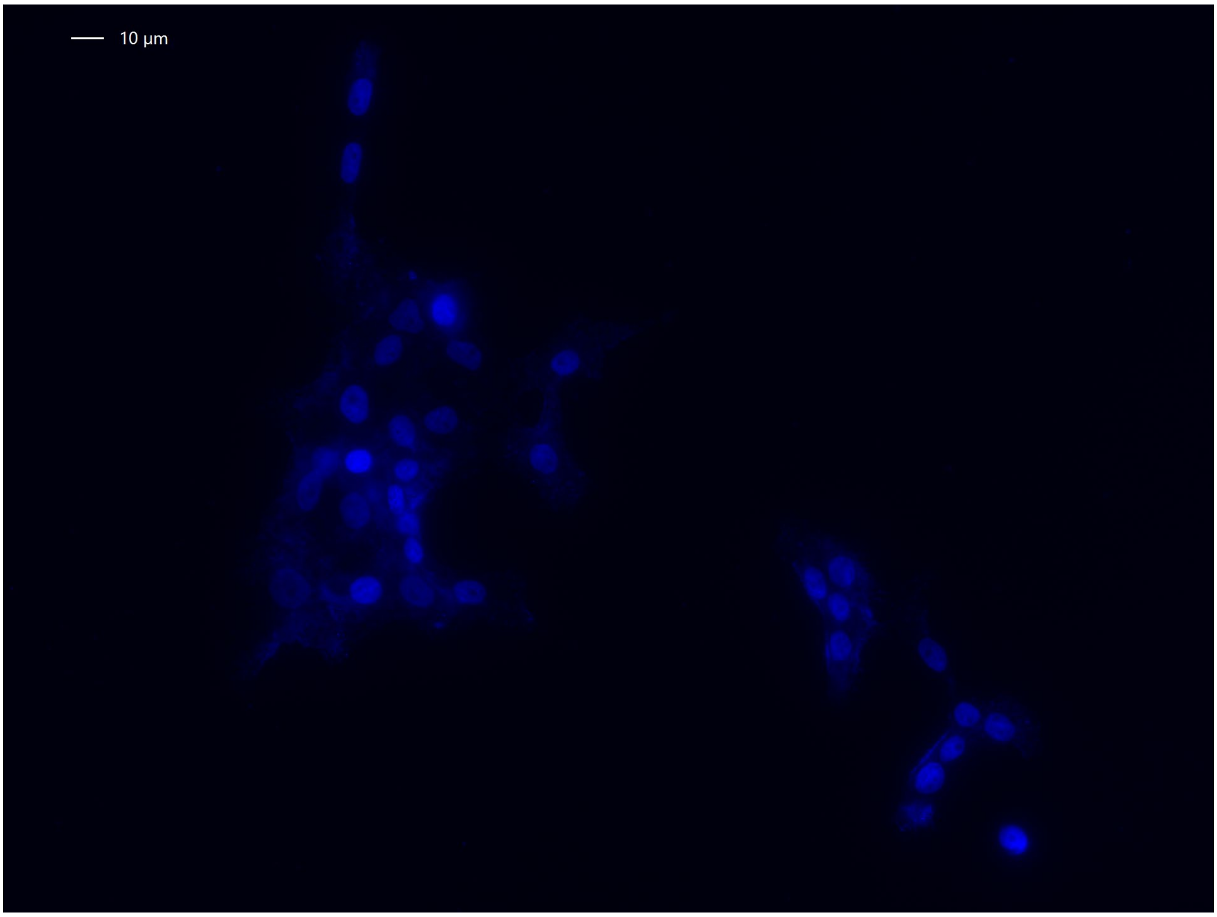

Scr Phalloidin

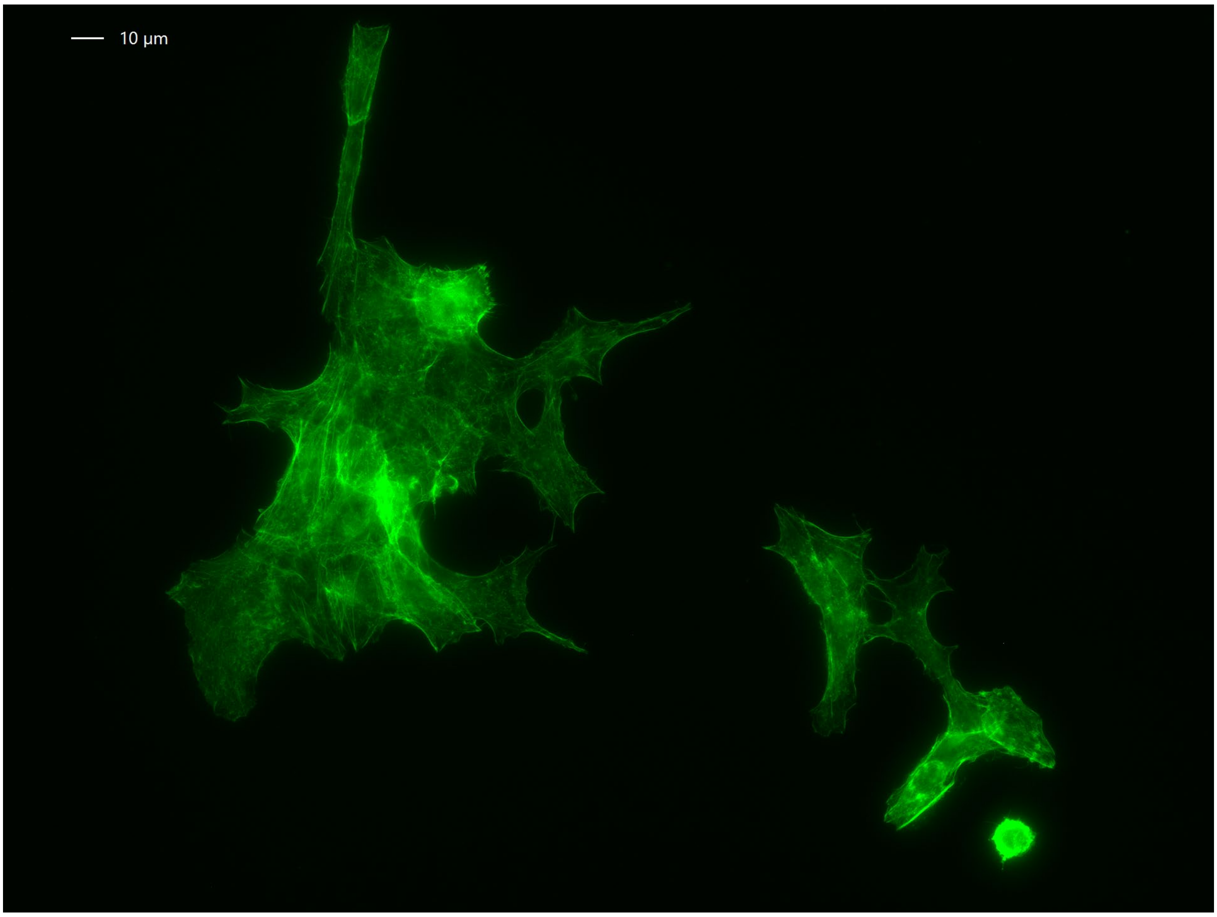

Scr ELAVL1

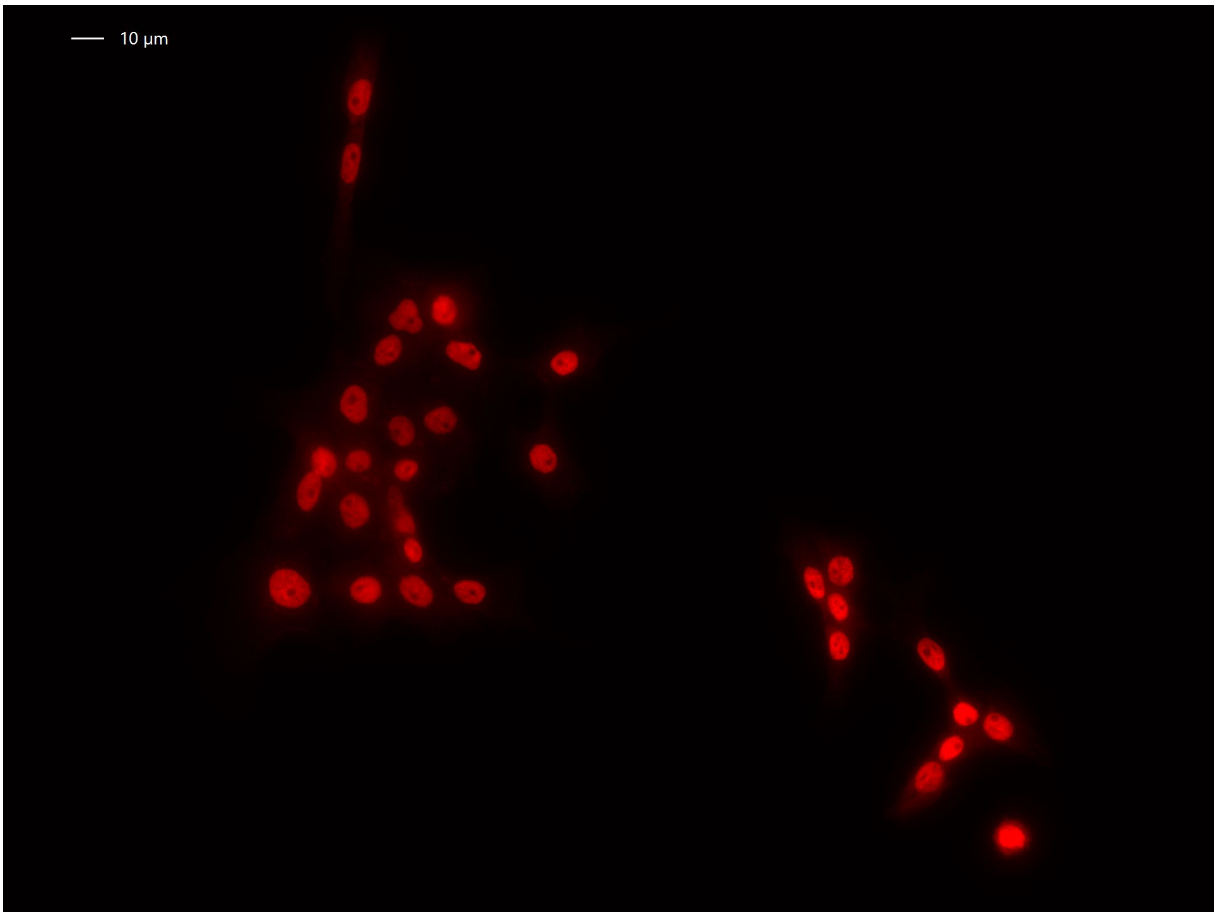

Scr Merge

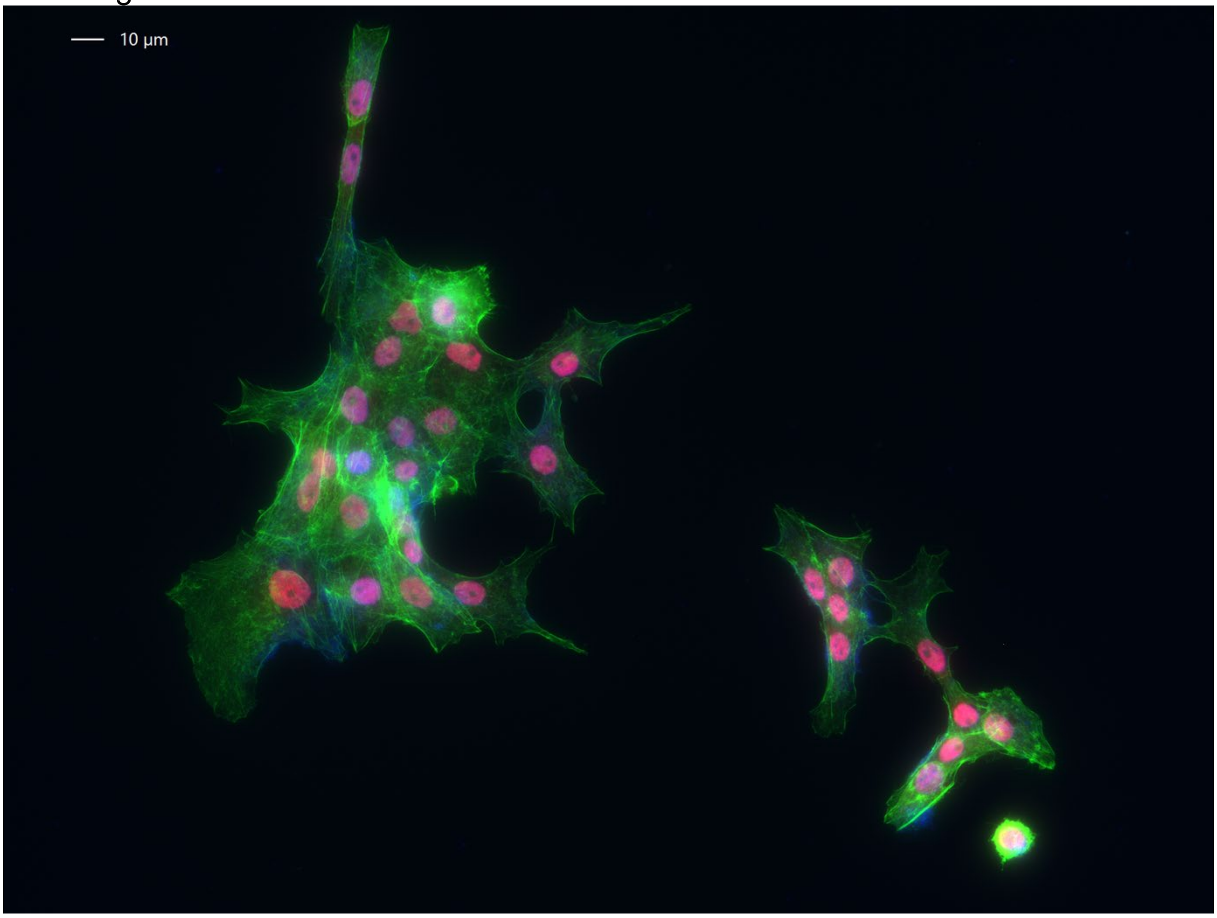

gRNA 3 KO-MC (mixed culture):

gRNA 3 KO-MC_DAPI

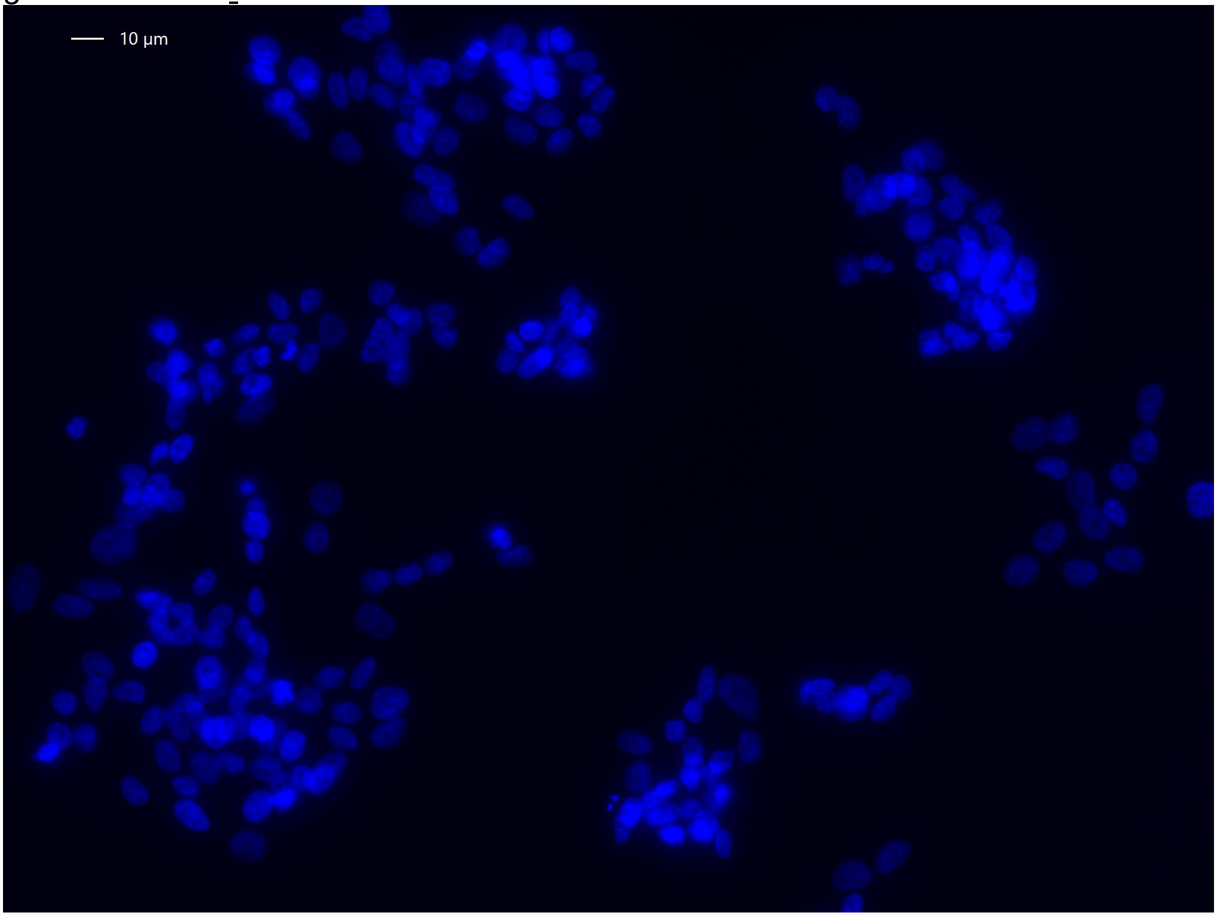

gRNA 3 KO-MC Phalloidin

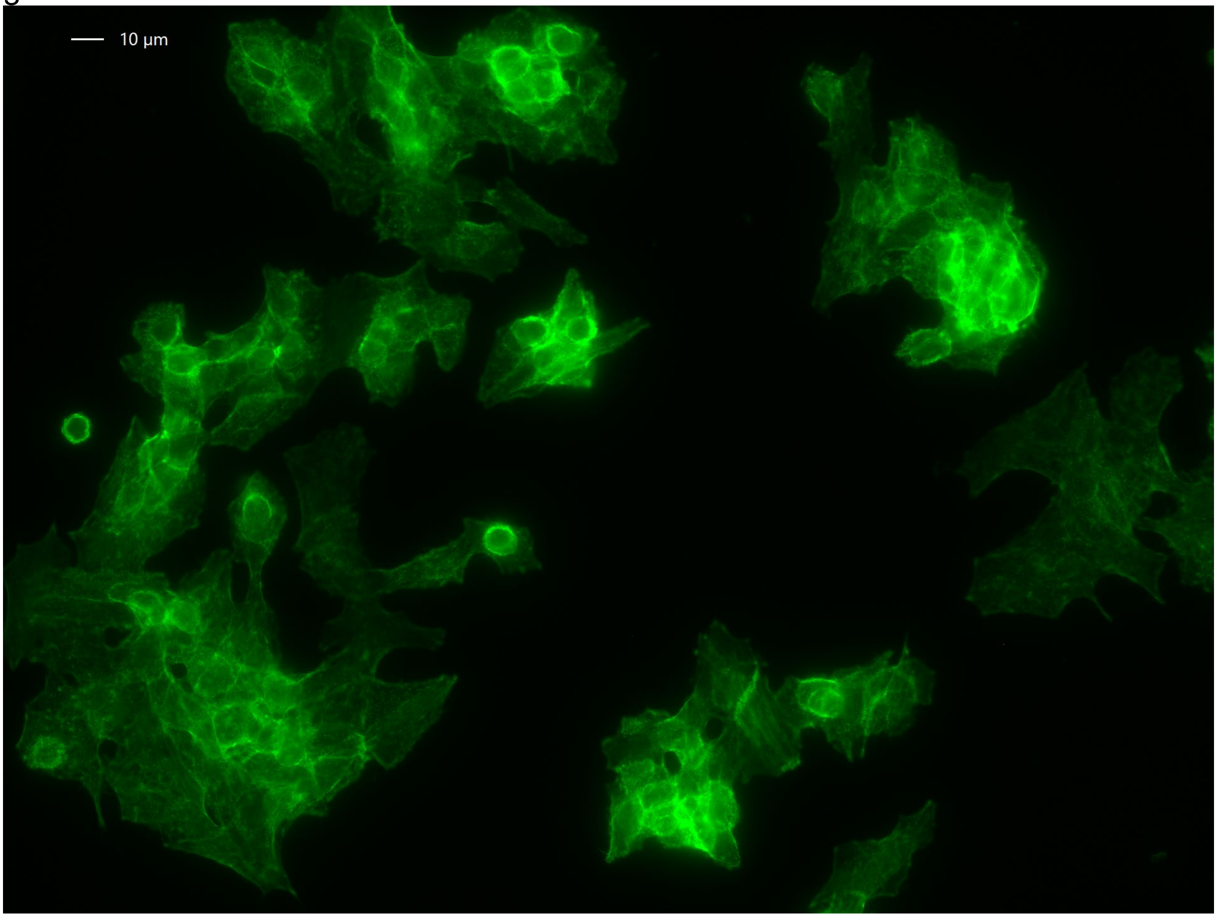

gRNA 3 KO-MC ELAVL1

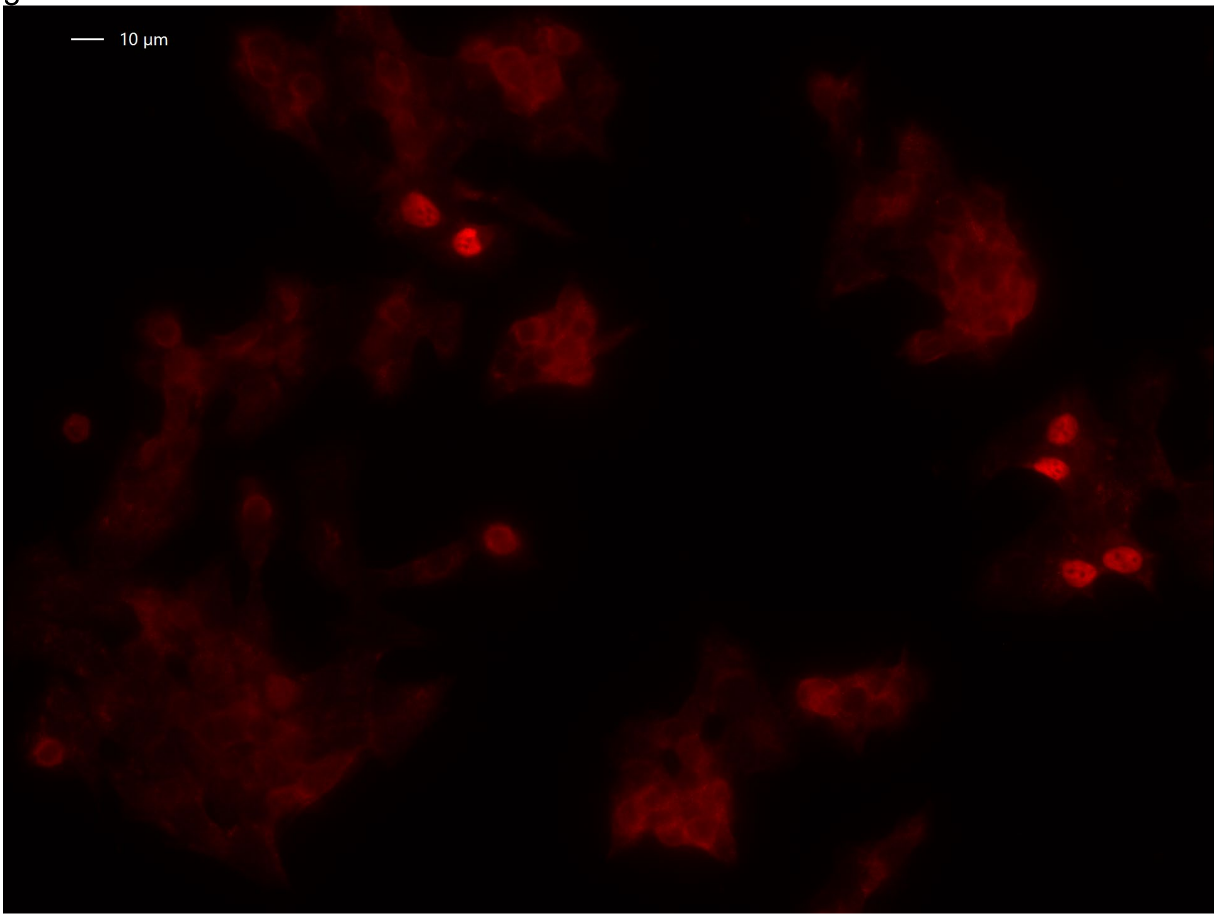

gRNA 3 KO-MC Merge

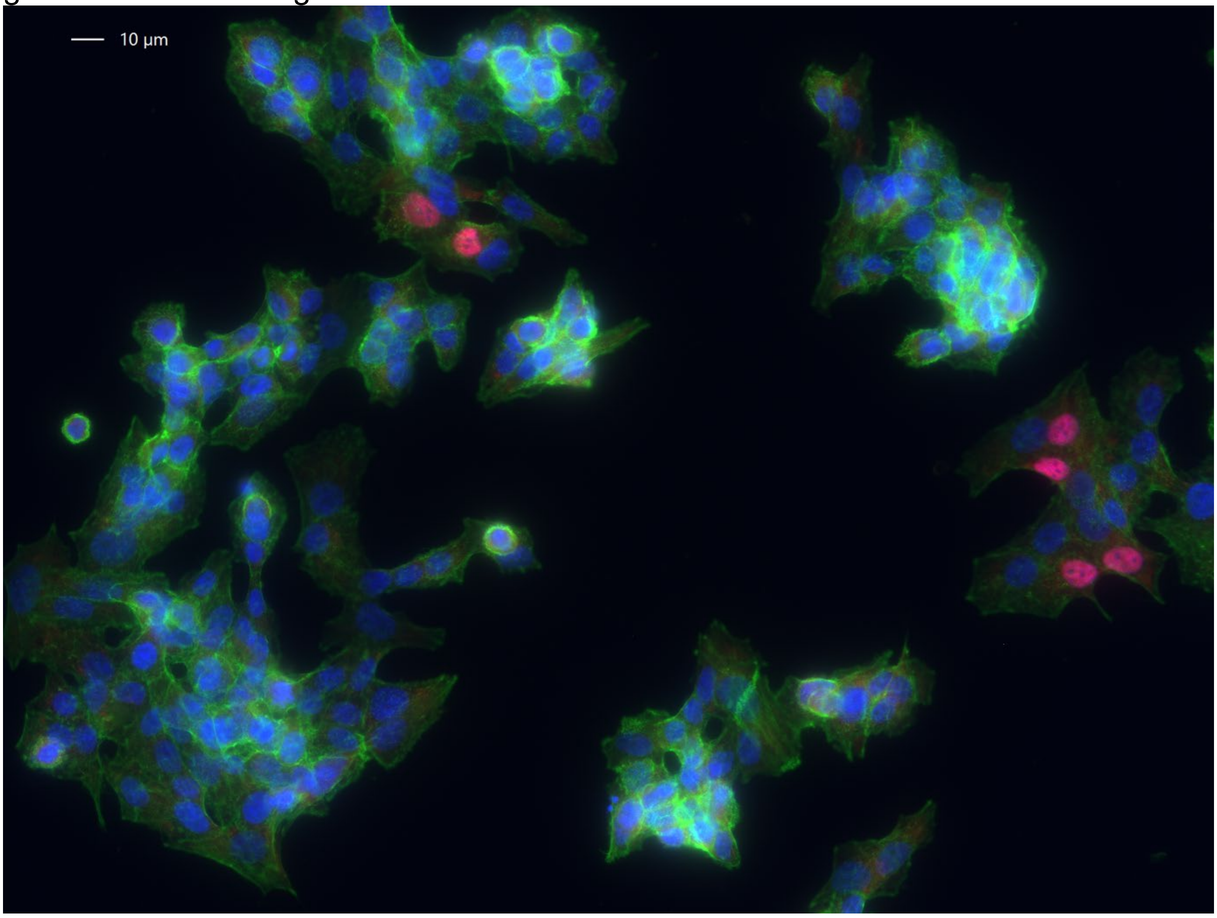

<u>gRNA3 KO-SCC (single cell clone):</u>
gRNA3 KO-SCC DAPI

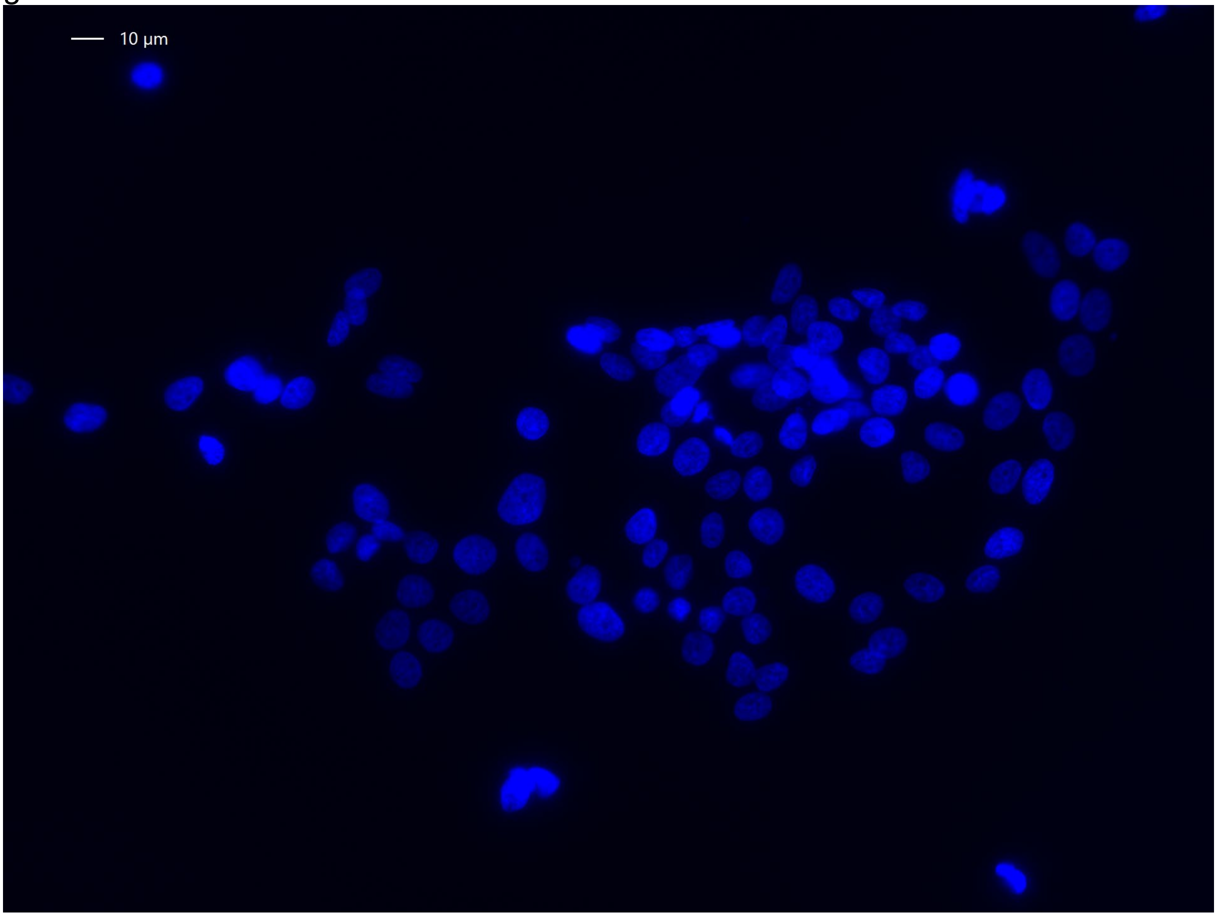

gRNA3 KO-SCC Phalloidin

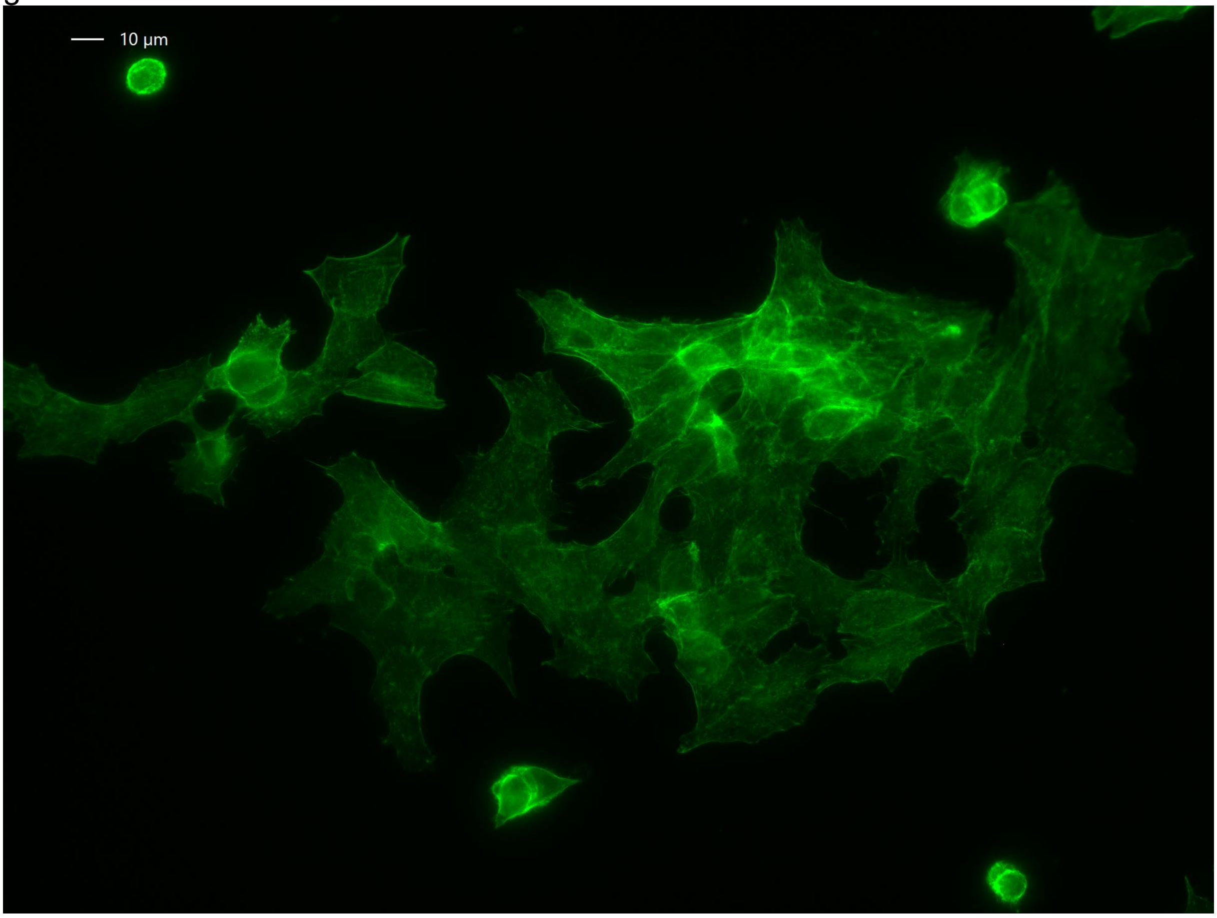

gRNA3 KO-SCC ELAVL1

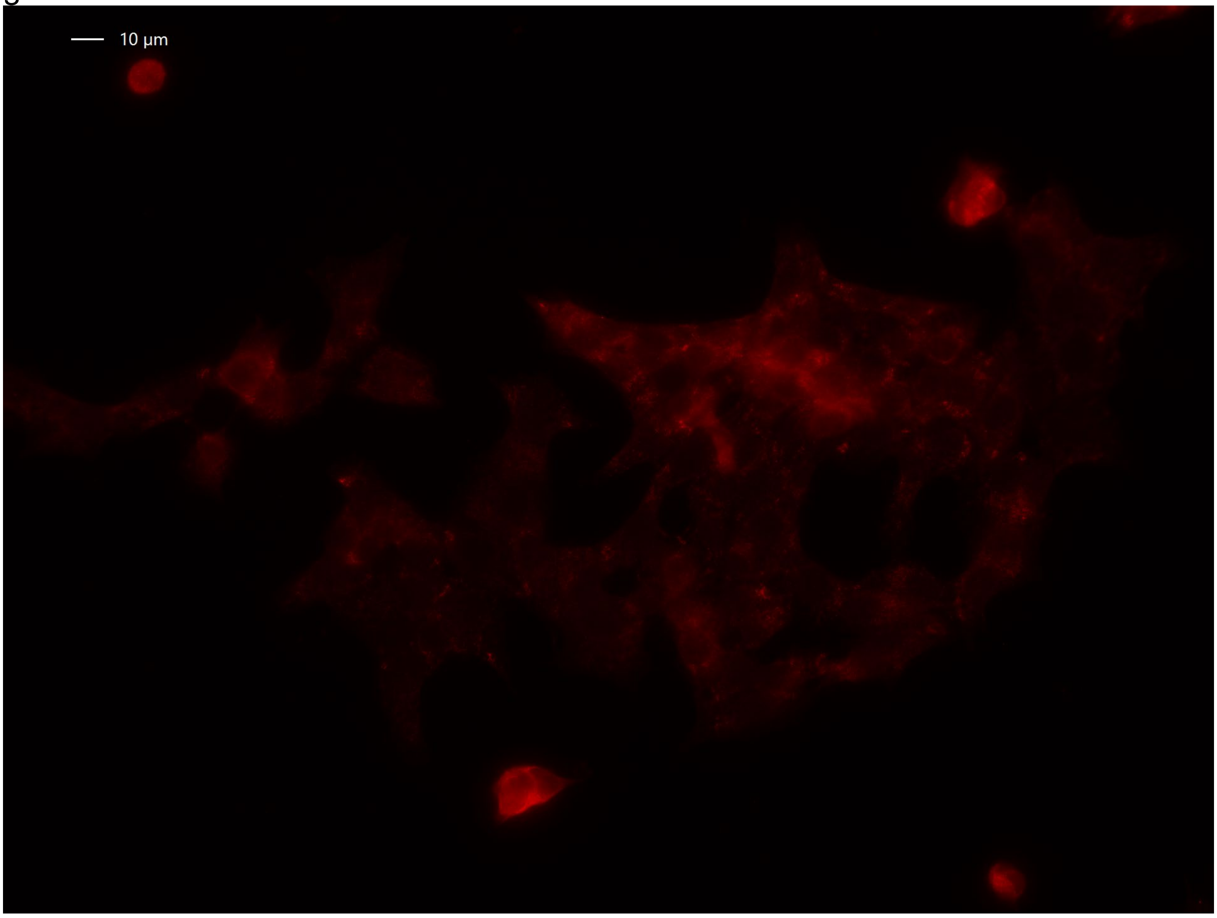

gRNA3 KO-SCC Merge

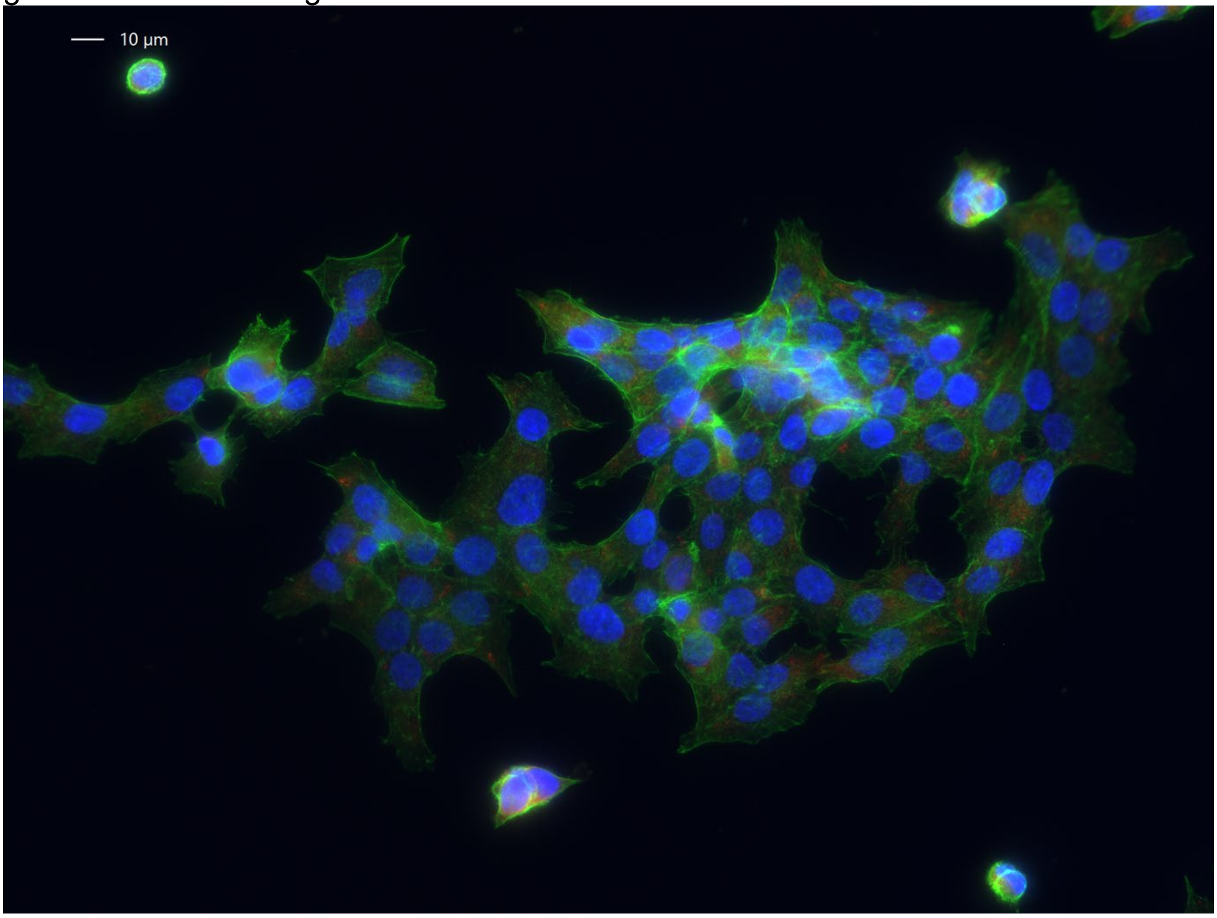

3A Soft agar colony forming assay

Scr

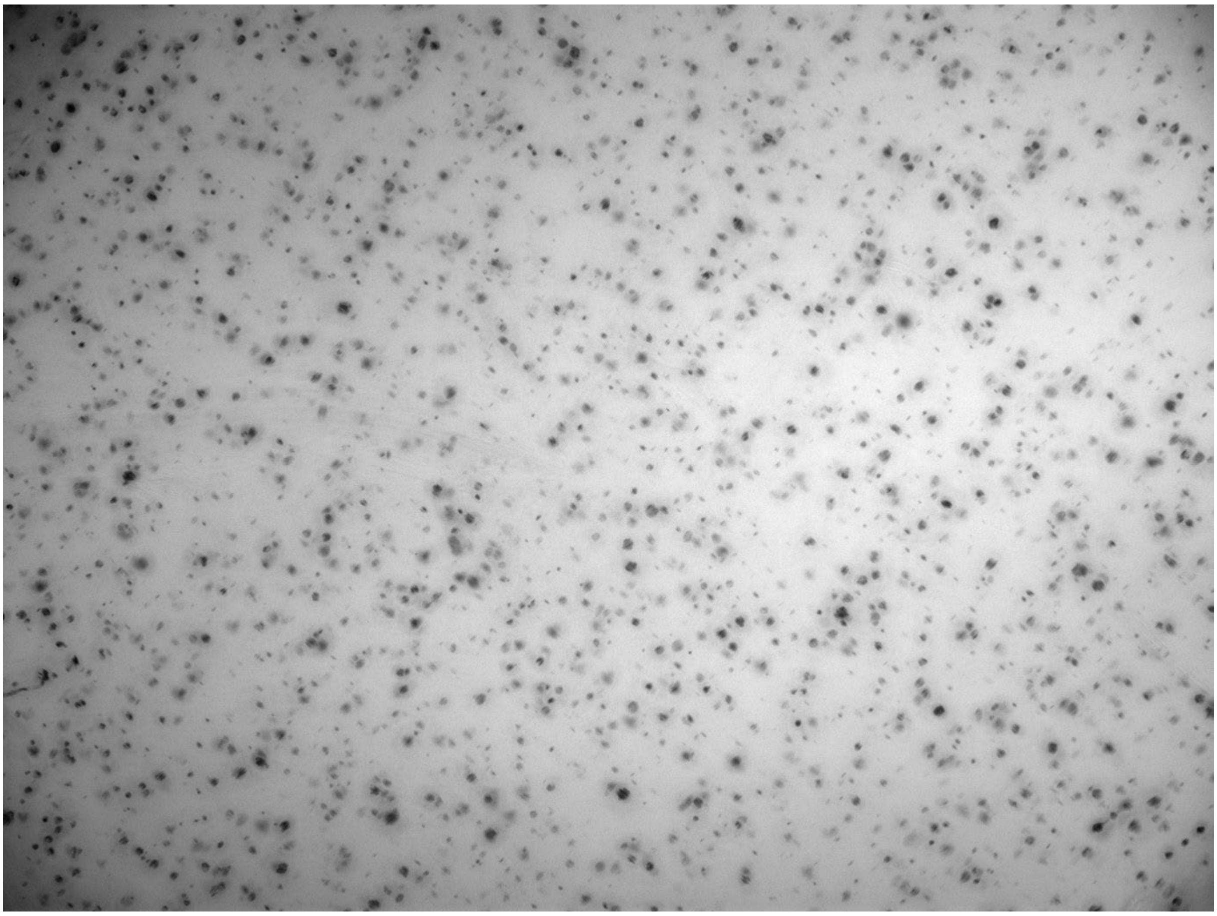

KO

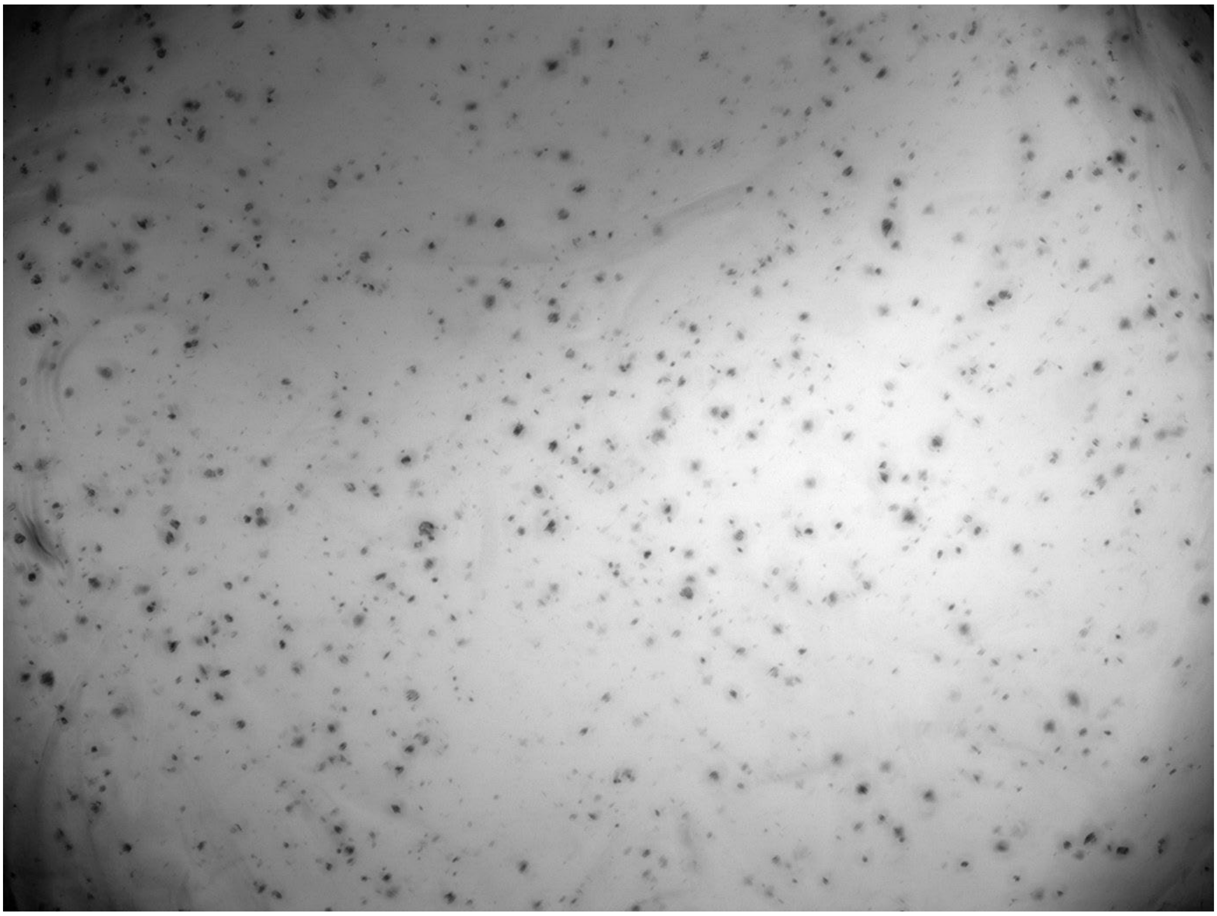

6B PCR

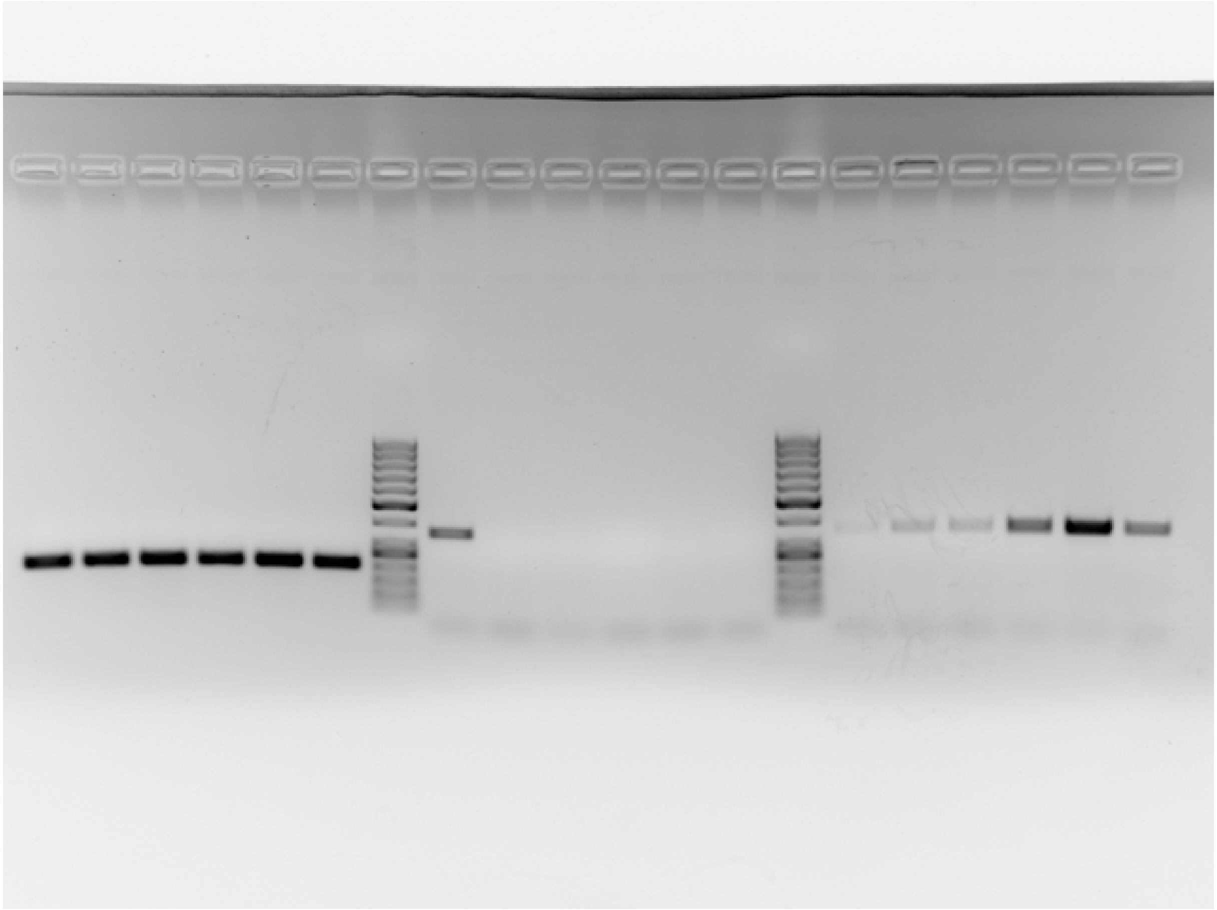

Supplement: Supplementary file 1 [file ijms-27-07001-s001.zip › Original Figures ELAVL1.pdf]
