# Supplementary material for: ELAVL1-KO Affects Steroid Synthesis in ACC Cell Line NCI-H295R and Reduces Colony-Forming Abilities
Source: Int J Mol Sci. 2026 Aug 4;27(15):7001. doi: 10.3390/ijms27157001 (PMC13467342; doi:10.3390/ijms27157001)
Supplement: Supplementary file 1 [file ijms-27-07001-s001.zip › Supplement_Data and methods.pdf]

Supplemental Data.

Supplementary Table S1:
ELAVL1 genomic Primer gRNA 1: Sense: CAAGAGCAATGGACAGAGTTTG
Antisense: AATGCAGTTACTAGTTTTGCCTTC
ELAVL1 genomic Primer gRNA 2 and 3 Sense: AGTGGGTCCTGAGACACTCAT
Antisense: GGAACCTGTGTTCATTGCAGA

Off Target effects:
Target sequence gRNA 2: TGTGAACTACCTGACCGCGAAGG

Nr.	Lokalisation (Genom)	Anzahl Mismatches	Sequenz (inkl. Mismatches)
1	chr17:33229930	3	TGTGAACTACTGTAACcCtGAAGG
2	chr19:43175908	3	CCATcCGGTcCcGTAtTTCACA
3	chr6:107708700	3	TGTGAACTACTCcCTGACCGCaAAGG

Target sequence gRNA3: TCAACACGCTGAACGGCTTGAGG

Nr.	Lokalisation (Genom)	Anzahl Mismatches	Sequenz (inkl. Mismatches)
1	chr5:79134206	3	TCAgCACaCTGAAGAGGCTTGAGG
2	chr8:52892779	3	TCAcCACCGTGAggGGCTTGAGG

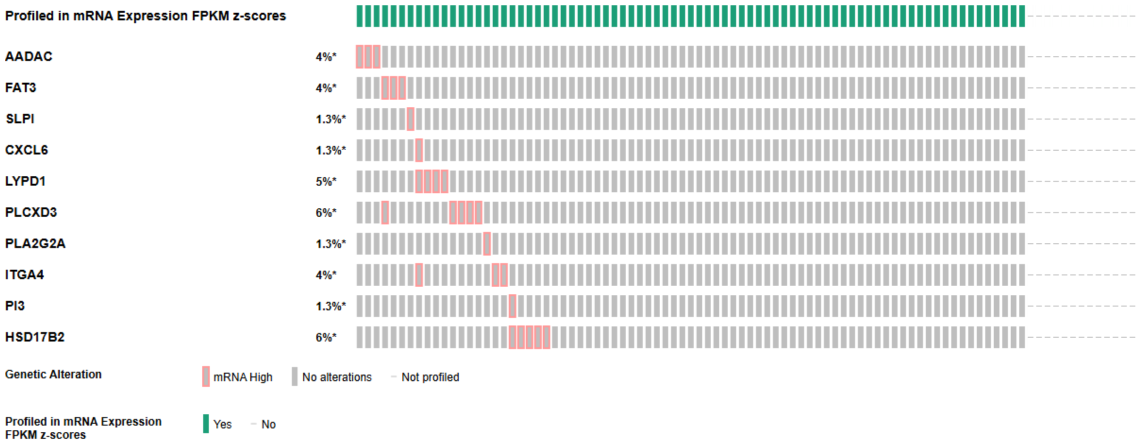

Supplemental Figure S1: Alterations in the expression level of the top down regulated genes identified with cBioPortal tool.

Supplemental Table S2: Correlation of clinical parameters with alterations in gene expression.

Clinical Attribute	Attribute Type	Statistical Test	p-Value	q-Value
Fraction Genome Altered	Sample	Wilcoxon Test	2.59E-04	6.98E-03

Race Category	Patient	Chi-squared Test	0.206	0.867
AJCC Clinical M-Stage	Patient	Chi-squared Test	0.213	0.867
Prior Malignancy	Patient	Chi-squared Test	0.288	0.867
AJCC Pathologic T-Stage	Patient	Chi-squared Test	0.316	0.867
AJCC Pathologic N-Stage	Patient	Chi-squared Test	0.357	0.867
AJCC Pathologic Stage	Patient	Chi-squared Test	0.37	0.867
Other Sample ID	Sample	Chi-squared Test	0.447	0.867
Other Patient ID	Patient	Chi-squared Test	0.447	0.867
Sex	Patient	Chi-squared Test	0.454	0.867
Birth from Initial Pathologic Diagnosis Date	Patient	Wilcoxon Test	0.62	0.867
Morphology	Patient	Chi-squared Test	0.628	0.867
Diagnosis Age	Patient	Wilcoxon Test	0.631	0.867
Prior Treatment	Patient	Chi-squared Test	0.644	0.867
Year of Diagnosis	Patient	Wilcoxon Test	0.667	0.867
Ethnicity Category	Patient	Chi-squared Test	0.7	0.867
TMB (nonsynonymous)	Sample	Wilcoxon Test	0.735	0.867
Patient's Vital Status	Patient	Chi-squared Test	0.736	0.867
Last Communication Contact from Initial Pathologic Diagnosis Date	Patient	Wilcoxon Test	0.747	0.867
Mutation Count	Sample	Wilcoxon Test	0.756	0.867
Primary Diagnosis	Patient	Chi-squared Test	0.803	0.867
Disease Type	Patient	Chi-squared Test	0.803	0.867
Death from Initial Pathologic Diagnosis Date	Patient	Wilcoxon Test	0.937	0.973

Supplemental Table S3: Data Quality Control Summary

sample	library	raw_reads	raw_bases	clean_reads	clean_bases	error_rate	Q20	Q30	GC_pct
h295_SCR1	ERRA210018670-1a	94894112	14.23G	93568506	14.04G	0.03	96.86	92.23	49.7

h295_SCR2	ERRA210018671-1a	106704064	16.01G	105239296	15.79G	0.03	96.68	91.82	49.69
h295_SCR3	ERRA210018672-1a	82643362	12.4G	81366930	12.21G	0.03	96.91	92.27	49.63
h295_SCR4	ERRA210018673-1a	83065994	12.46G	81544432	12.23G	0.03	96.91	92.23	49.56
h295_E3_1	ERRA210018674-1a	93324308	14G	91942172	13.79G	0.03	96.93	92.32	49.38
h295_E3_2	ERRA210018675-1a	95445354	14.32G	94063908	14.11G	0.03	96.93	92.32	49.49
h295_E3_3	ERRA210018676-1a	77828132	11.67G	76585200	11.49G	0.03	96.97	92.42	49.73
h295_E3_4	ERRA210018677-1a	84913220	12.74G	83741324	12.56G	0.03	96.82	92.1	49.61
h295_E1KO	ERRA210018678-1a	91615566	13.74G	90057012	13.51G	0.03	96.78	92.02	49.65
h295_E2KO	ERRA210018679-1a	109927264	16.49G	108490704	16.27G	0.03	96.87	92.19	49.58
HAC_SCR1	ERRA210018680-1a	83887052	12.58G	82422734	12.36G	0.03	96.79	92	49.57
HAC_SCR2	ERRA210018681-1a	78093178	11.71G	76932056	11.54G	0.03	96.83	92.13	49.54
HAC_E3_1	ERRA210018682-1a	88338012	13.25G	87269924	13.09G	0.03	97	92.12	49.48
HAC_E3_2	ERRA210018683-1a	99334368	14.9G	98140034	14.72G	0.03	97.06	92.28	49.35
HAC_E3_3	ERRA210018684-1a	81877422	12.28G	80576604	12.09G	0.03	97.28	92.74	49.51

Establishing a functional KO of *ELAVL1* in NCI-H295R. A: DNA sequences of NCI-H295R Scr control and an *ELAVL1*-KO single cell clone. Red arrow marks insertion of one nucleotide, which was also proven by TIDE analyzes as seen below, resulting in a frame shift mutation. B: Total protein of NCI-H295R Scr control and six different NCI-H295R *ELAVL1*-KO single cell clones were compared in terms of ELAVL1 expression in Western blot. Histone H3 was stained as control. C: Immunofluorescence of NCI-H295R Scr (top), NCI-H295R mixed culture (KO-MC, middle) and an NCI-H295R *ELAVL1*-KO SCC (bottom). ELAVL1, which is enriched in the nucleus, was not detectable in *ELAVL1*-KO cells. Nucleus = Blue (DAPI); actin filaments = Green (Phalloidin); ELAVL1 = Red.

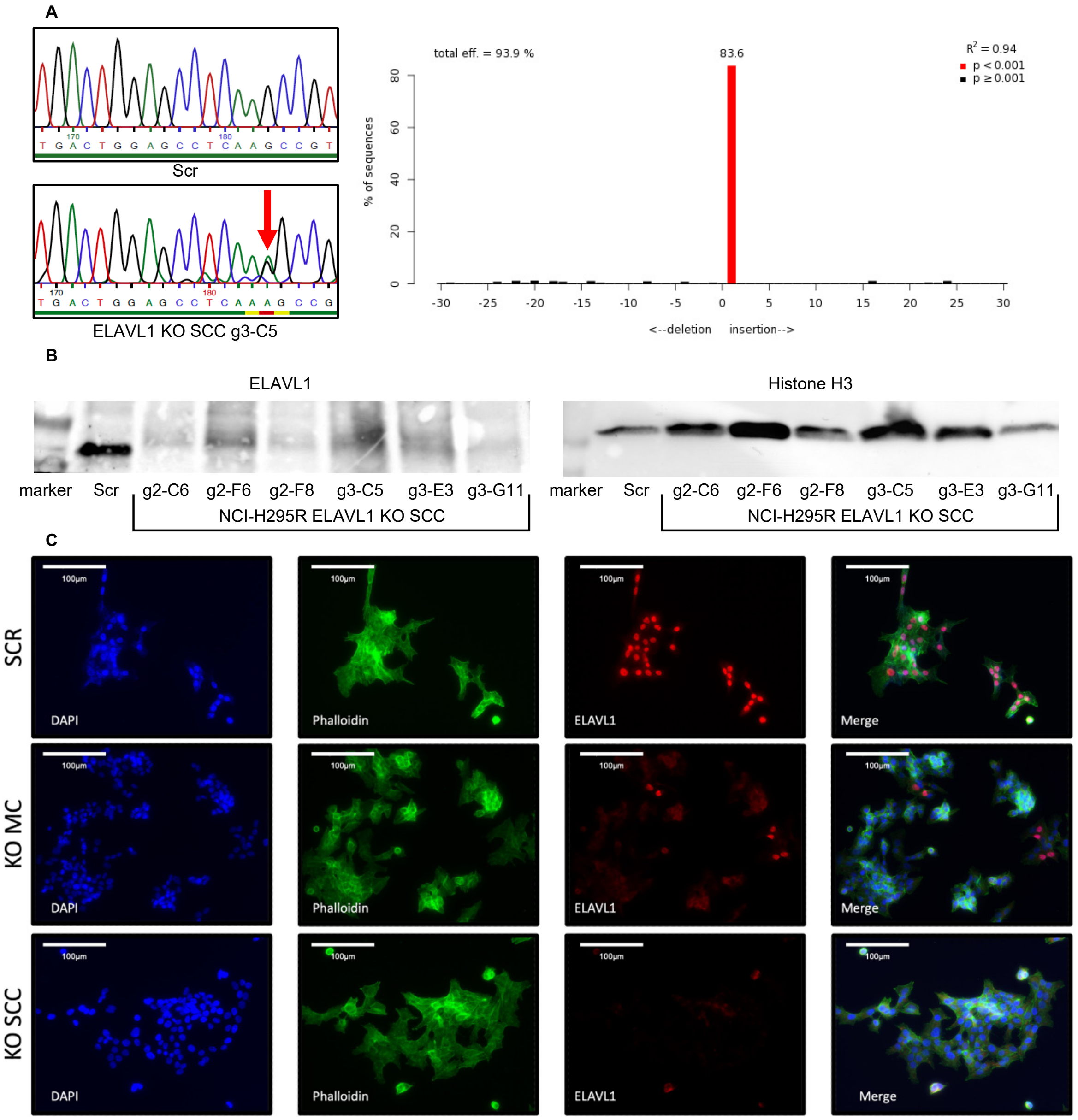

Supplemental methods

RNA Quantification and Qualification

RNA degradation and contamination was monitored on 1% agarose gels. RNA purity was checked using the NanoPhotometer® spectrophotometer (IMPLEN, CA, USA). RNA integrity

and quantitation were assessed using the RNA Nano 6000 Assay Kit of the Bioanalyzer 2100 system (Agilent Technologies, CA, USA).

Library Preparation for Transcriptome Sequencing

A total amount of 1 μg RNA per sample was used as input material for the RNA sample preparations. Sequencing libraries were generated using NEBNext® UltraTM RNA Library Prep Kit for Illumina® (New England Biolabs, Ipswich, MA, USA) following manufacturer's recommendations and index codes were added to attribute sequences to each sample. Briefly, mRNA was purified from total RNA using poly-T oligo-attached magnetic beads. Fragmentation was carried out using divalent cations under elevated temperature in NEBNext First Strand Synthesis Reaction Buffer (5X). First strand cDNA was synthesized using random hexamer primer and M-MuLV Reverse Transcriptase (RNase H-). Second strand cDNA synthesis was subsequently performed using DNA Polymerase I and RNase H. Remaining overhangs were converted into blunt ends via exonuclease/polymerase activities. After adenylation of 3' ends of DNA fragments, NEBNext Adaptor with hairpin loop structure were ligated to prepare for hybridization. In order to select cDNA fragments of preferentially 150~200 bp in length, the library fragments were purified with AMPure XP system (Beckman Coulter, Beverly, USA). Then 3 μl USER Enzyme (New England Biolabs, USA) was used with size-selected, adaptor ligated cDNA at 37 °C for 15 min followed by 5 min at 95 °C before PCR. Then PCR was performed with Phusion High-Fidelity DNA polymerase, Universal PCR primers and Index (X) Primer. At last, PCR products were purified (AMPure XP system) and library quality was assessed on the Agilent Bioanalyzer 2100 system.

Clustering and Sequencing

The clustering of the index-coded samples was performed on a cBot Cluster Generation System using PE Cluster Kit cBot-HS (Illumina, San Diego, CA, USA) according to the manufacturer's instructions. After cluster generation, the library preparations were sequenced on an Illumina platform and paired-end reads were generated.

Data Analysis and Quality Control

Raw data (raw reads) of FASTQ format were firstly processed through in-house scripts. In this step, clean data (clean reads) were obtained by removing reads containing adapter and poly-N sequences and reads with low quality from raw data. At the same time, Q20, Q30 and GC content of the clean data were calculated. All the downstream analyses were based on the clean data with high quality (59-61).

Mapping to Reference Genome

Reference genome and gene model annotation files were downloaded from genome website browser (NCBI/UCSC/Ensembl) directly. Paired-end clean reads were mapped to the reference genome using HISAT2 software. HISAT2 uses a large set of small GFM indexes that collectively cover the whole genome. These small indexes (called local indexes), combined with several alignment strategies, enable rapid and accurate alignment of sequencing reads (62-64.

Quantification

HTSeq was used to count the read numbers mapped of each gene, including known and novel genes. And then RPKM of each gene was calculated based on the length of the gene and reads count mapped to this gene. RPKM, Reads Per Kilobase of exon model per Million mapped reads, considers the effect of sequencing depth and gene length for the reads count at the same time, and is currently the most commonly used method for estimating gene expression levels (65, 66).

Differential Expression Analysis

Differential expression analysis between two conditions/groups (three biological replicates per condition) was performed using DESeq2 R package. DESeq2 provides statistical routines for determining differential expression in digital gene expression data using a model based on the negative binomial distribution. The resulting P values were adjusted using the Benjamini and Hochberg's approach for controlling the False Discovery Rate (FDR). Genes with an adjusted p value < 0.05 found byDESeq2 were assigned as differentially expressed (67).

Enrichment Analysis

A common way for searching shared functions among genes is to incorporate the biological knowledge provided by biological ontologies. Gene Ontology (GO) annotates genes to biological processes, molecular functions, and cellular components in a directed acyclic graph structure, and Kyoto Encyclopedia of Genes and Genomes (KEGG) annotates genes to pathway. KEGG is a database resource for understanding high-level functions and utilities of the biological system, such as the cell, the organism and the ecosystem, from molecular-level information, especially large-scale molecular datasets generated by genome sequencing and other high-through put experimental technologies (http://www.genome.jp/kegg/). We used KOBAS software to test the statistical enrichment of differential expression genes in KEGG pathways (68).

59. Cock, P. J., Fields, C. J., Goto, N., Heuer, M. L., and Rice, P. M. (2010) The Sanger FASTQ file format for sequences with quality scores, and the Solexa/Illumina FASTQ variants. Nucleic Acids Res 38, 1767-1771
60. Erlich, Y., Mitra, P. P., delaBastide, M., McCombie, W. R., and Hannon, G. J. (2008) Alta-Cyclic: a self-optimizing base caller for next-generation sequencing. Nat Methods 5, 679-682
61. Jiang, L., Schlesinger, F., Davis, C. A., Zhang, Y., Li, R., Salit, M., Gingeras, T. R., and Oliver, B. (2011) Synthetic spike-in standards for RNA-seq experiments. Genome Res 21, 1543-1551
62. Altschul, S. F., Madden, T. L., Schaffer, A. A., Zhang, J., Zhang, Z., Miller, W., and Lipman, D. J. (1997) Gapped BLAST and PSI-BLAST: a new generation of protein database search programs. Nucleic Acids Res 25, 3389-3402

63. Dobin, A., Davis, C. A., Schlesinger, F., Drenkow, J., Zaleski, C., Jha, S., Batut, P., Chaisson, M., and Gingeras, T. R. (2013) STAR: ultrafast universal RNA-seq aligner. Bioinformatics 29, 15-21
64. Finn, R. D., Tate, J., Mistry, J., Coggill, P. C., Sammut, S. J., Hotz, H. R., Ceric, G., Forslund, K., Eddy, S. R., Sonnhammer, E. L., and Bateman, A. (2008) The Pfam protein families database. Nucleic Acids Res 36, D281-288
65. Dillies, M. A., Rau, A., Aubert, J., Hennequet-Antier, C., Jeanmougin, M., Servant, N., Keime, C., Marot, G., Castel, D., Estelle, J., Guernec, G., Jagla, B., Jouneau, L., Laloe, D., Le Gall, C., Schaeffer, B., Le Crom, S., Guedj, M., Jaffrezic, F., and French StatOmique, C. (2013) A comprehensive evaluation of normalization methods for Illumina high-throughput RNA sequencing data analysis. Brief Bioinform 14, 671-683
66. Trapnell, C., Williams, B. A., Pertea, G., Mortazavi, A., Kwan, G., van Baren, M. J., Salzberg, S. L., Wold, B. J., and Pachter, L. (2010) Transcript assembly and quantification by RNA-Seq reveals unannotated transcripts and isoform switching during cell differentiation. Nat Biotechnol 28, 511-515
67. Anders, S., and Huber, W. (2010) Differential expression analysis for sequence count data. Genome Biol 11, R106
68. Kanehisa, M., and Goto, S. (2000) KEGG: kyoto encyclopedia of genes and genomes. Nucleic Acids Res 28, 27-30
